# Astrocytic Phenotypic Switching in Posterior Piriform Cortex Orchestrates Bone Cancer Pain–Depression Comorbidity via Purinergic–Noradrenergic Signaling

**DOI:** 10.1002/advs.202523150

**Published:** 2026-06-05

**Authors:** Jiang‐Ping Liu, Jun‐Han Zhang, Zhi‐Xuan Tan, Wei‐Bing Yan, Ya‐Ru Yuan, Yao Liu, Bing‐Di Wei, Meng‐Qi Ding, Zhuo‐Min Fu, Ying Liu, An‐Qi Wang, Lin Ma, Yong‐Hong Li, Er‐Qing Chai, Chao‐Jun Wei

**Affiliations:** ^1^ NHC Key Laboratory of Diagnosis and Therapy of Gastrointestinal Tumor Institute of Clinical Research and Translational Medicine Biobank and Bioinformation Engineering Research Center Gansu Provincial Hospital Lanzhou China; ^2^ The Third School of Clinical Medicine Lanzhou University Lanzhou China; ^3^ The First School of Clinical Medicine Gansu University of Chinese Medicine Lanzhou China; ^4^ The School of Public Health Gansu University of Chinese Medicine Lanzhou China; ^5^ Department of Psychiatry Qiqihar Medical University Qiqihar China; ^6^ KingMed School of Laboratory Medicine Guangzhou Medical University Guangzhou China; ^7^ Cerebrovascular Disease Centre Gansu Provincial Hospital Lanzhou China

**Keywords:** adenosine A2A receptor, astrocyte–neuron crosstalk, comorbid depression symptoms in bone cancer pain, norepinephrine transporter dysregulation, posterior piriform cortex

## Abstract

Bone cancer pain occurs in up to 70% of patients with skeletal metastases and is accompanied by depression, severely diminishing quality of life. Current pharmacological strategies provide only partial relief, and the neurobiological mechanisms linking nociceptive and affective processing remain poorly defined. Herein, in a mouse model of Lewis lung carcinoma, we identify a previously uncharacterized astrocyte–neuron signaling axis in the posterior piriform cortex that underlies bone cancer pain with comorbid depression. Through metabolomic, proteomic, and fiber photometry approaches, we show that astrocytic phenotypic transition from the neuroprotective A2 to the neurotoxic A1 state triggers metabolic reprogramming, downregulates ATP/adenosine/adenosine A2A receptor signaling, and upregulates the norepinephrine transporter, impairing noradrenergic transmission. Interventions targeting astrocyte phenotypes, including minocycline and *Lcn2* knockdown, normalized the posterior piriform cortex neurochemistry and alleviated both nociceptive and affective symptoms. These findings define a mechanistic framework in which astrocytic dysfunction orchestrates pain and depression, and highlight astrocyte‐directed strategies for treating cancer pain–depression comorbidity.

## Graphical abstract

Bone cancer pain and depression share a common origin: astrocytic A2‐to‐A1 transition in the posterior piriform cortex. This phenotypic shift disrupts the ATP–adenosine–A2AR–norepinephrine axis, simultaneously driving nociceptive and affective dysfunction. Targeting astrocyte phenotypes via minocycline or *Lcn2* knockdown rescues both symptoms, establishing astrocyte‐directed intervention as a promising dual‐target strategy for cancer pain–depression comorbidity.

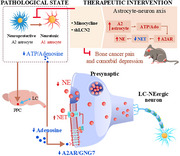

## 1 Introduction

Bone cancer metastasis represents a major clinical challenge, occurring in approximately 70% of patients with skeletal malignancies and profoundly impacting their quality of life [1, 2]. Beyond physical pain, these patients frequently experience comorbid depression symptoms (CDS), which further exacerbate other symptoms and compromise treatment outcomes [3]. Current pharmacological interventions, including opioids and serotonin–norepinephrine (NE) reuptake inhibitors, provide only partial relief and are often associated with adverse side effects [4, 5]. This clinical impasse underscores an urgent need to elucidate the neurobiological mechanisms that bridge nociceptive and affective processing, thereby enabling the development of targeted therapies for pain–depression comorbidity [6].

Research into bone cancer pain (BCP) has primarily focused on peripheral sensitization and spinal neuroplasticity [7, 8], approaches that insufficiently address the affective dimensions and fail to explain the high prevalence of CDS. While considerable progress has been made in mapping pain and depression circuits independently, supraspinal nodes integrating nociceptive and affective processing remain poorly characterized [9, 10]. The posterior piriform cortex (PPC), a paleocortical structure in the temporal lobe traditionally studied in the context of olfactory processing and seizures, represents a compelling candidate for pain–depression integration given its unique anatomical positioning [11, 12, 13]. Recent evidence implicates the PPC as a critical hub where sensory and emotional signals converge, potentially explaining the high comorbidity rates observed clinically [14]. The PPC has a distinctive connectivity, receiving direct thalamic nociceptive inputs and maintaining bidirectional connections with key pain processing and emotional regulation regions like the amygdala [15], locus coeruleus (LC) [15], anterior cingulate cortex [16], and the hypothalamus [17]. These intricate neural connections position the PPC as a key cortical node for pain–emotional integration and suggest that cortical‐level mechanisms could provide pivotal insights into developing effective treatments for pain‐associated depression.

Astrocytes, long regarded as supportive cells, are now recognized as active regulators of neural circuit function and behavior [18, 19, 20, 21], Under chronic pathological conditions, astrocytes undergo phenotypic transformation from the neuroprotective A2 to the neurotoxic A1 phenotype, characterized by morphological remodeling, transcriptional reprogramming, and disrupted adenosine 5'‐triphosphate (ATP)/adenosine (Ado) signaling [20, 22, 23, 24]. These changes destabilize neurotransmitter homeostasis, potentially contributing to both pain sensitization and affective disturbances [25, 26]. Recent studies have revealed a sophisticated astrocyte–neuron signaling mechanism involving NE and purinergic signaling [27, 28, 29]. Astrocytes are now recognized not only as NE responders but also as active orchestrators of neural circuit dynamics through NE‐induced calcium signaling, which triggers the release of ATP that is rapidly metabolized to Ado in the synaptic cleft [27, 28, 29]. This astrocyte–neuron feedback loop may underlie pain–depression comorbidity, particularly in brain regions such as the PPC that receive dense noradrenergic (NEergic) innervation [30, 31].

The astrocyte‐derived ATP/Ado signaling described above acts through distinct Ado receptor subtypes with region‐specific functions [32]. While spinal adenosine A1 receptors enhance inhibitory transmission and produce analgesia [33], adenosine A2A receptors (A2AR) exhibit context‐dependent effects, with reports of both facilitatory and inhibitory roles in modulating pain and emotion depending on cellular localization [34, 35, 36, 37, 38]. In the PPC, NEergic circuits interconnect with astrocyte networks, and A2AR is abundantly expressed. Accordingly, A2AR‐mediated modulation of NEergic transmission represents a particularly intriguing mechanism [39]. The NE transporter (NET) serves as the primary determinant of synaptic NE availability and is central to pain and mood regulation [40]. Recent advances have revealed a conserved NE–glia‐derived ATP/Ado regulatory loop that shapes behavioral states [27, 28, 29, 41]. However, whether astrocyte‐derived ATP/Ado acts on presynaptic A2AR to regulate NET activity in the PPC remains unknown, as does the potential contribution of this signaling axis to pain–depression comorbidity.

In this study, we identified a novel astrocyte‐to‐neuron ATP/Ado/A2AR/NET–NE signaling axis in the PPC that underlies BCP‐associated CDS. Mechanistically, BCP induced an A2‐to‐A1 astrocyte shift, reducing ATP/Ado signaling and diminishing A2AR‐mediated regulation of presynaptic NET, thereby impairing NE transmission and driving the comorbid phenotype. Pharmacological or genetic restoration of this pathway normalized ATP, Ado, and NE dynamics, alleviating both nociceptive and depression‐like behaviors. Conversely, A2AR antagonism, NET overexpression, NEergic depletion, and adrenergic receptor blockade, each targeting a discrete node of this axis, sufficed individually to phenocopy the pain–depression comorbidity in sham mice. Collectively, these findings highlight the PPC astrocyte–neuron circuit as a promising multi‐target therapeutic approach for managing depression comorbid with BCP.

## 2 Results

### 2.1 BCP Model Exhibits Comorbid Depression‐Like Behaviors and PPC Hyperactivation

**FIGURE 1:**
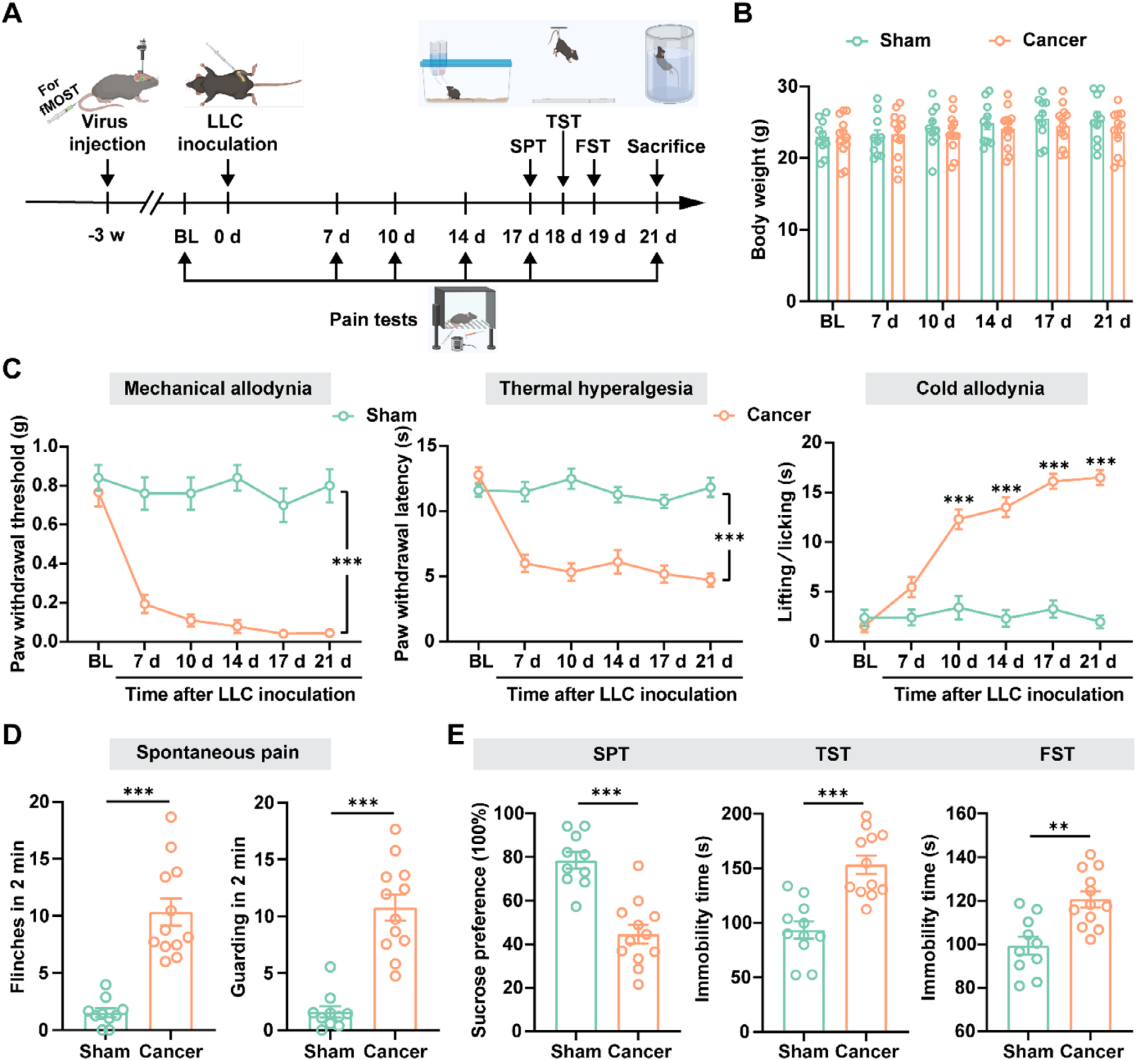
LLC inoculation induces progressive pain hypersensitivity and depression‐like behaviors in mice. (A) Experimental timeline: male C57BL/6J mice received virus injections 3 weeks before baseline via intravenous delivery (for fMOST imaging) or stereotaxic injection into PPC (for other experiments). At day 0, mice underwent intratibial inoculation of LLC cells or PBS (sham), followed by behavioral testing at the indicated time points. (B) Body weight remained comparable between sham and cancer groups throughout the experimental period (n = 12 mice per group). (C) Time course of nociceptive behaviors showing significant cancer‐induced mechanical allodynia (left), thermal hyperalgesia (middle), and cold allodynia (right) (*n* = 10–12 mice per group; ^***^ *p* < 0.001). (D) Spontaneous pain behaviors with increased flinching frequency and guarding frequency compared with sham controls (*n* = 10–12 mice per group; ^***^ *p* < 0.001). (E) Depression‐like behaviors in cancer mice assessed using SPT, TST, and FST (*n* = 10–12 mice per group; ^**^ *p* < 0.01, ^***^ *p* < 0.001). Significance was assessed using two‐way ANOVA with Bonferroni's post‐hoc test in B, C, and two‐tailed unpaired Student's *t*‐test in D and E. Data are presented as mean ± SEM. Source data and details of the statistical analyses are provided as a Source Data file. Created with BioRender.com.

To investigate the neural mechanisms underlying BCP with CDS, we established a BCP mouse model via intra‐femoral inoculation of Lewis lung carcinoma (LLC) cells. A comprehensive experimental paradigm was designed, integrating behavioral assessment, whole‐brain activity mapping, multi‐omics profiling, and neural circuit dissection (Figure 1A and Figure S1). Throughout the 21‐day observation period, body weight remained comparable between cancer and sham mice (Figure 1B), indicating that subsequent behavioral phenotypes were not attributable to general physical deterioration.

Following LLC inoculation, cancer mice developed progressive, multimodal pain hypersensitivity. Mechanical allodynia emerged as early as day 7 post‐inoculation, with paw withdrawal thresholds (PWT) significantly reduced relative to sham controls and persisting through day 21 (Figure 1C, left). Thermal hyperalgesia was similarly observed from day 7 to day 21, reflected by a significant reduction in paw withdrawal latency (PWL) (Figure 1C, middle), consistent with previous reports [7]. Cold allodynia (CA), assessed by acetone‐evoked lifting/licking duration, became evident from day 10 and progressively intensified through day 21 (Figure 1C, right). In addition, cancer mice displayed significantly elevated spontaneous pain behaviors, including increased flinching and guarding frequencies (Figure 1D).

We next evaluated whether BCP mice concurrently developed depression‐like behaviors. In the sucrose preference test (SPT), cancer mice exhibited a significantly reduced sucrose preference ratio, indicative of anhedonia—a core symptom of depression (Figure 1E, left). Cancer mice further exhibited markedly prolonged immobility times in both the tail suspension test (TST) and the forced swim test (FST), reflecting behavioral despair (Figure 1E, middle and right). Collectively, these data demonstrate that the BCP model reliably recapitulates the comorbidity of chronic pain and depression‐like behaviors.

**FIGURE 2:**
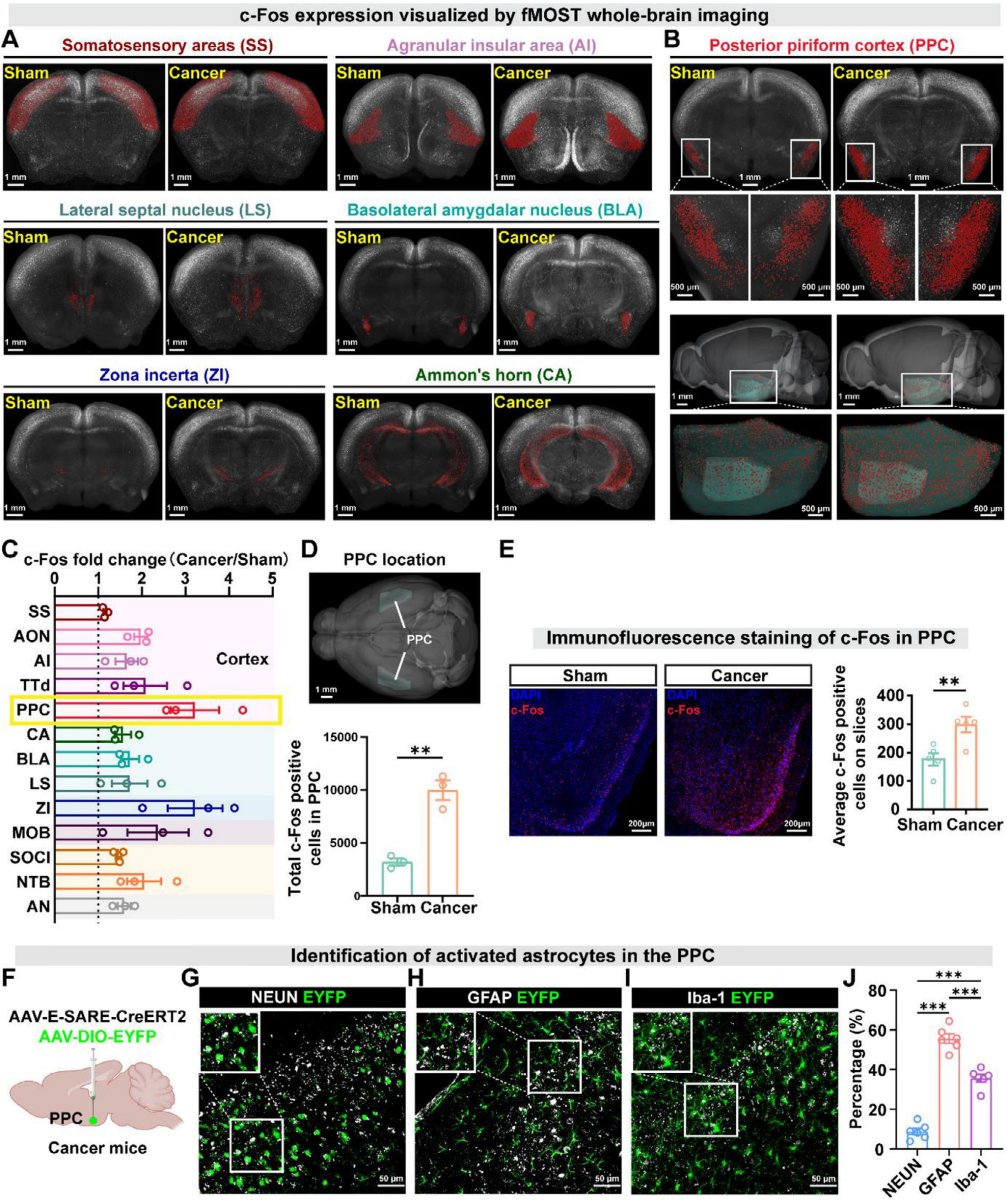
Whole‐brain fMOST c‐Fos mapping identifies astrocyte‐predominant PPC activation during BCP with CDS. (A) Representative coronal sections from fMOST imaging showing whole‐brain c‐Fos expression (white) with region‐specific c‐Fos‐positive cells pseudo‐colored in red for the somatosensory areas (SS), agranular insular area (AI), lateral septal nucleus (LS), basolateral amygdalar nucleus (BLA), zona incerta (ZI), and Ammon's horn (CA) in sham and cancer mice at day 21 post‐LLC inoculation. Scale bars, 1 mm. (B) fMOST coronal sections showing whole‐brain c‐Fos expression (white) with PPC‐specific c‐Fos‐positive cells highlighted in red at low (top; scale bars, 1 mm) and high (bottom; scale bars, 500 µm) magnification. Dashed boxes indicate the magnified regions. The PPC anatomical boundary is delineated in cyan in the magnified panels. (C) c‐Fos fold change (cancer/sham) across 13 brain regions quantified from fMOST data. The PPC (yellow highlight) exhibits the greatest fold change among all regions examined. The dashed line indicates a fold change of 1. AON, anterior olfactory nucleus; TTd, taenia tecta dorsal; MOB, main olfactory bulb; SOCl, superior olivary complex lateral; NTB, nucleus of the trapezoid body; AN, ansiform lobule (*n* = 3 mice per group). (D) Top: three‐dimensional rendering of the mouse brain indicating the anatomical location of the PPC (cyan). Scale bar, 1 mm. Bottom: quantification of total c‐Fos‐positive cells in the PPC from fMOST datasets (*n* = 3 mice per group; ^**^ *p* < 0.01). (E) Left and middle: representative immunofluorescence images of c‐Fos (red) and DAPI (blue) in the PPC of sham and cancer mice. Scale bars, 200 µm. Right: quantification of average c‐Fos‐positive cells per section (*n* = 5 mice per group; ^**^ *p* < 0.01). (F) Schematic of the activity‐dependent viral labeling strategy. AAV‐E‐SARE‐CreERT2 and AAV‐DIO‐EYFP were co‐injected into the PPC of cancer mice to identify activated cell types. (G–I) Representative immunofluorescence images of EYFP (green) co‐stained with (G) NeuN (neurons, white), (H) GFAP (astrocytes, white), and (I) Iba‐1 (microglia, white) in the PPC. Scale bars, 50 µm. Insets show higher‐magnification views of the boxed regions. (J) Quantification of the percentage of EYFP‐positive cells co‐labeled with NeuN, GFAP, or Iba‐1 (*n* = 6 slices/3 mice per group; ^***^ *p* < 0.001). Significance was assessed using a two‐tailed unpaired Student's *t*‐test in (C–E), and one‐way ANOVA with Bonferroni's post‐hoc test in (J). Data are presented as mean ± SEM. Exact *p*‐values, degrees of freedom, and pairwise comparisons are provided in the Source Data file. Created with BioRender.com.

To identify brain regions activated during BCP‐associated pain–depression comorbidity, we utilized fluorescence micro‐optical sectioning tomography (fMOST) for whole‐brain mapping of c‐Fos expression, a well‐established marker of cellular activation. Three weeks prior to LLC inoculation, a c‐Fos‐driven reporter virus was delivered via lateral tail vein injection to enable whole‐brain labeling (Figure 1A). We performed comparative analysis of c‐Fos expression across multiple brain regions, including the somatosensory areas (SS), agranular insular area (AI), lateral septal nucleus (LS), basolateral amygdalar nucleus (BLA), zona incerta (ZI), and Ammon's horn (CA). The results revealed diverse differences in c‐Fos expression between cancer and sham mice (Figure 2A). Notably, the posterior piriform cortex (PPC) exhibited a strikingly higher c‐Fos signal in cancer mice (Figure 2B). Quantitative fold‐change analysis (cancer/sham) across 13 brain regions further highlighted the PPC as the region with the most prominent activation (Figure 2C). Consistently, the total number of c‐Fos‐positive cells in the PPC was significantly higher in cancer mice than in sham controls (Figure 2D). To independently validate this observation, we performed immunofluorescence staining on PPC tissue sections, which confirmed a significantly higher c‐Fos‐positive cell density in cancer mice (Figure 2E).

To determine the cellular identity of the activated population in the PPC, we employed an activity‐dependent sparse labeling strategy. A cocktail of AAV‐E‐SARE‐CreERT2 and AAV‐DIO‐EYFP was co‐injected into the PPC, allowing 4‐hydroxytamoxifen‐induced EYFP expression selectively in recently activated cells (Figure 2F). Co‐immunostaining of EYFP with cell‐type‐specific markers revealed that only a small fraction of EYFP‐positive cells co‐localized with the neuronal marker NeuN (Figure 2G). In contrast, the majority of EYFP‐labeled cells co‐expressed the astrocytic marker glial fibrillary acidic protein (GFAP) (Figure 2H), while a substantial subset also overlapped with the microglial marker Iba‐1 (Figure 2I). Quantification confirmed that GFAP‐positive astrocytes accounted for approximately 55% of all activated cells in the PPC, significantly exceeding the proportions of Iba‐1‐positive microglia (∼35%) and NeuN‐positive neurons (∼10%) (Figure 2J). These findings suggest that astrocytes, rather than neurons or microglia, represent the predominant activated cell type in the PPC during BCP, implicating PPC astrocytic activation as a potential key mediator of cancer‐induced pain–depression comorbidity.

### 2.2 BCP Induces PPC Astrocyte Activation and A2‐to‐A1 Transition During CDS

**FIGURE 3:**
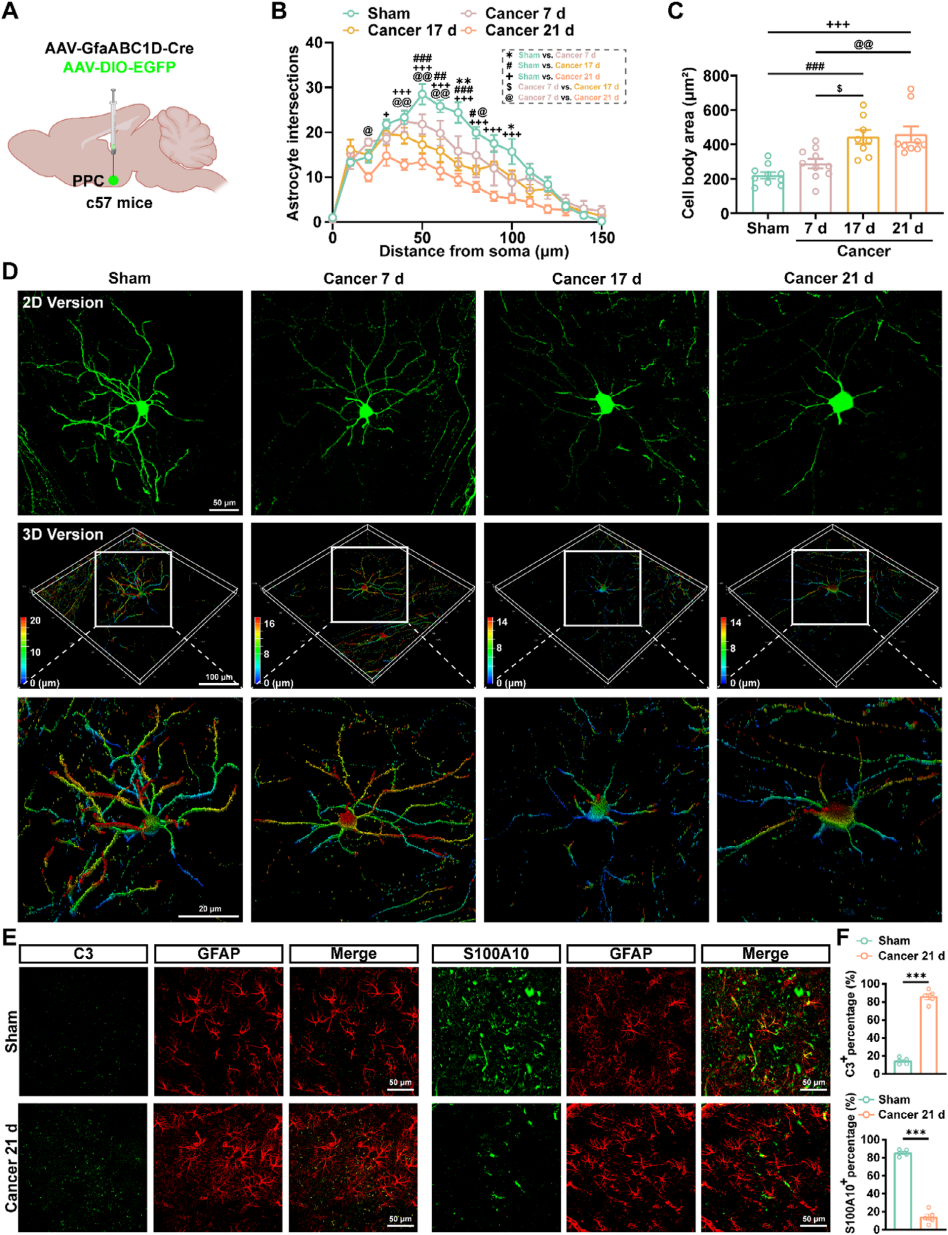
Progressive astrocytic hypertrophy and A1‐type neurotoxic polarization in the PPC during BCP‐induced CDS. (A) Schematic of the astrocyte‐specific labeling strategy. AAV‐GfaABC1D‐Cre and AAV‐DIO‐EGFP were co‐injected into the PPC of C57BL/6J mice to selectively express EGFP in astrocytes. (B) Sholl analysis of astrocyte morphological complexity plotted as the number of process intersections versus distance from the soma in sham, cancer 7 d, cancer 17 d, and cancer 21 d groups (*n* = 8–10 cells per group; ^*^ *p* < 0.05, ^**^ *p* < 0.01 for sham vs. cancer 7 d; *^#^p* < 0.05, *^##^p* < 0.01, *^###^p* < 0.001 for sham vs. cancer 17 d; *^+^p* < 0.05, *^+++^p* < 0.001 for sham vs. cancer 21 d; *^@^p* < 0.05, *^@@^p* < 0.01 for cancer 7 d vs. cancer 21 d). (C) Quantification of astrocyte cell body area (µm^2^) across groups (*n* = 8–10 cells per group; *^###^p* < 0.001 for sham vs. cancer 17 d; *^+++^p* < 0.001 for sham vs. cancer 21 d; *^$^p* < 0.05 for cancer 7 d vs. cancer 17 d; *^@@^p* < 0.01 for cancer 7 d vs. cancer 21 d). (D) Top row (two‐dimensional version): representative confocal images of individual EGFP‐labeled astrocytes in each group. Scale bar, 50 µm. Middle row (three‐dimensional version): three‐dimensional reconstructions of astrocytes pseudo‐colored by process diameter (µm) as indicated by the color scales at the bottom‐left of each panel. Boxed regions are magnified below. Scale bar, 100 µm. Bottom row: magnified views of the boxed regions from the three‐dimensional reconstructions. Scale bar, 20 µm. (E) Representative immunofluorescence images of C3 (A1 reactive astrocyte marker) with GFAP co‐staining (left) and S100A10 (A2 reactive astrocyte marker) with GFAP co‐staining (right) in the PPC of sham and cancer 21 d mice. Scale bars, 50 µm. (F) Quantification of the percentage of C3^+^ cells (top) and S100A10^+^ cells (bottom) among GFAP^+^ astrocytes in the PPC of sham and cancer 21 d mice (*(n =* 5 slices/3 mice per group; ^***^ *p* < 0.001). Significance was assessed using two‐way ANOVA with Bonferroni's post‐hoc test in (B), one‐way ANOVA with Bonferroni's post‐hoc test in (C), and two‐tailed unpaired Student's *t*‐test in (F). Data are presented as mean ± SEM. Exact *p*‐values, degrees of freedom, and pairwise comparisons are provided in the Source Data file. Created with BioRender.com.

Having established that astrocytes are the principal activated cell type in the PPC, we next investigated whether these cells undergo morphological remodeling during BCP progression. To visualize individual astrocyte morphology with high fidelity, we performed astrocyte‐specific sparse labeling by co‐injecting AAV‐GfaABC1D‐Cre and AAV‐DIO‐EGFP into the PPC of C57BL/6J mice (Figure 3A).

High‐resolution confocal imaging at multiple time points after tumor inoculation revealed progressive structural alterations in PPC astrocytes. Sholl analysis demonstrated a time‐dependent reduction in arborization complexity, with significantly decreased branching intersections across distances from the soma in cancer mice compared with sham controls (Figure 3B). Quantification of soma area further revealed significant hypertrophy at 7, 17, and 21 days post‐inoculation, with maximal enlargement at day 21 (Figure 3C). Consistent with these quantitative changes, two‐dimensional maximum intensity projections and three‐dimensional volume renderings showed that astrocytes in cancer mice exhibited enlarged cell bodies, thickened primary processes, and simplified branching, in stark contrast to the highly ramified morphology with extensive fine processes seen in sham controls (Figure 3D). Together, these morphological features, including soma hypertrophy coupled with process retraction and simplification, are hallmarks of reactive astrogliosis and suggest that PPC astrocytes become progressively activated during cancer‐induced pain and depression comorbidity.

Reactive astrocytes can be classified into neurotoxic A1 and neuroprotective A2 subtypes, with A1 astrocytes known to promote neuroinflammation [42] and contribute to depression pathogenesis [43]. The morphological changes observed above, including soma hypertrophy and process simplification, are characteristic features associated with the phenotypic transition from the A2 to the A1 state [44]. To directly test whether such a subtype switch occurs in BCP, we examined astrocyte subtype composition during cancer progression by co‐immunostaining PPC sections with GFAP and subtype‐specific markers. In sham controls, we observed extensive co‐localization between the A2 marker S100A10 and GFAP, whereas the A1 marker C3 showed minimal overlap with GFAP, indicating a predominance of the A2 subtype under physiological conditions. Strikingly, in cancer mice at 21 days, this pattern was dramatically reversed: C3 and GFAP exhibited extensive co‐localization, whereas S100A10 and GFAP showed minimal overlap, demonstrating a clear shift from the A2 to the A1 phenotype during pain and depression comorbidity (Figure 3E). Quantitative analysis confirmed that the proportion of C3^+^ astrocytes was significantly elevated in cancer mice, while the proportion of S100A10^+^ astrocytes was correspondingly reduced compared with sham controls (Figure 3F).

Collectively, these data demonstrate that PPC astrocytes undergo concurrent morphological remodeling and phenotypic transformation from the neuroprotective A2 subtype to the neurotoxic A1 subtype during BCP, establishing astrocyte dysfunction in the PPC as a critical cellular substrate in the pathogenesis of cancer‐induced pain and depression comorbidity.

### 2.3 A2‐to‐A1 Astrocytic Transformation Drives Concurrent Purinergic and NEergic Signaling Deficits Underlying Pain–Depression Comorbidity

**FIGURE 4:**
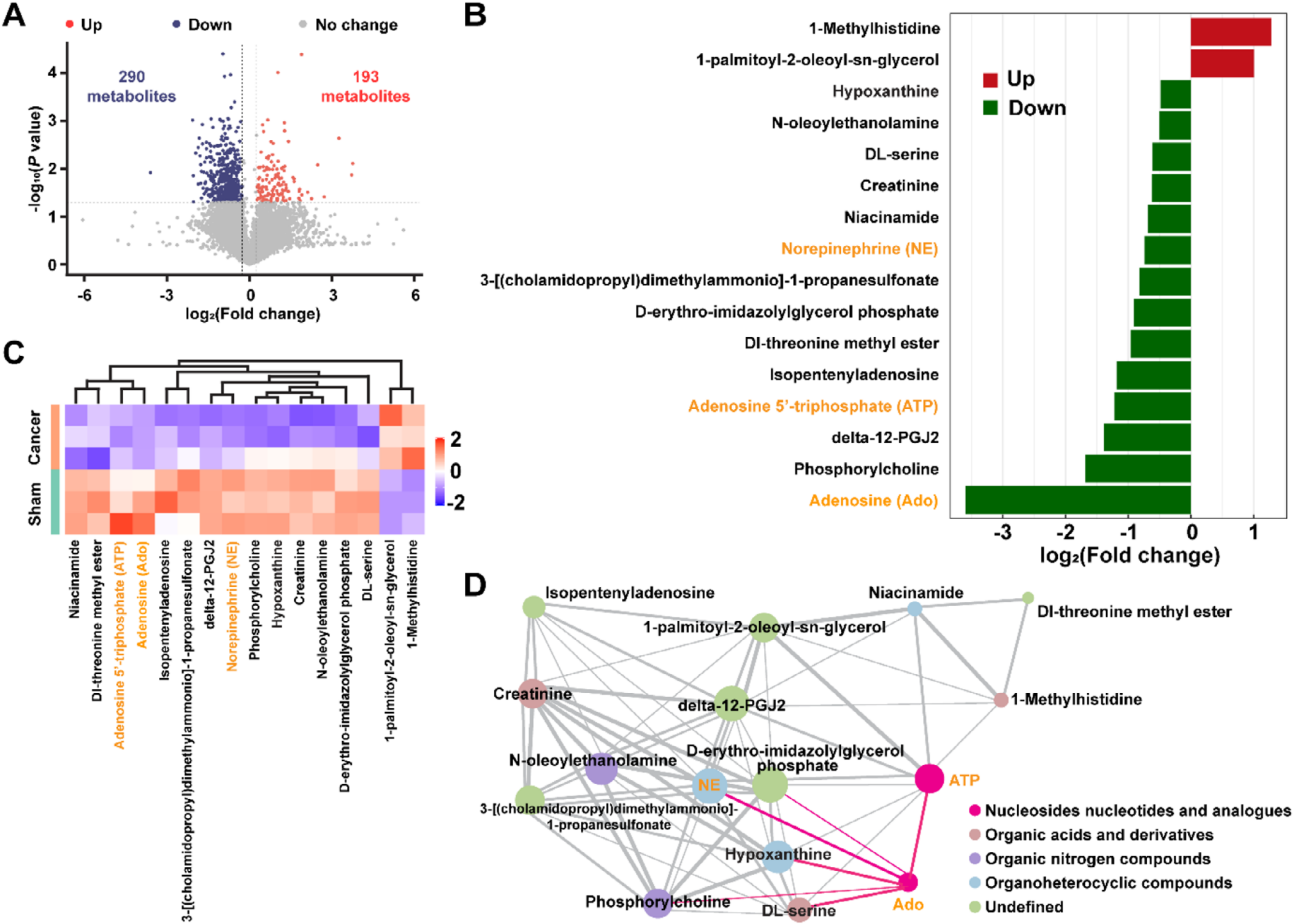
Untargeted metabolomics profiling reveals significant alterations in purinergic and NEergic metabolites in the PPC of cancer mice. (A) Volcano plot of differentially expressed metabolites in the PPC between the sham and cancer groups. Red, upregulated (193 metabolites); blue, downregulated (290 metabolites); gray, no significant change. Thresholds: |log_2_(FC)| > 1.2, *p* < 0.05 (*n* = 3 samples per group, each pooled from 4 mice). (B) Log_2_(FC) of selected differentially expressed metabolites. Red and green bars denote upregulated and downregulated metabolites, respectively. Ado, ATP, and NE are highlighted in orange (*n* = 3 samples per group, each pooled from 4 mice). (C) Heat map of differentially expressed metabolites across sham and cancer groups (*n* = 3 samples per group, each pooled from 4 mice). (D) Correlation network highlighting relationships among key metabolites (*n* = 3 samples per group, each pooled from 4 mice). Metabolites were identified using untargeted LC–MS/MS. Differential expression was determined by two‐tailed Student's *t*‐test with Benjamini–Hochberg FDR correction in (A–C); correlations in (D) were computed using Pearson's correlation coefficient. Exact *p*‐values and FDR‐adjusted *q*‐values are provided in the Source Data file.

Astrocytes critically regulate brain metabolism and neurotransmitter homeostasis [28, 45]. To determine whether the A2‐to‐A1 shift reshapes the neurochemical milieu of the PPC, we performed untargeted metabolomic profiling. This analysis identified 483 significantly dysregulated metabolites in comorbid mice relative to sham controls, with 193 upregulated and 290 downregulated (Figure 4A). Among the most strongly downregulated were ATP, Ado, and NE (Figure 4B), all key neuromodulators operating at the interface of pain processing and affective regulation [46]. Beyond these individual changes, hierarchical clustering of the full metabolite profile cleanly separated comorbid from sham samples (Figure 4C), confirming that these alterations constitute a coherent metabolic signature. Building on this, interaction network analysis positioned ATP and Ado as hub nodes forming a prominent signaling axis with NE (Figure 4D), implicating this axis as a potential molecular link between nociceptive and affective processing. Given that A1 astrocytes display impaired ATP release and altered Ado metabolism relative to A2 astrocytes [47], these findings suggest that the A2‐to‐A1 shift may directly drive purinergic signaling deficits in the PPC.

**FIGURE 5:**
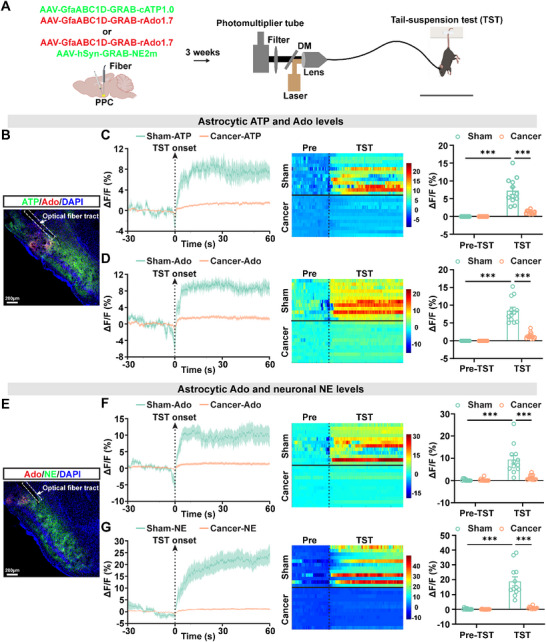
Fiber photometry reveals diminished astrocytic ATP, Ado, and neuronal NE release in the PPC during despair behavior in BCP‐CDS mice. (A) Schematic of the experimental strategy. AAV‐GfaABC1D‐GRAB‐cATP1.0 with AAV‐GfaABC1D‐GRAB‐rAdo1.7, or AAV‐GfaABC1D‐GRAB‐rAdo1.7 with AAV‐hSyn‐GRAB‐NE2m, were co‐injected into the PPC, followed by optical fiber implantation. After 3 weeks, fiber photometry signals were recorded during the TST. (B) Representative confocal image showing co‐expression of the ATP sensor (green) and Ado sensor (red) with DAPI (blue) in the PPC. The dashed line indicates the cannula fiber tract. Scale bar, 200 µm. (C) Astrocytic ATP dynamics during TST. Left, average ΔF/F (%) traces aligned to TST onset (dashed line) for sham and cancer groups. Middle, heatmaps of individual trials during Pre‐TST and TST periods. Right, quantification of mean ΔF/F during Pre‐TST and TST epochs (*n* = 12 mice per group; ^***^ *p* < 0.001). (D) Astrocytic Ado dynamics recorded simultaneously with ATP in the same cohort as (C). Displayed as in (C) (*n* = 12 mice per group; ^***^ *p* < 0.001). (E) Representative confocal image showing co‐expression of the Ado sensor (red) and NE sensor (green) with DAPI (blue) in the PPC. Scale bar, 200 µm. (F) Astrocytic Ado dynamics during TST in a separate cohort co‐expressing the Ado and NE sensors. Displayed as in (C) (*n* = 12 mice per group; ^***^ *p* < 0.001). (G) Neuronal NE dynamics recorded simultaneously with Ado in the same cohort as (F). Displayed as in (C) (*n* = 12 mice per group; ^***^ *p* < 0.001). Significance was assessed using two‐way ANOVA with Bonferroni's post‐hoc test in (C), (D), (F), and (G). Data are presented as mean ± SEM. Exact *p*‐values, degrees of freedom, and pairwise comparisons are provided in the Source Data file. Created with BioRender.com.

To monitor neurotransmitter dynamics in vivo, we employed genetically encoded GRAB fluorescent sensors: AAV‐GfaABC1D‐GRAB‐cATP1.0 and AAV‐GfaABC1D‐GRAB‐rAdo1.7 for astrocytic ATP and Ado, together with AAV‐hSyn‐GRAB‐NE2m for neuronal NE (Figure 5A). Two sensor combinations were co‐injected into the PPC for simultaneous dual‐color fiber photometry in freely behaving mice. For the ATP‐Ado pair, histological examination confirmed accurate targeting and faithful sensor expression at the injection site (Figure 5B), and during the TST, sham mice exhibited robust increases in astrocytic ATP and Ado, whereas cancer mice displayed markedly blunted responses in both signals (Figure 5C,D; both *p* < 0.001). For the Ado‐NE pair, histological verification likewise confirmed proper sensor distribution across astrocytic and neuronal compartments (Figure 5E), and TST recordings revealed correspondingly attenuated Ado and neuronal NE transients in cancer mice relative to sham controls (Figure 5F,G; both *p* < 0.001).

To validate the recording platform beyond functional outputs, we systematically characterized sensor expression at multiple levels. Bulk‐tissue analyses by RT‐PCR (Figure S2A) and western blot (Figure S2B) revealed no statistically significant between‐group differences, although small non‐significant trends at the mRNA level (4.9% lower GFP and 5.6% lower mApple in cancer animals; both *p* > 0.3), combined with the limited power of our pooled‐sample cohort (n = 6 pooled samples per group, drawn from 24 animals total), leave open the possibility of modest differences (≤ ∼20%). Per‐astrocyte sensor content assessed by GFAP co‐immunostaining was likewise indistinguishable between groups (Figure S2C–H), although a modest shift in soma‐to‐process intensity distribution was observed (see Discussion for limitations). As an orthogonal, sensor‐free validation, we performed targeted LC‐MS/MS quantification of ATP and Ado in PPC tissue using authenticated analytical standards, which confirmed significant reductions of both metabolites in cancer mice (Figure S3A, B; both *p* < 0.001), consistent with the untargeted metabolomic findings (Figure 4B) and establishing tissue‐level purinergic deficits independently of sensor performance.

An analogous pattern of attenuated neurotransmitter dynamics emerged during von Frey mechanical stimulation (2.0 g) (Figure S4A), where cancer mice showed significantly lower astrocytic ATP, Ado, and neuronal NE transients than sham controls (Figure S4B–E; all *p* < 0.001). Because the A2‐to‐A1 transformation constitutes a stable structural alteration, we reasoned that these signaling deficits should persist as tonic impairments beyond pain‐ and depression‐specific contexts. Consistent with this prediction, cancer mice exhibited markedly attenuated behavior‐locked neurotransmitter transients during spontaneous home‐cage behaviors such as grooming, sniffing, and rearing (Figure S5A–E; all *p* < 0.05), and failed to mount speed‐dependent neurotransmitter responses during the accelerating rotarod task (Figure S5F–I; all *p* < 0.001). The pervasive nature of these deficits aligns with the constitutive character of A1 astrocytic transformation, but raises the question of whether they bear preferential relevance to the comorbid phenotype or instead reflect nonspecific signaling degradation.

To resolve this, we asked whether A1 astrocyte polarization and its downstream signaling molecules track the severity of behavioral deficits within the pathological state itself, rather than merely reflecting differences between sham and cancer animals. We therefore performed Pearson correlation analyses restricted to cancer‐group mice. Within this group, the proportion of A1 astrocytes correlated strongly with both pain‐related measures (PWT, PWL, CA, SP) and depression‐related outcomes (SPT, TST, FST) (Figure S6A). Levels of astrocytic ATP, Ado, and neuronal NE showed similar patterns, correlating with most pain‐related (PWT, PWL, CA) and depression‐related indices (Figure S6B–D). These within‐group correlations indicate that the magnitude of astrocytic and neurochemical alterations scales with behavioral severity on a per‐animal basis, pointing to genuine biological coupling rather than passive group separation. One notable exception was SP, which correlated strongly with A1 polarization but showed little relationship with neurotransmitter levels, raising the possibility that spontaneous pain is driven more directly by the astrocytic phenotypic transformation itself rather than by deficits in the downstream purinergic and NEergic signaling. Apart from this case, all four variables consistently showed their strongest associations with indices reflecting combined pain and affective dysfunction, consistent with the idea that the astrocyte‐to‐neuron signaling axis from ATP/Ado to NE underlies the comorbid phenotype rather than pain or depression in isolation.

Collectively, these results establish a mechanistic cascade in which BCP‐induced A2‐to‐A1 astrocytic transformation leads to concurrent deficits in astrocyte‐derived purinergic signaling and neuronal NEergic transmission in the PPC. Although these impairments manifest tonically across diverse behavioral contexts, they are most strongly associated with combined pain–affective dysfunction, identifying the astrocytic ATP/Ado–neuronal NE signaling axis as a critical neurochemical substrate of cancer‐induced pain–depression comorbidity.

### 2.4 Data‐Independent Acquisition Proteomics Reveals Dysregulation of Multiple Neurotransmitter Signaling Pathways in the PPC of Comorbid Mice

The concurrent A1 astrocytic polarization and purinergic–NEergic deficits characterized above prompted us to investigate the upstream protein‐level mechanisms governing these neurochemical changes. To this end, we performed data‐independent acquisition (DIA) proteomic profiling of PPC tissues from sham and cancer‐bearing mice.

**FIGURE 6:**
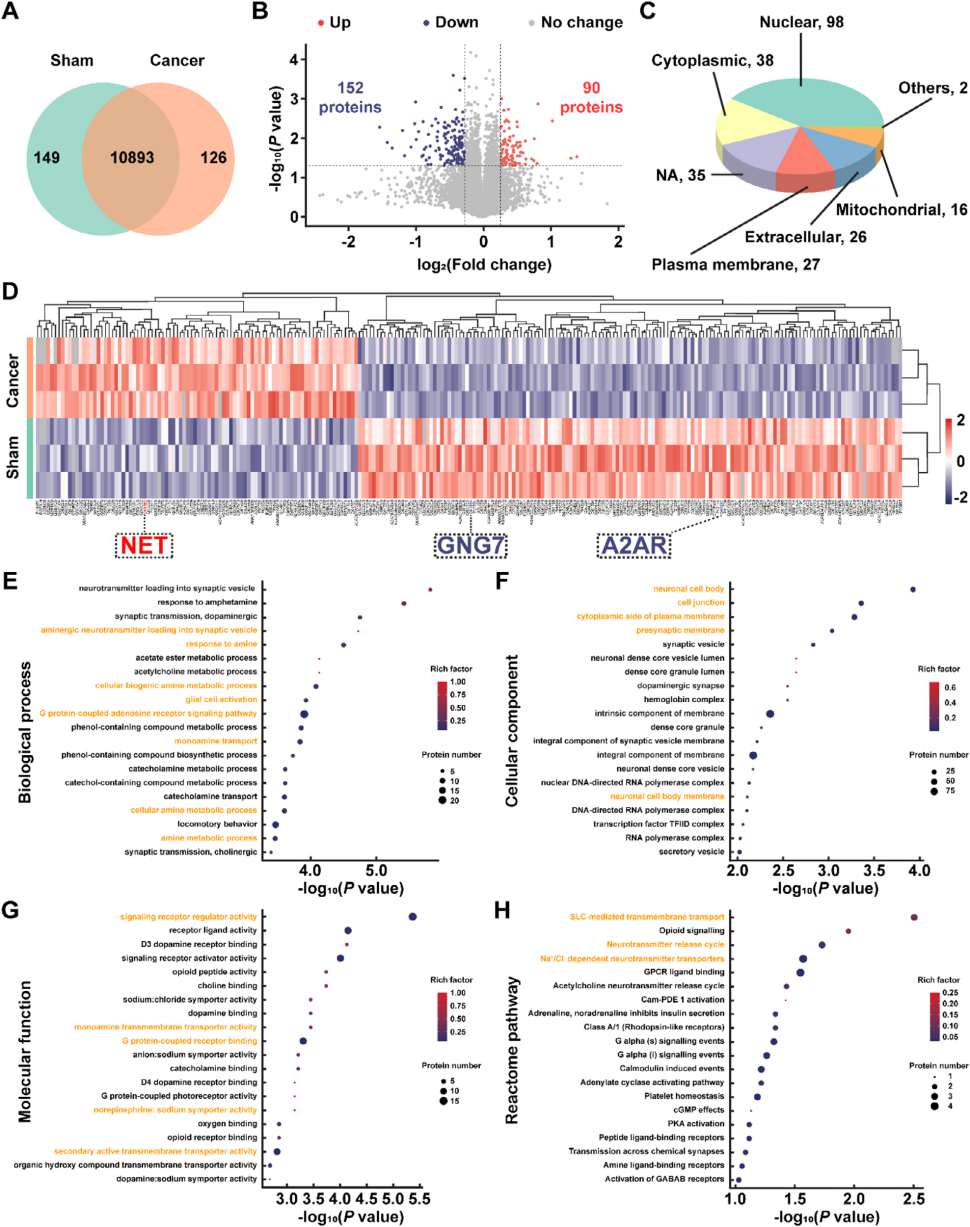
DIA‐based proteomic identifies dysregulated purinergic and NEergic signaling‐related proteins in the PPC of BCP‐CDS mice. (A) Venn diagram showing overlap of proteins identified in the sham and cancer groups. A total of 10,893 proteins were commonly detected (*n* = 3 pooled samples per group, 4 mice per sample). (B) Volcano plot of differentially expressed proteins (DEPs). Red, upregulated (90 proteins); blue, downregulated (152 proteins); gray, no significant change. Thresholds: |log_2_(FC)| > 1.2 and *p* < 0.05 (*n* = 3 pooled samples per group, each pooled from 4 mice). (C) Pie chart of the subcellular localization distribution of DEPs. (D) Heatmap with unsupervised hierarchical clustering of DEPs. Color scale represents z‐score–normalized expression. NET, GNG7, and A2AR are highlighted in black boxes (*n* = 3 pooled samples per group, each pooled from 4 mice). (E–G) Gene Ontology enrichment analysis of DEPs for biological process (E), cellular component (F), and molecular functions (G). Bubble size indicates protein number; color represents the Rich factor. Terms related to purinergic and NEergic signaling are highlighted in colored text. (H) Reactome pathway enrichment analysis of DEPs. Displayed as in (E–G). DEPs were identified by DIA‐based label‐free quantitative proteomics. Differential expression was determined using two‐tailed Student's *t*‐test with Benjamini–Hochberg FDR correction in (B) and (D); enrichment significance in (E–H) was assessed using Fisher's exact test with FDR correction. Data are presented as mean ± SEM where applicable. Exact *p*‐values and FDR‐adjusted *q*‐values are provided in the Source Data file.

A total of 11 168 proteins were quantified across both groups, with high overlap (10 893 shared) confirming consistent proteomic coverage (Figure 6A). Differential expression analysis identified 242 significantly altered proteins (*p* < 0.05, |log_2_FC| > 1.20), comprising 152 downregulated and 90 upregulated in cancer mice (Figure 6B). Subcellular localization analysis showed that differentially expressed proteins were predominantly nuclear (98, 40.5%) and cytoplasmic (38, 15.7%), with notable representation on the plasma membrane (27, 11.2%), in the extracellular space (26, 10.7%), and in mitochondria (16, 6.6%) (Figure 6C). Hierarchical clustering clearly separated the two groups, confirming distinct proteomic signatures in comorbid mice (Figure 6D).

Critically, among the differentially expressed proteins, we identified direct regulators of purinergic and NEergic neurotransmission: NET was significantly upregulated, whereas GNG7 and A2AR were markedly downregulated (Figure 6D). Upregulation of NET, the primary synaptic NE reuptake transporter, provides a parsimonious molecular explanation for the diminished NE tone detected by GRAB‐NE photometry, while downregulation of A2AR and its coupled signaling partner GNG7 points to impaired adenosinergic receptor signaling that may both result from and further exacerbate the purinergic deficits documented above.

Gene Ontology enrichment analysis corroborated these individual findings at the systems level. Differentially expressed proteins were significantly enriched in biological processes related to neurotransmitter loading into synaptic vesicles, monoamine metabolism, and G protein‐coupled adenosine receptor signaling (Figure 6E); in cellular components including neuronal dense‐core vesicles, synaptic vesicles, and presynaptic membranes (Figure 6F); and in molecular functions encompassing monoamine transmembrane transporter activity, signaling receptor regulation, and G protein‐coupled receptor binding (Figure 6G). Reactome pathway analysis further confirmed involvement of neurotransmitter release cycle, Na^+^/Cl^−^‐dependent neurotransmitter transporters, and solute carrier‐mediated transmembrane transport pathways (Figure 6H).

Overall, the proteomic data point to dysregulation of synaptic proteins governing purinergic and monoaminergic neurotransmission, providing a molecular framework linking A1 astrocytic transformation to the downstream neurotransmitter deficits in the PPC of comorbid mice. In particular, NET upregulation and A2AR/GNG7 downregulation emerge as candidate molecular substrates bridging BCP with CDS.

### 2.5 Molecular Validation of Key Players and Their Interaction Networks Within the LC–PPC NEergic Circuit

The proteomic analysis above suggested NET, A2AR, and GNG7 as candidate molecular drivers of purinergic–NEergic dysfunction in the PPC. To move beyond correlative proteomics, we undertook multi‐level validation of these targets and mapped the neural circuit through which they operate.

**FIGURE 7:**
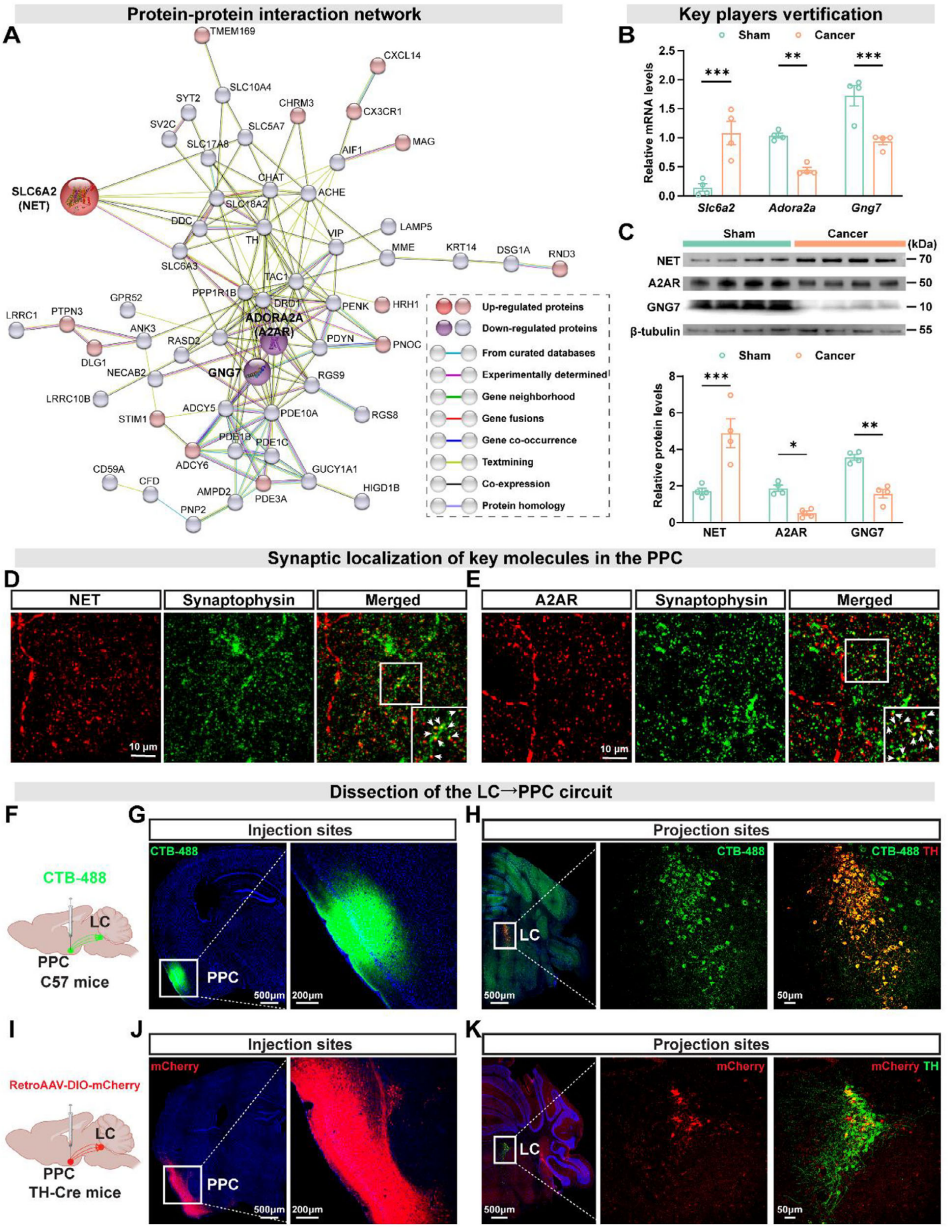
Network analysis identifies key purinergic and NEergic regulators in the PPC receiving LC‐derived NEergic projections. (A) Protein‐protein interaction (PPI) network of DEPs constructed via STRING. Red and purple nodes denote upregulated and downregulated proteins, respectively. Edge colors indicate interaction evidence types. SLC6A2 (NET), ADORA2A (A2AR), and GNG7 are highlighted as hub nodes. (B) qRT‐PCR validation of *Slc6a2*, *Adora2a*, and *Gng7* mRNA levels in the PPC (*n* = 4 pooled samples per group, each pooled from 4 mice; ^**^ *p* < 0.01, ^***^ *p* < 0.001). (C) Representative western blots (top) and quantification (bottom) of NET, A2AR, and GNG7 in the PPC. β‐tubulin served as loading control (*n* = 4 pooled samples per group, each pooled from 4 mice; ^*^ *p* < 0.05, ^**^ *p* < 0.01, ^***^ *p* < 0.001). (D, E) Immunofluorescence co‐staining of NET (D) or A2AR (E) with synaptophysin (presynaptic marker) in the PPC. Arrowheads indicate co‐localized puncta. Scale bars, 10 µm (*n* = 5 slices/3 mice per group). (F–H) Retrograde tracing with CTB‐488 injected into the PPC of C57BL/6J mice. (F) Schematic of the injection strategy. (G) Injection site in the PPC. (H) CTB‐488^+^ neurons in the LC co‐labeled with TH. Scale bars, 500 and 200 µm (G); 500 and 50 µm (H) (*n* = 5 slices/3 mice per group). (I–K) Cre‐dependent retrograde tracing with RetroAAV‐DIO‐mCherry injected into the PPC of *Th*‐Cre mice. (I) Schematic of the injection strategy. (J) Injection site in the PPC. (K) mCherry^+^ neurons in the LC co‐labeled with TH. Scale bars, 500 and 200 µm (J); 500 and 50 µm (K) (n = 5 slices/3 mice per group). Significance was assessed by two‐way ANOVA with Bonferroni's post‐hoc test in (B) and (C). Data are presented as mean ± SEM. Exact *p*‐values, degrees of freedom, and pairwise comparisons are provided in the Source Data file. Created with BioRender.com.

We first constructed a protein–protein interaction network by integrating our differentially expressed proteins with curated interaction databases (Figure 7A). NET (SLC6A2), A2AR (ADORA2A), and GNG7 emerged as distinct yet interconnected hubs within a broader network encompassing neurotransmitter transporters, monoamine‐synthesizing enzymes, and G protein‐coupled signaling components. Notably, A2AR and GNG7 exhibited particularly strong interaction edges, suggesting functional coupling within purinergic signaling cascades that may, in turn, modulate NEergic transmission.

These proteomic changes were independently confirmed at both transcript and protein levels. RT‐qPCR analysis revealed significant upregulation of *Slc6a2* (encoding NET) and downregulation of *Adora2a* (encoding A2AR) and *Gng7* in cancer mice relative to sham controls (Figure 7B). Western blot corroborated these findings, showing elevated NET and diminished A2AR and GNG7 protein expression in the PPC of cancer mice (Figure 7C).

To determine the subcellular localization of these key molecules, we performed double immunofluorescence staining with the presynaptic marker synaptophysin. Both NET (Figure 7D) and A2AR (Figure 7E) displayed punctate immunoreactivity that partially co‐localized with synaptophysin in the PPC, indicating their presence at presynaptic terminals where they are positioned to directly regulate neurotransmitter release and reuptake. In particular, NET exhibited robust co‐localization with synaptophysin at presumptive NEergic varicosities, consistent with its canonical role in synaptic NE clearance [48].

The predominant presynaptic localization of NET in the PPC raised a critical question: from which brain region do these NEergic terminals originate? Given that the LC constitutes the principal source of cortical NEergic innervation [49], we employed complementary neuroanatomical tracing strategies to test for a direct LC→PPC projection. First, retrograde tracer CTB‐488 was injected into the PPC of C57 mice (Figure 7F). Examination of injection sites confirmed tracer deposition restricted to the PPC (Figure 7G), and retrogradely labeled somata were identified in the LC, where they co‐expressed tyrosine hydroxylase (TH), the rate‐limiting enzyme for catecholamine synthesis and a definitive marker of NEergic neurons (Figure 7H). To achieve cell‐type specificity, we next injected RetroAAV‐DIO‐mCherry into the PPC of *Th*‐Cre mice (Figure 7I). Consistent with the CTB results, mCherry‐expressing cell bodies were detected at the PPC injection site (Figure 7J) and, critically, within TH‐positive LC neurons (Figure 7K), demonstrating a direct NEergic projection from the LC→PPC.

Taken together, these experiments validate the proteomic candidates at the mRNA, protein, and subcellular levels, and identify the LC→PPC circuit as the anatomical substrate through which NET upregulation may drive excessive NE clearance and diminished NEergic tone, thereby linking synaptic protein dysregulation to the neurotransmitter signaling deficits observed in BCP‐CDS comorbidity.

### 2.6 Astrocyte‐Derived ATP/Ado Activates Presynaptic A2AR to Drive NE Release in PPC

Having identified dysregulation of A2AR, NET, and GNG7 in the PPC and confirmed LC‐derived NEergic innervation to this region, we next investigated whether the astrocytic purinergic cascade functionally regulates NEergic transmission and contributes to comorbid phenotypes.

**FIGURE 8:**
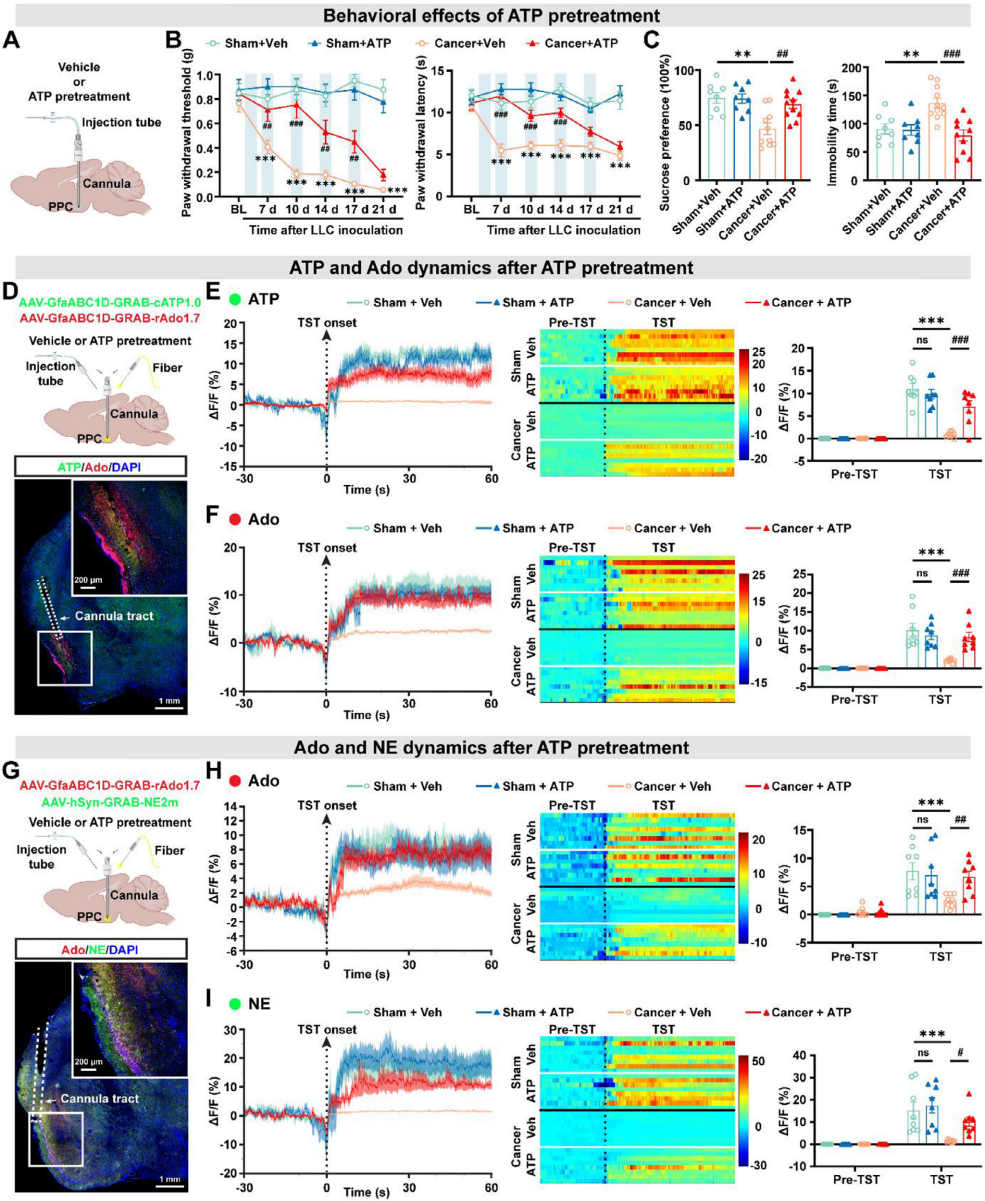
ATP pretreatment in the PPC alleviates comorbid behaviors and restores purinergic and NEergic signaling in BCP‐CDS mice. (A) Schematic of intra‐PPC cannula implantation for vehicle or ATP pretreatment. (B) Time course of PWT (left) and paw withdrawal latency (right) following LLC inoculation. BL, baseline (*n* = 8–12 mice per group; ^**^ *p* < 0.01, ^***^ *p* < 0.001 for sham + Veh vs. cancer + Veh; ^##^ *p* < 0.01, ^###^ *p* < 0.001 for cancer + Veh vs. cancer + ATP). (C) Sucrose preference (left) and immobility time in TST (right) across four groups (*n* = 8–11 mice per group; ^**^ *p* < 0.01 for sham + Veh vs. cancer + Veh; ^##^ *p* < 0.01, ^###^ *p* < 0.001 for cancer + Veh vs. cancer + ATP). (D) Schematic of dual‐sensor fiber photometry strategy. AAV‐GfaABC1D‐GRAB‐cATP1.0 and AAV‐GfaABC1D‐GRAB‐rAdo1.7 were co‐injected with a cannula and fiber implanted into the PPC. Bottom, representative confocal image confirming sensor co‐expression and cannula placement. Scale bars, 200 µm (inset) and 1 mm. (E) Astrocytic ATP dynamics during TST. Left, average ΔF/F (%) traces aligned to TST onset. Middle, heatmaps of individual trials. Right, quantification of mean ΔF/F during Pre‐TST and TST epochs (*n* = 8 mice per group; ^***^ *p* < 0.001 for sham + Veh vs. cancer + Veh; ^###^ *p* < 0.001 for cancer + Veh vs. cancer + ATP). (F) Astrocytic Ado dynamics recorded simultaneously with ATP in the same cohort as (E). Displayed as in (E) (*n* = 8 mice per group; ^***^ *p* < 0.001 for sham + Veh vs. cancer + Veh; ^###^ *p* < 0.001 for cancer + Veh vs. cancer + ATP). (G) Schematic of the second cohort. AAV‐GfaABC1D‐GRAB‐rAdo1.7 and AAV‐hSyn‐GRAB‐NE2m were co‐injected with a cannula and fiber into the PPC. Bottom, representative confocal image. Scale bars, 200 µm (inset) and 1 mm. (H) Astrocytic Ado dynamics during TST after ATP pretreatment in the second cohort. Displayed as in (E) (*n* = 8 mice per group; ^***^ *p* < 0.001 for sham + Veh vs. cancer + Veh; ^##^ *p* < 0.01 for cancer + Veh vs. cancer + ATP). (I) Neuronal NE dynamics recorded simultaneously with Ado in the same cohort as (H). Displayed as in (E) (*n* = 8 mice per group; ^***^ *p* < 0.001 for sham + Veh vs. cancer + Veh; ^#^ *p* < 0.05 for cancer + Veh vs. cancer + ATP). Significance was assessed using two‐way ANOVA with Bonferroni's post‐hoc test in (B), (E), (F), (H), and (I), and one‐way ANOVA with Bonferroni's post‐hoc test in (C). Data are presented as mean ± SEM. Exact *p*‐values, degrees of freedom, and pairwise comparisons are provided in the Source Data file. Created with BioRender.com.

To determine whether restoring astrocytic ATP signaling could rescue NEergic transmission and alleviate comorbid behaviors, we performed repeated intra‐PPC ATP infusions in cancer mice (Figure 8A). ATP treatment significantly attenuated mechanical allodynia, thermal hyperalgesia, anhedonia, and behavioral despair (Figure 8B,C). Fiber photometry using co‐expressed astrocytic GRAB‐cATP1.0 and GRAB‐rAdo1.7 sensors (Figure 8D) revealed that ATP and Ado signals during TST were markedly blunted in vehicle‐treated cancer mice but were restored by ATP pretreatment (Figure 8E,F). In a parallel cohort co‐expressing GRAB‐rAdo1.7 and neuronal GRAB‐NE2m (Figure 8G), ATP pretreatment similarly rescued TST‐evoked Ado and NE transients (Figure 8H,I), supporting a functional ATP→Ado→NE signaling link. This cascade also operated under nociceptive conditions, as ATP pretreatment augmented von Frey‐evoked ATP, Ado, and NE transients in cancer mice (Figure S7A–C).

**FIGURE 9:**
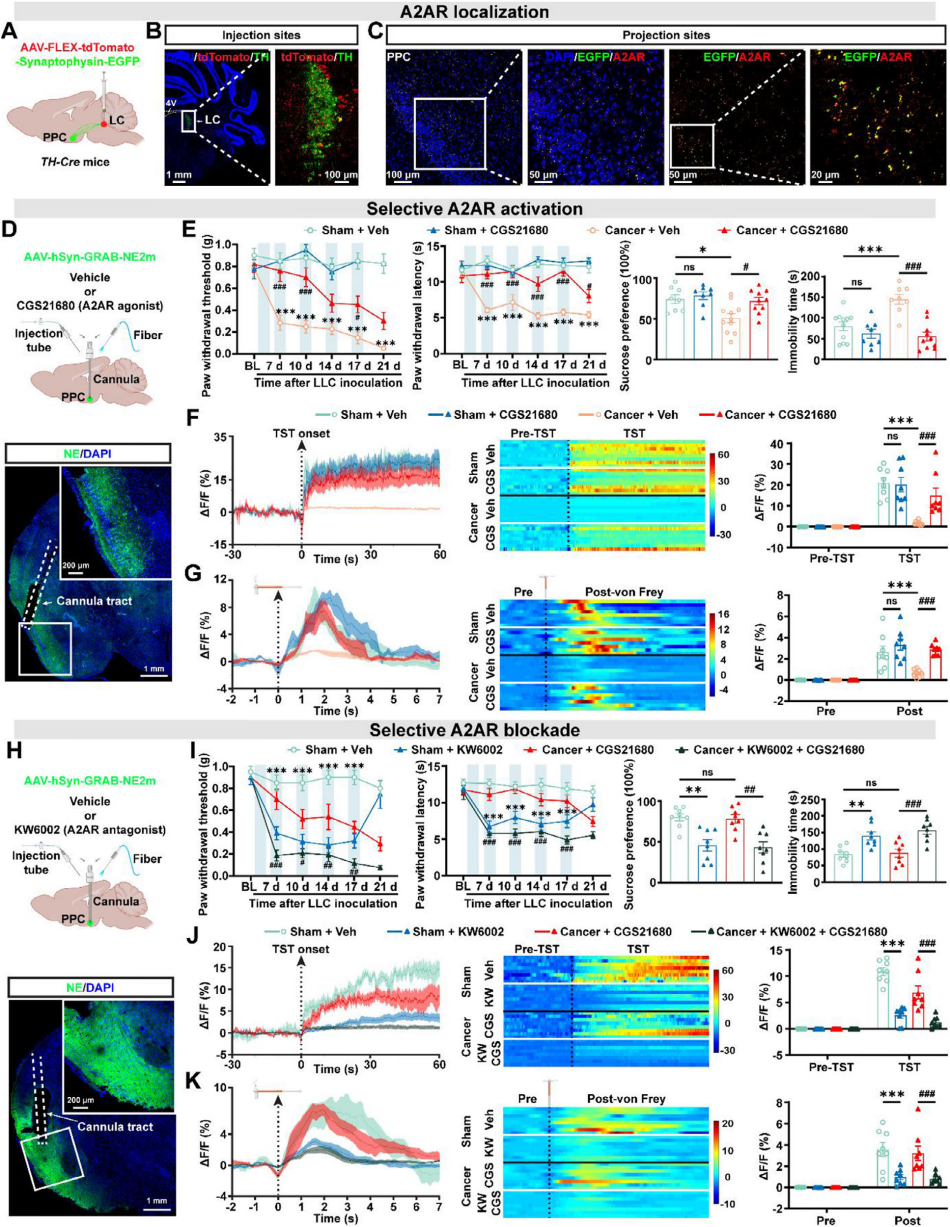
Presynaptic A2AR on LC→PPC NEergic terminals bidirectionally regulates NE release and pain‐ and depression‐like behaviors. (A) Schematic of Cre‐dependent anterograde tracing. AAV‐FLEX‐tdTomato‐Synaptophysin‐EGFP was injected into the LC of *Th*‐Cre mice. (B) Representative images of the LC injection site showing tdTomato and TH co‐labeling. 4 V, fourth ventricle. Scale bars, 1 mm and 100 µm. (C) Synaptophysin‐EGFP^+^ presynaptic terminals co‐localize with A2AR immunostaining in the PPC. Arrowheads indicate co‐localized puncta. Scale bars, 100, 50, and 20 µm. (D) Schematic (top) and representative confocal image (bottom) of intra‐PPC NE sensor (AAV‐hSyn‐GRAB‐NE2m) combined with a cannula for drug delivery and fiber photometry recording. Dashed lines indicate the cannula tract. Scale bars, 200 µm (inset) and 1 mm. (E) PWT (far left), PWL (middle left), sucrose preference (middle right), and immobility time in TST (far right) across sham + Veh, sham + CGS21680, cancer + Veh, and cancer + CGS21680 groups (*n* = 8–12 mice per group). (F) NE dynamics during TST. Left, average ΔF/F (%) traces aligned to TST onset. Middle, heatmaps of individual trials. Right, mean ΔF/F during Pre‐TST and TST epochs (*n* = 8 mice per group). (G) NE dynamics during von Frey stimulation. Displayed as in (F) (*n* = 8 mice per group). (H) Schematic (top) and confocal image (bottom) of the A2AR blockade experiment. KW6002 (A2AR antagonist) was co‐administered with CGS21680 via intra‐PPC cannula. Scale bars, 200 µm (inset) and 1 mm. (I) PWT (far left), PWL (middle left), sucrose preference (middle right), and immobility time in TST (far right) across sham + Veh, sham + KW6002, cancer + CGS21680, and cancer + KW6002 + CGS21680 groups (*n* = 8 mice per group). (J) NE dynamics during TST. Displayed as in (F) (*n* = 8 mice per group). (K) NE dynamics during von Frey stimulation. Displayed as in (F) (*n* = 8 mice per group). In (E)–(G): ^*^ *p* < 0.05, ^***^ *p* < 0.001 for sham + Veh vs. cancer + Veh; ^#^ *p* < 0.05, ^###^ *p* < 0.001 for cancer + Veh vs. cancer + CGS21680. In (I)–(K): ^**^ *p* < 0.01, ^***^ *p* < 0.001 for sham + Veh vs. cancer + KW6002 + CGS21680; ^#^ *p* < 0.05, ^##^ *p* < 0.01, ^###^ *p* < 0.001 for cancer + CGS21680 vs. cancer + KW6002 + CGS21680. Significance was assessed using two‐way ANOVA with Bonferroni's post‐hoc test for PWT and PWL in (E) and (I), and for (F), (G), (J), and (K); one‐way ANOVA with Bonferroni's post‐hoc test for SPT and TST in (E) and (I). Data are presented as mean ± SEM. Exact *p*‐values, degrees of freedom, and pairwise comparisons are provided in the Source Data file. Created with BioRender.com.

To identify the receptor transducing the Ado signal, we injected Cre‐dependent AAV‐FLEX‐tdTomato and AAV‐FLEX‐Synaptophysin‐EGFP into the LC of *Th*‐Cre mice to label NEergic presynaptic terminals (Figure 9A,B). In the PPC, EGFP‐labeled presynaptic puncta exhibited robust co‐localization with A2AR immunofluorescence (Figure 9C), placing A2AR precisely at LC→PPC NEergic terminals.

We next tested whether selective A2AR activation is sufficient to rescue NEergic signaling and comorbid phenotypes. Intra‐PPC infusion of the selective A2AR agonist CGS21680 (Figure 9D) alleviated pain and depression‐like behaviors (Figure 9E) and restored NE transients during both TST and von Frey stimulation (Figure 9F,G). Conversely, co‐administration of the selective A2AR antagonist KW6002 with CGS21680 (Figure 9H) completely abolished all behavioral improvements (Figure 9I) and eliminated restored NE dynamics (Figure 9J,K), establishing A2AR as both necessary and sufficient for Ado‐mediated NE release. The non‐selective adenosine receptor agonist NECA similarly alleviated comorbid behaviors and restored NE dynamics (Figure S8A–D), further supporting Ado‐mediated modulation of NEergic tone; however, the complete blockade by KW6002 suggests A2AR as the principal mediator.

We further examined whether A2AR activation could reverse the molecular signatures of NEergic dysfunction. In cancer mice, chronic CGS21680 treatment significantly upregulated *Adora2a* and *Gng7* mRNA while downregulating *Slc6a2* mRNA (Figure S8E). Accordingly, NET protein levels were also significantly reduced following CGS21680 treatment (Figure S8F), collectively reversing the proteomic alterations identified in the PPC. These results indicate that A2AR activation coordinately restores purinergic signaling components and suppresses NET expression at both transcriptional and translational levels, thereby reducing NE reuptake capacity and prolonging synaptic NE availability.

These experiments delineate a multi‐step astrocyte‐to‐neuron signaling cascade in the PPC, proceeding from astrocytic ATP through Ado and presynaptic A2AR to NE release, that is disrupted in BCP‐depression comorbidity. Pharmacological restoration at any node rescues NEergic transmission and alleviates both pain and depression‐like behaviors, while A2AR blockade abolishes these effects. Integrated with the A2‐to‐A1 astrocytic phenotype transition and metabolic reprogramming (Figures 3 and 4), these findings identify impaired astrocytic ATP output and consequent A2AR hypoactivation as upstream drivers of NEergic deficiency linking nociceptive hypersensitivity to affective disturbance, highlighting the therapeutic potential of targeting this purinergic–NEergic axis.

### 2.7 NEergic Signaling in the PPC Bidirectionally Modulates Sensory and Affective Deficits in Cancer Pain–Depression Comorbidity

**FIGURE 10:**
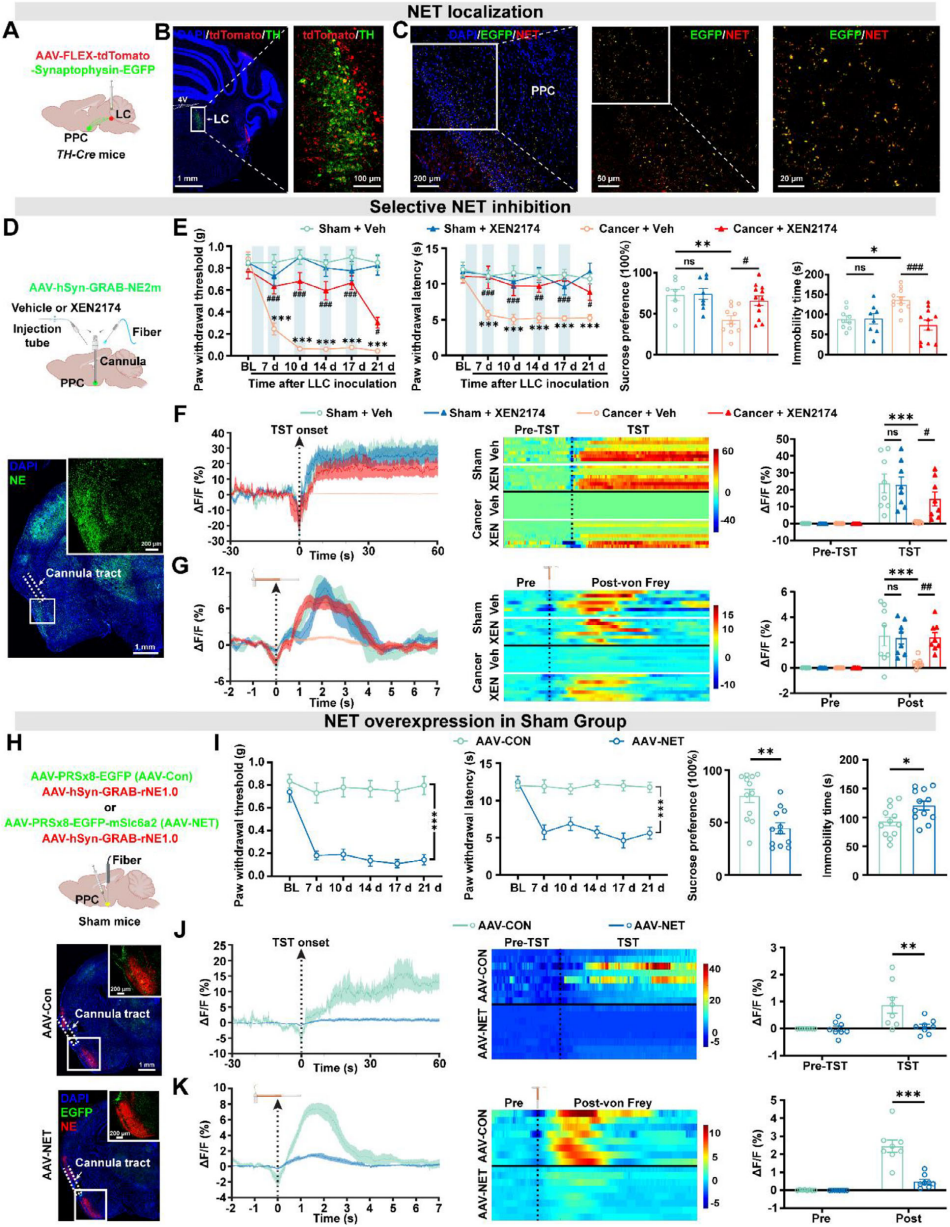
NET on LC→PPC NEergic terminals controls synaptic NE availability and comorbid pain–depression phenotypes. (A) Schematic of Cre‐dependent anterograde tracing. AAV‐FLEX‐tdTomato‐Synaptophysin‐EGFP was injected into the LC of *Th‐*Cre mice. (B) Representative images of the LC injection site showing tdTomato and TH co‐labeling. 4 V, fourth ventricle. Scale bars, 1 mm and 100 µm. (C) Synaptophysin‐EGFP^+^ presynaptic terminals co‐localize with NET immunostaining in the PPC. Arrowheads indicate co‐localized puncta. Scale bars, 200, 50, and 20 µm. (*n* = 5 slices/3 mice per group) (D) Schematic (top) and representative confocal image (bottom) of intra‐PPC NE sensor (AAV‐hSyn‐GRAB‐NE2m) combined with cannula for drug delivery and fiber photometry recording. Vehicle or XEN2174 (selective NET inhibitor) was delivered via injection tube. Dashed lines indicate the cannula tract. Scale bars, 200 µm (inset) and 1 mm. (E) PWT (far left), PWL (middle left), sucrose preference (middle right), and immobility time in TST (far right) across sham + Veh, sham + XEN2174, cancer + Veh, and cancer + XEN2174 groups (*n* = 8–12 mice per group). (F) NE dynamics during TST. Left, average ΔF/F (%) traces aligned to TST onset. Middle, heatmaps of individual trials. Right, mean ΔF/F during Pre‐TST and TST epochs (*n* = 8 mice per group). (G) NE dynamics during von Frey stimulation. Displayed as in (F) (*n* = 8 mice per group). (H) Schematic (top) and representative confocal images (bottom) of NET overexpression in sham mice. AAV‐PRSx8‐EGFP (AAV‐Con) or AAV‐PRSx8‐EGFP‐mSlc6a2 (AAV‐NET) was co‐injected with AAV‐hSyn‐GRAB‐rNE1.0 into the PPC, combined with fiber implantation. Top: AAV‐Con; bottom: AAV‐NET showing DAPI, EGFP, and NET immunostaining. Dashed lines indicate fiber tract. Scale bars, 200 µm (inset) and 1 mm. (I) PWT (far left), PWL (middle left), sucrose preference (middle right), and immobility time in TST (far right) in AAV‐Con and AAV‐NET sham groups (*n* = 10‐12 mice per group). (J) NE dynamics during TST. Displayed as in (F) (*n* = 8 mice per group). (K) NE dynamics during von Frey stimulation. Displayed as in (F) (*n* = 8 mice per group). In (E)–(G): ^*^ *p* < 0.05, ^**^ *p* < 0.01, ^***^ *p* < 0.001 for sham + Veh vs. cancer + Veh; ^#^ *p* < 0.05, ^##^ *p* < 0.01, ^###^ *p* < 0.001 for cancer + Veh vs. cancer + XEN2174. In (I)–(K): ^*^ *p* < 0.05, ^**^ *p* < 0.01, ^***^ *p* < 0.001 for AAV‐Con vs. AAV‐NET. Significance was assessed using two‐way ANOVA with Bonferroni's post‐hoc test for PWT and PWL in (E) and (I), and for (F), (G), (J), and (K); one‐way ANOVA with Bonferroni's post‐hoc test for SPT and TST in (E); unpaired two‐tailed Student's *t*‐test for SPT and TST in (I). Data are presented as mean ± SEM. Exact *p*‐values, degrees of freedom, and pairwise comparisons are provided in the Source Data file. Created with BioRender.com.

Building on the identified ATP/Ado/A2AR/NE signaling pathway and proteomic evidence of NET upregulation, we investigated the causal role of NEergic transmission in cancer pain–depression comorbidity. Using anterograde viral tracing in *Th*‐Cre mice, we first confirmed robust NET expression on NEergic terminals projecting from the LC to PPC (Figure 10A–C).

The selective NET inhibitor XEN2174 was then administered into the PPC of cancer mice while NE dynamics were monitored via fiber photometry (Figure 10D). XEN2174 alleviated mechanical and thermal hypersensitivity, anhedonia, and behavioral despair (Figure 10E), accompanied by significantly augmented NE fluorescence signals during both TST and noxious mechanical stimulation (Figure 10F,G). Conversely, viral overexpression of NET in the PPC of sham mice (Figure 10H), mechanical and thermal hypersensitivity, decreased sucrose preference, and increased immobility (Figure 10I), with markedly attenuated stimulus‐evoked NE signals during TST and von Frey stimulation (Figure 10J,K), recapitulating the comorbid phenotypes of cancer mice.

**FIGURE 11:**
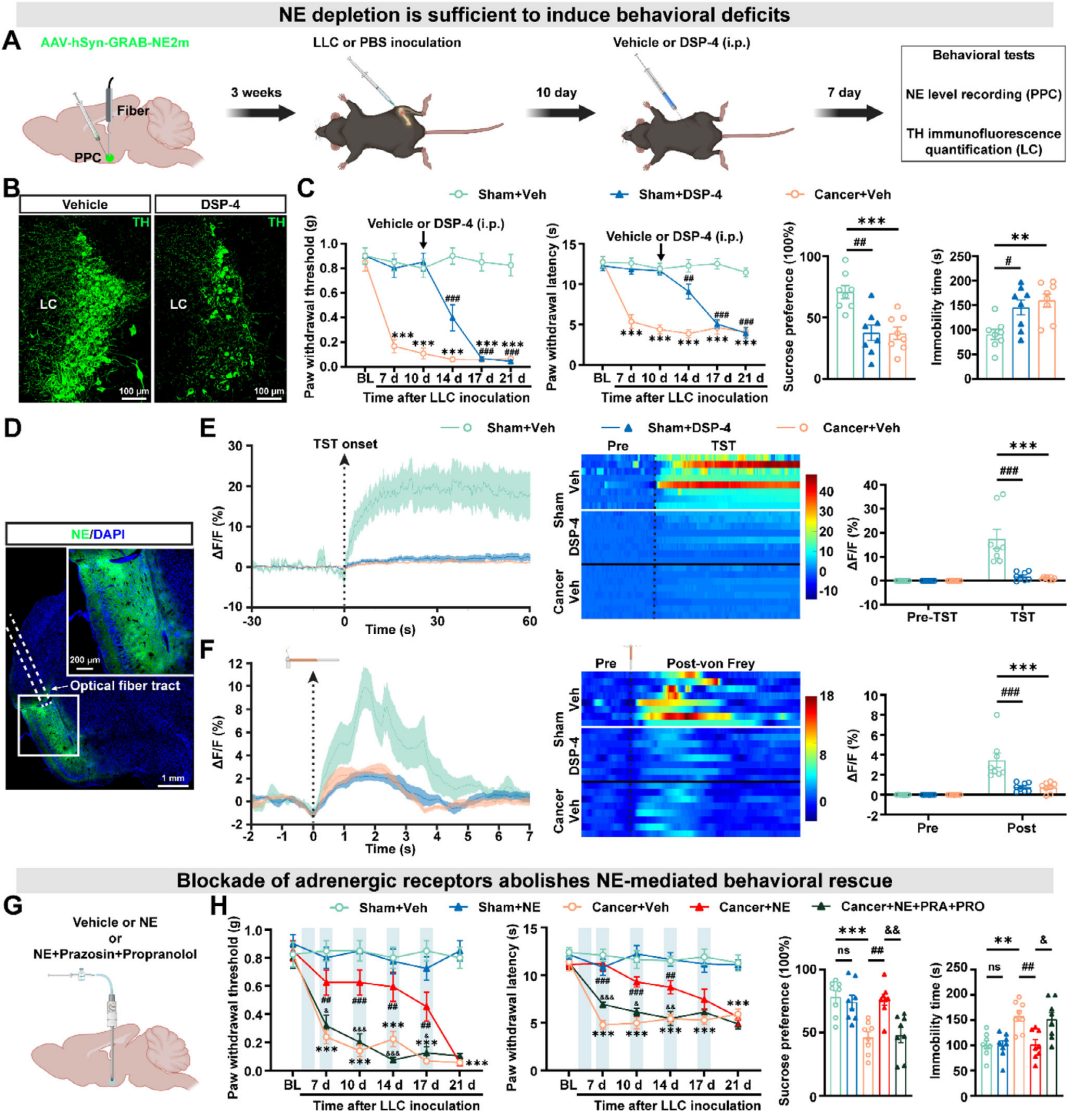
Bidirectional NEergic control of pain and depressive‐like behaviors. (A) Experimental timeline. AAV‐hSyn‐GRAB‐NE2m was injected into the PPC with optical fiber implantation. After 3 weeks of viral expression, mice received LLC or PBS (sham) inoculation. Vehicle or DSP‐4 was administered intraperitoneally (i.p.) at 10 days post‐inoculation. Behavioral tests, fiber photometry recording of NE levels in the PPC, and TH immunofluorescence quantification in the LC were performed 7 days later. (B) Representative TH immunofluorescence images in the LC from Vehicle‐ and DSP‐4‐treated mice, confirming NEergic neuron depletion by DSP‐4. Scale bar, 100 µm. (C) PWT (far left), PWL (middle left), sucrose preference (middle right), and immobility time in TST (far right) for sham + Veh, sham + DSP‐4, and cancer + Veh groups. Arrows indicate Vehicle or DSP‐4 administration. DSP‐4 treatment in sham mice recapitulated pain hypersensitivity and depressive‐like behaviors comparable to those in cancer mice (*n* = 8 mice per group). (D) Representative images showing AAV‐hSyn‐GRAB‐NE2m expression (green) and DAPI (blue) in the PPC (top), and the fiber tract at low magnification (bottom). Scale bars, 200 µm (top) and 1 mm (bottom). (E) NE dynamics in the PPC during TST. Left, average ΔF/F (%) traces aligned to TST onset (dashed line); shaded areas represent SEM. Middle, heatmaps of individual NE responses during Pre‐TST and TST periods. Right, quantification of mean ΔF/F (%) during Pre‐TST and TST epochs (*n* = 8 mice per group). (F) NE dynamics in the PPC during von Frey stimulation. Left, average ΔF/F (%) traces aligned to mechanical stimulation onset. Middle, heatmaps of individual NE responses during Pre‐ and Post‐von Frey periods. Right, quantification of mean ΔF/F (%) during Pre‐ and Post‐von Frey epochs (*n* = 8 mice per group). (G) Schematic of intra‐PPC administration of Vehicle, NE, or NE combined with the α_1_‐adrenergic receptor antagonist prazosin (PRA) and the β‐adrenergic receptor antagonist propranolol (PRO). (H) PWT (far left), PWL (middle left), sucrose preference (middle right), and immobility time in TST (far right) for sham + Veh, sham + NE, cancer + Veh, cancer + NE, and cancer + NE + PRA + PRO groups. Intra‐PPC NE rescued pain and depressive‐like behaviors in cancer mice, an effect that was abolished by co‐administration of adrenergic receptor antagonists (*n* = 8 mice per group). In (C), (E), and (F): ^**^ *p* < 0.01, ^***^ *p* < 0.001 for sham + Veh vs. cancer + Veh; ^#^ *p* < 0.05, ^##^ *p* < 0.01, ^###^ *p* < 0.001 for sham + Veh vs. sham + DSP‐4. In (H): ^**^ *p* < 0.01, ^***^ *p* < 0.001 for sham + Veh vs. cancer + Veh; ^##^ *p* < 0.01, ^###^ *p* < 0.001 for cancer + Veh vs. cancer + NE; ^&^ *p* < 0.05, ^&&^ *p* < 0.01, ^&&&^ *p* < 0.001 for cancer + NE vs. cancer + NE + PRA + PRO; ns, not significant. Significance was assessed using two‐way ANOVA with Bonferroni's post‐hoc test for PWT and PWL in (C) and (H), and for (E) and (F); one‐way ANOVA with Bonferroni's post‐hoc test for SPT and TST in (C) and (H). Data are presented as mean ± SEM. Exact *p*‐values, degrees of freedom, and pairwise comparisons are provided in the Source Data file. Created with BioRender.com.

To further confirm that NE deficiency itself is sufficient to drive these deficits, sham mice were treated with the NEergic neurotoxin DSP‐4 (Figure 11A), which markedly reduced TH immunoreactivity in the LC (Figure 11B) and induced mechanical and thermal hypersensitivity, anhedonia, and behavioral despair, closely phenocopying cancer mice (Figure 11C). Fiber photometry recordings in the PPC (Figure 11D) further demonstrated that DSP‐4 attenuated NE release during both TST (Figure 11E) and noxious mechanical stimulation (Figure 11F). Conversely, direct intra‐PPC NE supplementation in cancer mice (Figure 11G) significantly improved mechanical and thermal pain thresholds, sucrose preference, and immobility; these rescue effects were completely abolished by co‐administration of the adrenergic antagonists prazosin (α_1_) and propranolol (β) (Figure 11H), confirming that NE modulates both nociceptive and affective processing through adrenergic receptors.

Collectively, these four complementary approaches, namely NET inhibition, NET overexpression, NE depletion, and NE supplementation with receptor blockade, establish NE availability in the PPC as a key determinant of cancer pain–depression comorbidity. When integrated with the ATP/Ado/A2AR axis, these findings delineate a multi‐step signaling cascade: A2‐to‐A1 astrocytic phenotype transition impairs purinergic signaling, reduces A2AR‐mediated regulation of NET, and consequently diminishes synaptic NE levels. Supporting this model, the A2AR agonist CGS21680 significantly upregulated *Gng7* mRNA (Figure S8E), a G‐protein subunit involved in A2AR signaling [50], suggesting that GNG7 may serve as a potential mediator linking A2AR activation to NET regulation. This cascade offers a plausible mechanistic framework in which astrocyte metabolic reprogramming contributes to both sensory hypersensitivity and affective disturbances in cancer pain–depression comorbidity.

### 2.8 Reversal of A1 Astrocyte Phenotype Normalizes PPC Neurochemistry and Alleviates Comorbid Behaviors

The preceding findings demonstrated that BCP‐depression comorbidity drives A2‐to‐A1 astrocyte conversion in the PPC, triggering sequential reductions in ATP, Ado, A2AR signaling, and synaptic NE. This raised a critical question: could reversing this phenotypic shift normalize downstream neurochemistry and rescue comorbid behaviors?

**FIGURE 12:**
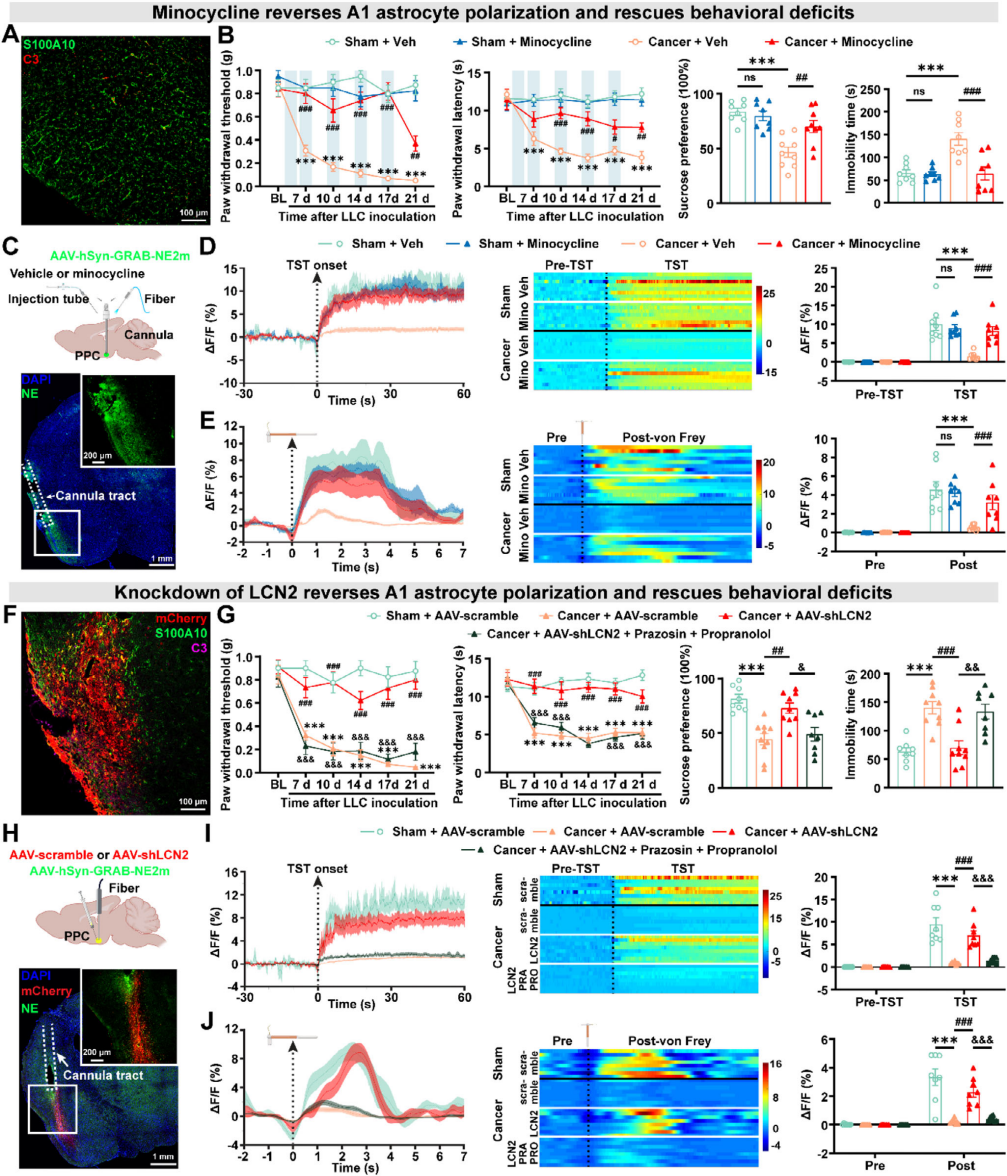
Minocycline treatment and *Lcn2* knockdown restore PPC NE signaling and rescue behavioral deficits. (A) Representative S100A10 and C3 immunofluorescence in the PPC following minocycline treatment. Scale bar, 100 µm. (B) PWT (far left), PWL (middle left), sucrose preference (middle right), and immobility time in TST (far right) across sham + Veh, sham + minocycline, cancer + Veh, and cancer + minocycline groups (*n* = 8–12 mice per group). (C) Schematic (top) of GRAB‐NE2m injection, fiber photometry recording, and vehicle/minocycline delivery in the PPC. Representative images (bottom) of GRAB‐NE2m expression and cannula tract. Scale bars, 200 µm (inset) and 1 mm. (D) NE dynamics in the PPC during TST. Left, average ΔF/F (%) traces aligned to TST onset (dashed line); shaded areas represent SEM. Middle: heatmaps of individual trials. Right, quantification of mean ΔF/F (%) during Pre‐TST and TST epochs (*n* = 8 mice per group). (E) NE dynamics in the PPC during von Frey stimulation. Displayed as in (D) (*n* = 8 mice per group). (F) Representative mCherry, S100A10, and C3 immunofluorescence in the PPC following AAV‐shLCN2 injection. Scale bar, 100 µm. (G) PWT (far left), PWL (middle left), sucrose preference (middle right), and immobility time in TST (far right) across sham + AAV‐scramble, cancer + AAV‐scramble, cancer + AAV‐shLCN2, and cancer + AAV‐shLCN2 + Prazosin + Propranolol groups (*n* = 8–10 mice per group). (H) Schematic (top) of AAV‐shLCN2 and GRAB‐NE2m co‐injection with fiber photometry in the PPC. Representative images (bottom) of mCherry and GRAB‐NE2m co‐expression and cannula tract. Scale bars, 200 µm (inset) and 1 mm. (I) NE dynamics during TST across sham + AAV‐scramble, cancer + AAV‐scramble, cancer + AAV‐shLCN2, and cancer + AAV‐shLCN2 + Prazosin + Propranolol groups. Displayed as in (D) (*n* = 8–10 mice per group). (J) NE dynamics during von Frey stimulation across groups as in (I). Displayed as in (D) (*n* = 8–10 mice per group). In (B), (D), and (E): ^***^ *p* < 0.001 for sham + Veh vs. cancer + Veh; ^#^ *p* < 0.05, ^##^ *p* < 0.01, ^###^ *p* < 0.001 for cancer + Veh vs. cancer + Minocycline. In (G), (I), and (J): ^***^ *p* < 0.001 for sham + AAV‐scramble vs. cancer + AAV‐scramble; ^##^ *p* < 0.01, ^###^ *p* < 0.001 for cancer + AAV‐scramble vs. cancer + AAV‐shLCN2; ^&^ *p* < 0.05, ^&&^ *p* < 0.01, ^&&&^ *p* < 0.001 for cancer + AAV‐shLCN2 vs. cancer + AAV‐shLCN2 + Prazosin + Propranolol; ns, not significant. Significance was assessed using two‐way ANOVA with Bonferroni's post‐hoc test for PWT and PWL in (B) and (G), and for (D), (E), (I), and (J); one‐way ANOVA with Bonferroni's post‐hoc test for SPT and TST in (B) and (G). Data are presented as mean ± SEM. Exact *p*‐values, degrees of freedom, and pairwise comparisons are provided in the Source Data file. Created with BioRender.com.

Two complementary strategies were employed to promote A1‐to‐A2 astrocyte conversion in cancer‐bearing mice: (1) systemic minocycline administration, which facilitates astrocyte phenotype transition and possesses analgesic properties [51, 52], and (2) PPC‐targeted *Lcn2* knockdown via AAV‐shLCN2, which attenuates pro‐inflammatory A1 reactivity [53]. Pharmacological suppression of reactive astrogliosis with minocycline shifted PPC astrocyte phenotype toward the A2 state, as evidenced by reduced C3 (A1 marker) expression and preserved S100A10 (A2 marker) staining compared with vehicle‐treated cancer mice (Figure 12A). Behaviorally, minocycline restored mechanical withdrawal thresholds and thermal withdrawal latencies, increased sucrose preference, and reduced TST immobility time in cancer mice, without affecting baseline behaviors in sham controls (Figure 12B). To probe the underlying neurochemical changes, fiber photometry with the neuronal GRAB‐NE2m sensor was performed in the PPC through a combined cannula/fiber assembly (Figure 12C). Minocycline‐treated cancer mice exhibited significantly increased NE signals during the TST (Figure 12D) and upon von Frey stimulation (Figure 12E), approaching levels observed in sham mice. To determine whether the upstream purinergic cascade was also normalized, astrocyte‐targeted GRAB‐cATP1.0 and GRAB‐rAdo1.7 sensors were co‐expressed in PPC astrocytes (Figure S9A). Minocycline treatment significantly elevated astrocytic ATP signals during both the TST and von Frey stimulation in cancer mice (Figure S9B), and similarly restored Ado signals in both paradigms (Figure S9C).

To genetically validate the causal contribution of astrocyte‐derived LCN2, AAV‐shLCN2 was injected into the PPC, which similarly reduced C3 expression and preserved S100A10 staining in mCherry‐labeled astrocytes (Figure 12F). *Lcn2* knockdown produced comparable improvements in pain‐ and depression‐like behaviors (Figure 12G). To determine whether these behavioral benefits required intact NE neurotransmission, a separate cohort of shLCN2‐treated cancer mice received co‐administration of the α_1_‐adrenergic antagonist prazosin and the β‐adrenergic antagonist propranolol; this combined adrenergic blockade completely abolished the analgesic and antidepressant effects of *Lcn2* knockdown (Figure 12G), indicating that the behavioral rescue conferred by A1‐to‐A2 phenotype reversion is dependent on downstream NE signaling. Consistently, fiber photometry with neuronal GRAB‐NE2m (Figure 12H) revealed that shLCN2 normalized stimulus‐evoked NE signals during the TST (Figure 12I) and upon von Frey stimulation (Figure 12J), whereas concurrent prazosin/propranolol treatment significantly attenuated these NE responses, mirroring the behavioral impairment. To examine the upstream purinergic cascade, GRAB‐cATP1.0 was expressed in PPC astrocytes (Figure S10A): shLCN2 restored astrocytic ATP signals during both the TST and von Frey stimulation, while concurrent adrenergic blockade abolished this recovery (Figure S10B). A parallel experiment with GRAB‐Ado1.0m (Figure S10C) demonstrated that shLCN2 likewise normalized astrocytic Ado release in both paradigms, an effect that was again abrogated by prazosin/propranolol co‐treatment (Figure S10D).

In summary, these results demonstrate that reversing the A1 astrocyte phenotype in the PPC, through either pharmacological (minocycline) or genetic (*Lcn2* knockdown) approaches, normalizes the ATP → Ado → A2AR → NET → NE signaling cascade and alleviates comorbid pain hypersensitivity and depression‐like behaviors. The finding that adrenergic receptor blockade abolishes the behavioral benefits of phenotype reversion confirms NE signaling as the critical downstream effector linking astrocyte phenotypic state to behavioral outcomes.

## 3 Discussion

This study reveals a previously uncharacterized astrocyte‐neuron signaling axis in the PPC that coordinately drives CDS in BCP. We demonstrate that BCP‐induced A2‐to‐A1 astrocyte conversion triggers cascading dysregulation of purinergic (ATP/Ado/A2AR) and NEergic (NET/NE) signaling, collectively driving nociceptive hypersensitivity and affective disturbances. Interventions at discrete nodes of this axis, including ATP supplementation, A2AR agonism, NET inhibition, NE supplementation, minocycline administration, and *Lcn2* knockdown, all restored PPC neurochemical homeostasis and alleviated comorbid behaviors, whereas disruption of individual nodes in healthy mice was sufficient to recapitulate the pain–depression comorbidity phenotype. Together, these findings establish that cortical astrocyte phenotype conversion regulates cancer pain‐depression comorbidity through a defined purinergic‐NEergic cascade, providing a mechanistic framework for multi‐target therapeutic intervention.

The identification of the PPC as a supraspinal hub for pain–depression integration extends the understanding of classically studied structures, including the amygdala [54], hippocampus [55], anterior cingulate cortex [56], and periaqueductal gray [57]. The piriform cortex has traditionally been associated with olfactory processing and implicated in epilepsy, Alzheimer's disease, and autism [13], yet recent studies have linked it to hyperalgesia [58] and antidepressant‐like responses [59]. To systematically identify the most engaged supraspinal node in pain‐depression comorbidity, we employed fMOST whole‐brain imaging to quantify c‐Fos expression across 13 cortical and subcortical regions. Although multiple regions, including the SS, AI, BLA, ZI, and hippocampal CA fields, showed elevated c‐Fos expression in cancer versus sham groups, the PPC exhibited the highest fold change among all regions examined, a result independently validated by immunofluorescence quantification. To resolve the cellular identity underlying this activation signal, we applied an E‐SARE‐based activity‐dependent labeling strategy, which revealed that GFAP‐positive astrocytes constituted the predominant activated cell population in the PPC, significantly exceeding NeuN‐positive neurons and Iba‐1‐positive microglia, thereby positioning astrocyte dysfunction rather than neuronal hyperexcitability at the intersection of sensory and affective processing. A pronounced A2‐to‐A1 conversion, characterized by the transition from S100A10^+^ to C3^+^ astrocytes, was evident by day 21. Although similar shifts have been reported in chronic post‐surgical pain [51], most BCP studies have focused on spinal mechanisms without distinguishing astrocyte subtypes [60]. By localizing this conversion to a cortical site at the convergence of nociceptive and affective circuits, our findings position the PPC A2‐to‐A1 transition as a molecular switch that transforms acute nociception into chronic pain–depression comorbidity.

How bone cancer initiates this cortical astrocyte conversion is an important mechanistic question. We propose that sustained nociceptive afferent input constitutes the primary trigger: tumor growth generates a persistent peripheral inflammatory microenvironment that drives sustained nociceptive bombardment of the PPC via ascending pathways, likely activates resident microglia to release the classical A1‐inducing factors TNF‐α, IL‐1α, and C1q [44]. A complementary humoral route, whereby circulating tumor‐derived inflammatory mediators cross the blood‐brain barrier to act directly on PPC astrocytes, is also plausible. Consistent with this neuroinflammatory hypothesis, AAV‐mediated *Lcn2* knockdown effectively reversed A1 polarization of PPC astrocytes and restored downstream purinergic–NEergic signaling, suggesting that LCN2, as a known neuroinflammatory amplifier [61, 62], plays a critical role in maintaining the A1 phenotype. An alternative explanation, that A1 conversion is a downstream consequence of the depressive state rather than a primary event, deserves consideration but is not supported by our data: nociceptive hypersensitivity preceded depression‐like behaviors, indicating that astrocyte changes are temporally aligned with the nociceptive phase; targeted A1 reversal via minocycline or *Lcn2* knockdown restored the entire downstream cascade and alleviated both sensory and affective behaviors; and NET overexpression in healthy mice, which reproduced the neurochemical consequences without involving astrocyte pathology, was sufficient to induce the comorbid phenotype. Nevertheless, we cannot exclude bidirectional reinforcement at later disease stages, whereby stress‐related neuroendocrine changes may further exacerbate astrocyte dysfunction; dissecting this positive‐feedback interaction through temporally precise astrocyte manipulation remains an important future direction.

Regardless of the precise initiating mechanism, A1 conversion was accompanied by profound metabolic reprogramming. Untargeted metabolomics revealed simultaneous downregulation of ATP, Ado, and NE, and network analysis identified these molecules as coordinately linked hub nodes within the purinergic‐NEergic axis. In vivo fiber photometry using GRAB sensors confirmed these deficits, as ATP, Ado, and NE transient signals were severely attenuated during the TST and mechanical stimulation. Multiple control analyses helped mitigate concerns about technical confounders: sensor expression levels did not differ between groups, and sensors exhibited the expected neuron‐specific localization. As an orthogonal validation, LC‐MS/MS quantification of PPC tissue independently confirmed significant reductions in ATP and Ado content. Notably, this signaling impairment was not task‐specific. Transient amplitudes of all three signals were similarly reduced during home‐cage spontaneous behaviors and the accelerating rotarod challenge. We interpret this cross‐behavioral pattern as a tonic functional deficit in the PPC purinergic–NEergic pathway, providing a common neurochemical substrate for the co‐occurrence of nociceptive hypersensitivity and depression‐like behaviors. Alternative interpretations related to sensor reporting under chronic disease conditions are considered in the limitations section below. Pearson correlation analysis further supported this inference: the degree of A1 polarization was strongly correlated with behavioral indices across domains (|*r*| = 0.69–0.92), and ATP, Ado, and NE levels were each moderately to strongly correlated with most behavioral measures (majority |*r*| > 0.50), indicating a quantitative association between the degree of molecular deficit and the severity of cross‐domain behavioral impairment. These findings are consistent with evidence linking astrocyte‐derived ATP to mood regulation [25, 29], although a report that chronic orofacial pain elevates ventral hippocampal ATP/Ado [24] highlights the region‐specificity of purinergic adaptations. Proteomic analysis further identified the molecular substrate: NET was upregulated while A2AR and its signaling partner GNG7 were downregulated, providing a direct molecular link between astrocyte phenotype conversion and neurotransmitter dysfunction.

We next characterized the functional architecture of this cascade at LC‐PPC terminals. Perfusion of exogenous ATP into the PPC of comorbid mice restored Ado and NE transient signals during behavioral challenges, establishing a sequential ATP‐to‐Ado‐to‐NE signaling hierarchy. Anterograde tracing in *Th*‐Cre mice localized A2AR to NEergic presynaptic terminals, identifying the receptor through which Ado modulates NE release. The selective A2AR agonist CGS21680 reproduced the NE‐enhancing and behavioral effects of ATP supplementation, while the antagonist KW6002 abolished these benefits, demonstrating that A2AR activation is necessary for, and on its own sufficient to reproduce, Ado‐mediated NE facilitation. The non‐selective agonist NECA produced similar effects, but the complete blockade by KW6002 established A2AR as the principal mediating receptor. These findings contrast with reports in which A2AR activity promotes depressive phenotypes in other contexts [34, 54], a discrepancy that likely reflects differences in cellular localization: A2AR on striatal GABAergic neurons inhibits reward signaling, whereas presynaptic A2AR on LC‐PPC NEergic terminals facilitates NE release. This context‐dependence is consistent with observations that A2AR attenuates neuropathic pain across different circuits [38], further confirming that A2AR function is determined by its cellular and regional expression pattern rather than possessing a uniform positive or negative valence.

With A2AR established as the signal‐transducing receptor, we next examined the NE clearance component of the cascade. Anterograde tracing confirmed that LC‐PPC terminals express NET, and its causal role was established through three independent strategies: pharmacological inhibition (XEN2174) enhanced NE signaling and alleviated comorbid behaviors, viral overexpression in healthy mice suppressed NE signaling and induced the comorbid phenotype, and DSP‐4 chemical ablation of LC NEergic neurons independently reproduced these effects from the perspective of neuronal lesion, collectively demonstrating that PPC NEergic insufficiency is sufficient to drive the comorbid phenotype. CGS21680 coordinately upregulated *Adora2a* and *Gng7* mRNA levels while downregulating *Slc6a2* mRNA and protein levels, suggesting tonic inhibitory regulation of NET by A2AR. We speculate that this regulation may operate through the Golf complex (αolf/β2/γ7), in which GNG7 is critical for complex assembly and effector coupling [50, 63, 64], although this remains to be directly verified. The downregulation of GNG7 in comorbid mice would impair Golf‐dependent signal transduction, releasing NET from tonic inhibition and accelerating NE clearance, consistent with reports that GNG7 generates region‐specific transcripts influencing neuronal morphology and behavior [64, 65]. These data indicate that impaired A2AR signaling drives NET upregulation and NEergic insufficiency, aligning with evidence implicating NEergic dysfunction in pain‐depression comorbidity [66] and identifying the A2AR‐NET axis as a tractable therapeutic target. To further validate the downstream effector mechanism of NE, we supplemented exogenous NE in comorbid mice, which significantly restored nociceptive hypersensitivity and depression‐like behaviors; combined application of the α_1_ receptor antagonist prazosin and the β receptor antagonist propranolol completely abolished the therapeutic effects of NE, establishing adrenergic receptor activation as the requisite downstream effector mechanism for NE‐mediated behavioral rescue.

Having delineated the downstream cascade, we tested whether reversing upstream A1 conversion could restore signaling throughout the entire axis. Both minocycline and AAV‐mediated *Lcn2* knockdown shifted PPC astrocytes from a C3‐dominant A1 phenotype toward an S100A10‐dominant A2 phenotype, concurrently alleviating nociceptive hypersensitivity and depression‐like behaviors. Fiber photometry recordings confirmed that phenotype normalization restored stimulus‐evoked ATP, Ado, and NE transient signals; in particular, *Lcn2* knockdown significantly restored NE transient signals in the PPC of comorbid mice during the TST and mechanical stimulation, with effects comparable to those achieved by pharmacological intervention at individual nodes. Crucially, the combined application of prazosin and propranolol in *Lcn2* knockdown comorbid mice completely blocked the behavioral rescue effects and suppressed the functional recovery of NE transient signals, demonstrating that the therapeutic benefits of upstream astrocyte correction ultimately converge on adrenergic receptor activation as the common terminal effector. These results extend previous findings that minocycline attenuates pain behaviors [67] and that phenotype conversion influences neuroinflammatory outcomes [68], establishing a complete causal chain from astrocyte A1/A2 phenotype through purinergic signaling to NEergic transmission and ultimately to adrenergic receptor activation. This indicates that upstream astrocyte‐targeted therapy can propagate corrective effects throughout the entire purinergic‐NEergic axis, providing a tractable single‐node therapeutic entry point that addresses both the sensory and affective dimensions of cancer pain‐depression comorbidity.

Several limitations of this study should be noted. The proposed role of GNG7/Golf in coupling A2AR to NET remains hypothetical and requires validation through conditional gene knockout or phosphorylation state analyses. Our A1/A2 classification may underestimate astrocyte transcriptomic heterogeneity; single‐cell sequencing and spatial transcriptomics would better capture subpopulation diversity [69]. Although fiber photometry offers excellent temporal resolution, two‐photon imaging would better resolve subcellular dynamics and individual astrocyte responses. All experiments used male mice with a single LLC cell line, yet sex differences in pain, depression, and glial biology necessitate broader evaluation. Finally, stereotactic injection poses translational barriers, underscoring the need for blood‐brain barrier‐penetrant delivery systems to achieve noninvasive PPC targeting.

An additional point worth elaborating is the temporal profile of intra‐PPC NE supplementation. NE produced robust behavioral rescue between days 7 and 17, but its effect on nociceptive thresholds (PWT and PWL) was attenuated by day 21, approaching cancer+Veh levels. Notably, this decline was dimension‐selective, as sucrose preference and immobility time remained significantly improved at the same time point. This argues against a generalized loss of NE bioactivity. We interpret the pattern as a quantitative ceiling on downstream intervention. As the disease progresses, escalating peripheral nociceptive drive, ongoing A1 conversion, and rising NET expression collectively outpace the corrective capacity of a fixed‐dose terminal input, while accelerated NE clearance and possible adrenergic receptor desensitization may further blunt late‐phase efficacy. Sensory thresholds, continuously challenged by peripheral tumor‐derived input, are inherently more vulnerable to this ceiling than affective endpoints. Importantly, prazosin and propranolol abolished NE‐mediated rescue across all time points and in both behavioral domains, indicating that adrenergic receptor activation remains the obligatory terminal effector regardless of rescue magnitude. The attenuation, therefore, does not challenge the proposed mechanism. Rather, it reinforces the translational implication that sustained efficacy will likely require multi‐level strategies targeting both the upstream astrocytic driver (LCN2) and the downstream NEergic machinery (e.g., NET), rather than single‐point NE supplementation alone.

Beyond these mechanistic and translational considerations, a separate methodological question concerns how confidently GRAB sensor signals can be interpreted in the context of bone cancer pain–depression comorbidity. The widespread fluorescence reductions we observed across behavioral states could, in principle, arise from altered sensor expression, distribution, or function in diseased tissue rather than from changes in neurotransmitter dynamics per se. Because GRAB sensors report receptor‐accessible extracellular availability rather than vesicular release itself, factors such as membrane trafficking, receptor desensitization, or local diffusion geometry could plausibly contribute to the recorded signals. The morphological remodeling that accompanies A1 reactive transition complicates this interpretation further, since changes in process arborization or perisynaptic coverage could affect sensor accessibility even when molecular expression is preserved. We therefore explicitly acknowledge that a sensor‐side contribution to the observed fluorescence reductions cannot be excluded on the basis of the imaging data alone, and we have approached this concern from several complementary directions, each of which offers partial rather than complete constraint.

The most direct evidence we can offer comes from measuring ligand content with a method that does not depend on the sensor. LC‐MS/MS quantification of PPC tissue showed reduced ATP and Ado content in cancer animals, in the same direction as the GRAB findings and of broadly comparable magnitude. Because mass spectrometry is unaffected by viral expression level, membrane targeting, or astrocytic morphology, it provides ligand‐side evidence that is independent of sensor‐related factors. We note, however, that LC‐MS/MS reports total tissue content rather than the receptor‐accessible extracellular pool detected by GRAB sensors, so the two readouts are complementary rather than strictly equivalent, and their concordance should be regarded as supportive rather than conclusive.

Additional measurements at the protein level partially constrain sensor‐related concerns. Bulk transcript and protein quantification revealed no statistically significant between‐group difference in sensor abundance, although given our cohort size, this comparison is only sensitive to differences of approximately 15% to 20%, and smaller effects cannot be formally excluded. Per‐astrocyte mean intensities and sensor‐to‐GFAP ratios were comparable between groups, consistent with, though not proof of, preservation of total sensor content per cell. We did observe a modest shift in the somatic‐to‐process intensity ratio in cancer animals. Because neither GRAB construct carries compartment‐specific targeting sequences, we tentatively interpret this as passive cytosolic redistribution accompanying astrocytic morphological remodeling, although we cannot exclude the possibility that such redistribution affects sensor accessibility in ways that contribute to the bulk fluorescence reduction. Astrocytic morphological remodeling, including process retraction, hypertrophy, and altered perisynaptic coverage, is a recognized feature of A1 reactive transition and could, in principle, alter the receptor‐accessible extracellular pool independently of total sensor content per cell. We therefore regard this morphological dimension as a particular and unresolved concern, which our current expression‐level measurements constrain only partially. Fiber photometry integrates across hundreds of cells and both somatic and process compartments within the optical volume, which we would expect to attenuate, though not eliminate, the impact of subcellular redistribution on the bulk ΔF/F signal.

The internal pattern of the imaging data offers an additional, though indirect, line of reasoning. The attenuation we report extended across nociceptive (von Frey), depression‐related (tail suspension), home‐cage activity, and rotarod conditions. We accept that this breadth, taken alone, is equally compatible with a tonic ligand‐side shift and with a global sensor‐side reduction in reporting efficiency. The two interpretations can, however, be partially distinguished by the magnitude profile across conditions: reductions were more pronounced in nociceptive and depression‐related contexts than in home‐cage and rotarod recordings, whereas a uniform sensor‐side decrement would, in its simplest form, predict relatively proportional downscaling across all behavioral states. Within this breadth, the changes do not appear to be a simple proportional scaling of all fluorescence metrics, and shape and timing features of individual transients remain relatively preserved even where overall rate or context‐specific amplitude is reduced. In our view, this dissociation is more readily explained by altered release than by a uniform reduction in sensor availability, since a global decrement in sensor expression or accessibility would be expected to attenuate baseline, event amplitude, and event kinetics together. We are nonetheless cautious about this argument: a sensor‐side account with non‐uniform effects on different signal features cannot be formally excluded, and we present this consideration as suggestive rather than definitive. A related point is that, within the linear range of these sensors, ΔF/F normalization is only approximately independent of absolute expression level, and the fluorescence reductions we observe are larger than the maximum expression difference our measurements could plausibly have missed (around 15% to 20%). We regard this as consistent with, but not proof of, a ligand‐side origin for the observed changes.

These considerations constrain but do not eliminate the possibility of a sensor‐side contribution to the observed fluorescence changes, and we recommend that the absolute magnitude of the changes reported here be interpreted with appropriate caution. This caveat is particularly relevant in the context of reactive astrocyte remodeling, where sensor behavior in vivo could be subtly affected in ways that our current measurements would not fully resolve. More definitive resolution of this question would require additional experiments beyond the scope of the present study, including validation with orthogonal biosensors of distinct binding kinetics or high‐temporal‐resolution microdialysis, which we have flagged as priorities for future work.

In conclusion, this study identifies an astrocyte–neuron signaling axis in the PPC in which A1 conversion drives coordinated purinergic and NEergic deficits that underlie cancer pain‐depression comorbidity. Interventions at discrete nodes, from upstream phenotype reversal to downstream A2AR activation and NET inhibition, restored neurochemical homeostasis and alleviated comorbid behaviors, with these effects ultimately converging on adrenergic receptor activation as the common terminal pathway. This extends neuron‐centric models by repositioning astrocytes as active regulators of both sensory and affective processing. The multiple intervention points within this framework provide a rational basis for multi‐target therapy, with priority directions including the investigation of PPC interactions with canonical pain and mood processing circuits and the development of noninvasive delivery methods for clinical translation.

## 4 Methods

### 4.1 Animals

All animal experiments were conducted in accordance with protocols approved by the Ethics Committee of Gansu Provincial Hospital (Approval No. 2025‐094) and complied with the International Association for the Study of Pain ethical guidelines for investigations of experimental pain in animals. Male C57BL/6J mice (8 weeks old; 22–27 g) were obtained from the Lanzhou Veterinary Research Institute of the Chinese Academy of Agricultural Sciences (Lanzhou, China). The *Th*‐Cre mouse line was kindly supplied by Prof. Xiao‐Dong Hu (Lanzhou University). Mice were randomly allocated to experimental groups, with three to five animals housed per cage. All animals were maintained in a specific pathogen‐free, temperature‐controlled facility under a 12‐h light/dark cycle, with ad libitum access to food and water. Prior to behavioral testing, mice were acclimated to the laboratory environment for approximately 1 week. On testing days, animals were habituated to the experimental apparatus for at least 2 h before the onset of behavioral assays. Behavioral observers were blinded to the intervention conditions and group assignments to minimize experimental bias.

### 4.2 Mouse Model

#### 4.2.1 Cell Culture

The murine LLC cell line LL/2 (LLC1)‐luc2 (CTCC‐0499‐Luc2) was obtained from Meisen Cell Technology (Zhejiang, China) on September 1, 2024. This cell line is derived from the parent LL/2 (LLC1) cell line (RRID:CVCL_4358) and has been engineered to express luciferase using the CTCC‐Luc2 plasmid vector, which contains an optimized firefly luciferase (Luc2) gene that utilizes akaLumine as a substrate. Cell authentication was performed using short tandem repeat (STR) profiling on January 17, 2025, confirming the identity of the parent cell line. For this study, we used the LL/2 (LLC1)‐luc2 cell line exclusively, with mycoplasma testing performed on both early and current passages of this cell line. The most recent mycoplasma testing was conducted on March 14, 2025, confirming the cells are mycoplasma‐free. The plasmid map, authentication report, and mycoplasma‐free certification reports for the LL/2 (LLC1)‐luc2 cell line are provided in the Supplementary Materials. Cells were maintained in high‐glucose Dulbecco's modified Eagle medium (DMEM; Gibco, Thermo Fisher Scientific, Waltham, MA, USA) supplemented with 10% fetal bovine serum (Fu Heng, Shanghai, China) and 1% penicillin–streptomycin solution (Sigma–Aldrich, Merck KGaA, Darmstadt, Germany). Cultures were incubated at 37 °C in a humidified atmosphere containing 5% CO_2_.

### 4.3 CDS in the BCP Model

The procedure for LLC cell inoculation was adapted from previously published methods [7]. LLC cells were digested briefly with 0.05% trypsin, followed by centrifugation to remove undigested cell clumps. The resulting cell suspension was adjusted to a final concentration of 2 × 10^5^ cells/mL in PBS. Mice were anesthetized with pentobarbital sodium (50 mg/kg, intraperitoneally), after which the left hind limb was shaved and disinfected with 10% povidone‐iodine followed by 75% ethanol. A 25‐G needle was inserted through the intercondylar notch of the left femur into the medullary cavity, then replaced by a 10‐µL microinjection syringe containing 5 µL of the LLC cell suspension. The cells were injected slowly into the femoral cavity over 2 min. To minimize extracavitary leakage, the injection site was sealed with bone wax. Sham‐operated mice received an equivalent injection of 5 µL PBS without tumor cells. Animals exhibiting surgery‐related motor impairment or lacking tumor growth and bone destruction were excluded from subsequent analyses.

### 4.4 Behavioral Tests

All behavioral assessments were conducted between 08:00 and 17:00 in a quiet, temperature‐ and noise‐controlled environment. To prevent olfactory cues, the testing room and apparatus were carefully cleaned with 10% ethanol between trials. The experimenter remained blind to genotype and treatment allocation throughout the procedures.

### 4.5 Von Frey Test

Mechanical pain threshold was evaluated using the von Frey test, following established protocols [70]. Mice were placed individually in transparent enclosures (5 × 5 × 8 cm^3^) situated on an elevated wire mesh platform. Following a 20–30‐min acclimation period, a blinded experimenter applied a series of calibrated von Frey filaments (0.02, 0.04, 0.07, 0.16, 0.40, 0.60, and 1.00 g; Stoelting) in ascending order of stiffness to the plantar surface of the left hind paw. The paw withdrawal threshold (PWT) was defined as the lowest force that elicited a withdrawal response in at least three of five trials. If fewer than three responses occurred at all forces, the maximum force (1.0 g) was recorded as the PWT.

### 4.6 Hargreaves Test

Paw withdrawal latency (PWL) was assessed using the Hargreaves test, following established protocols. After a 30‐min acclimation period, a radiant heat source was directed at the plantar surface of the left hind paw through a glass platform. PWL was measured to the nearest 0.1 s, with a cutoff time of 20 s to prevent tissue damage. The mean PWL was calculated from three trials conducted at 5–10 min intervals.

### 4.7 Cold Allodynia Test

Cold allodynia was evaluated by applying a drop of acetone (approximately 20–30 µL) to the plantar surface of the hind paw in mice placed individually on an elevated mesh platform. The cumulative duration of nociceptive behaviors (licking or lifting the paw) was recorded for 90 s after acetone application.

### 4.8 Spontaneous Pain Test

To quantify spontaneous pain behaviors, mice were placed individually in transparent chambers with wire mesh floors and video‐recorded for 30 min. Videos were subsequently evaluated in slow motion to manually score the number of flinching or guarding episodes of the left hind paw over a representative 2‐min interval. Behaviors related to locomotion, body repositioning, or grooming were excluded from the analysis.

### 4.9 Sucrose Preference Test (SPT)

Mice were individually housed and acclimated to both 1% sucrose solution and water over a two‐day period, with bottle positions switched every 12 h to avoid side preference. On the day of testing, animals were deprived of food and water for 12 h, after which they were provided with two pre‐weighed identical bottles—one containing water and the other 1% sucrose—for a 12‐h period during the dark phase. Sucrose preference was determined by calculating the ratio of sucrose solution intake to total fluid intake (sucrose solution + water).

### 4.10 Tail‐Suspension Test (TST)

The tails of the mice were wrapped with adhesive tape approximately 1 cm from the tip, and each mouse was suspended upside down from a horizontal bar, with the nose positioned about 30 cm above the floor. Behavioral activity was recorded for a total of 6 min, and immobility time was quantified during the final 4 min of the test.

### 4.11 Forced‐Swim Test (FST)

Each mouse was individually placed in a transparent, vertical glass cylinder (20 cm in diameter, 30 cm in height) filled with water maintained at 22°C–25°C. The water depth was adjusted to ensure that animals could not support themselves by touching the bottom with their limbs or tails. The test was conducted for 6 min, with immobility time measured during the final 4 min. Mice were considered immobile when they remained floating without active movements, except for those necessary to keep their heads above water.

### 4.12 Viruses

All viruses were purchased from BrainVTA Technology (Wuhan, China) or Taitool Bioscience (Shanghai, China). For detailed information regarding the viruses used in this study, see Table S1.

### 4.13 Stereotaxic Surgeries and Viral Microinjection

For stereotaxic surgical procedures, animals were anesthetized with Avertin (250 mg/kg, Sigma–Aldrich). Viral vectors were delivered either unilaterally or bilaterally using a pressure microinjector equipped with a pulled glass capillary. The targeted brain regions varied according to the experimental design and included the PPC (anteroposterior: −1.34 mm, mediolateral: ± 4.00 mm, dorsoventral: −4.15 mm from bregma) and the LC (anteroposterior: −5.05 mm, mediolateral: ± 0.85 mm, dorsoventral: −3.12 mm from bregma). Injection volumes ranged from 200 to 400 nL per hemisphere, administered at a rate of 20 nL/min. Following each injection, the capillary was maintained in situ for an additional 10 min to facilitate viral diffusion and minimize backflow. After surgery, mice were placed on a heating pad to aid recovery from anesthesia. The accuracy of viral injection sites was confirmed by *p*ost‐*hoc* immunohistochemical analysis.

### 4.14 fMOST

We utilized AAV‐c‐Fos‐EYFP vectors delivered through the lateral tail vein (1 × 10^11^ v.g. per mouse in 100 µL PBS) 3 weeks before bone cancer modeling. Following perfusion with 0.1 M PBS and 4% paraformaldehyde after isoflurane anesthesia, brains underwent overnight fixation at 4°C, then embedding within 3%–5% agarose matrix. Whole‐brain fMOST was performed using the BioMapping 9000 system (Wuhan SinoMed, Wuhan, China) with a 10× objective (0.5 NA, LUMPLFLN, Olympus, Tokyo, Japan), dual lasers (473/561 nm), and a sCMOS detector. This approach captured sequential 6‐µm‐thick optical sections through vibratome cutting (77 Hz, 0.2 mm/s), followed by precise light‐sheet scanning. Image processing involved illumination correction, stripe stitching, and standardized preprocessing. Data were registered to Allen Brain Atlas coordinates via Common Coordinate Framework version 3 (CCFV3), enabling automated neural activity quantification through c‐Fos^+^ cell detection. Whole‐brain c‐Fos expression patterns were reconstructed at single‐cell resolution using Imaris software (Oxford Instruments, Abingdon, UK).

### 4.15 Activity‐Dependent Cell Labeling

To identify activity‐dependent cell populations involved in BCP‐induced depressive behaviors, we employed the E‐SARE‐CreERT2 system for targeted labeling. Stereotaxic surgeries were performed by injecting a viral mixture into the PPC. The mixture contained AAV‐E‐SARE‐CreERT2 (200 nL) and AAV‐CAG‐DIO‐EYFP (diluted 1:5, 200 nL). Three weeks post‐surgery, mice were inoculated with LLC cells to establish the BCP model. Before activity‐dependent labeling, all mice were habituated to the behavioral testing environment for three consecutive days to minimize stress‐induced activation. On day 17 after LLC inoculation, we initiated the targeted recombination in active populations protocol. One hour prior to behavioral testing, 4‐hydroxytamoxifen (H6278, Sigma–Aldrich) was dissolved in corn oil and administered intraperitoneally (50 mg/kg). This administration was repeated for three consecutive days to ensure sufficient labeling of cells activated during BCP and comorbid depressive states. Following the labeling protocol, mice were euthanized, and the PPC tissue was collected for immunohistochemical analysis.

### 4.16 Sparse Labeling

To investigate subtle morphological dynamics of astrocytes, glass micropipettes preloaded with a mixture of AAV‐GfaABC1D‐Cre (diluted 1:20 000; 80 nL) and AAV‐DIO‐EGFP (120 nL) were inserted into the unilateral PPC of tumor‐bearing and sham‐operated mice. The viral solution was infused at a rate of 20 nL/min, and the micropipette was maintained in place for 10 min post‐injection to minimize reflux. Following a three‐week expression period, disease modeling was conducted. Brain tissues were harvested for immunofluorescence analysis at days 7, 17, and 21 post‐induction. Coronal sections (45 µm thickness) were prepared using a cryostat, and high‐resolution confocal imaging (4096 × 4096 pixels) was performed. Morphological analyses were conducted using ImageJ software [71].

### 4.17 Neural Circuit Tracing

To anatomically characterize the LC→PPC pathway, two complementary retrograde tracing strategies were employed. For conventional retrograde tracing, CTB‐488 (300 nL) was unilaterally injected into the PPC of C57BL/6J mice using the stereotaxic coordinates and injection parameters described in Section 4.13. Seven days post‐injection, mice were perfused, and LC‐containing sections were immunolabeled for TH to assess co‐localization with CTB‐488 retrograde signal. For cell‐type‐specific retrograde tracing, RetroAAV‐DIO‐mCherry (300 nL) was unilaterally injected into the PPC of *Th*‐Cre mice. Three weeks after injection, LC‐containing sections were co‐labeled for TH to confirm the NEergic identity of mCherry‐labeled neurons projecting to the PPC. Tissue processing, immunolabeling, and imaging procedures were performed as described in Section 4.24.

### 4.18 Sample Preparation

Following behavioral assessments, mice were humanely euthanized under deep CO_2_ anesthesia. The PPC was rapidly dissected, weighed, flash‐frozen in liquid nitrogen, and stored at −80°C until further analysis. The tissue samples were subsequently used for omics profiling, RT‐qPCR, and western blotting.

### 4.19 Metabolomics Analysis

For untargeted metabolomic profiling, hydrophilic metabolites were quantified using an LC‐MS/MS platform, comprising an ultra‐high‐performance liquid chromatography (Vanquish Flex, Thermo Fisher Scientific, Waltham, USA) system coupled to a Q‐Exactive Orbitrap mass spectrometer (Thermo Fisher Scientific). Owing to the limited tissue mass of the PPC and the high sensitivity requirements of LC‐MS/MS, PPC tissues from four mice were pooled to generate each biological replicate. In total, PPC samples from 24 mice were processed for metabolite extraction, yielding three biological replicates per group (each pooled from four mice). Each tissue sample was homogenized with 200 µL H_2_O with five ceramic beads using a mechanical homogenizer. Subsequently, 800 µL of a methanol/acetonitrile (1:1, v/v) was added for metabolite extraction, followed by centrifugation at 14 000 × *g* for 20 min at 4°C. The resulting supernatant was evaporated to dryness in a vacuum concentrator.

For subsequent LC‐MS/MS analysis, dried extracts were reconstituted in 100 µL of acetonitrile/water (1:1, v/v) and centrifuged at 14 000 × *g* for 15 min at 4°C, and the resulting supernatant was injected. Data acquisition was performed in both positive and negative electrospray ionization (ESI) modes. Raw LC‐MS data were processed using XCMS (The Scripps Research Institute, La Jolla, CA, USA) for peak detection, alignment, and quantification. Metabolite identification was achieved by matching accurate m/z values (tolerance < 10 ppm) and MS/MS fragmentation patterns to an in‐house spectral library established with authentic standards.

Statistical analyses included principal component analysis and orthogonal partial least‐squares discriminant analysis to visualize metabolic differences between groups. The variable importance in projection score was used to evaluate the contribution of each metabolite to group separation within the orthogonal partial least‐squares discriminant analysis model. Metabolites exhibiting both *p* < 0.05 and variable importance in projection scores > 1.0 were considered differentially abundant. Differential metabolites were visualized using volcano plots and heat maps and further investigated through metabolic network analysis. Heat maps and unsupervised hierarchical clustering were generated in R statistical software (version 3.6.1). MetaboAnalyst (version 4.0) was employed to examine biological patterns, functional annotations, and pathway enrichments of differentially expressed metabolites. Metabolic network construction and visualization were performed in Cytoscape (version 3.10.3; Cytoscape Consortium, San Diego, CA, USA).

### 4.20 Targeted Quantification of ATP and Ado by LC‐MS/MS

To independently validate the metabolomic findings, ATP and Ado concentrations in the PPC were quantified using a targeted LC‐MS/MS approach. Immediately after dissection, PPC tissues were flash‐frozen in liquid nitrogen. Each biological replicate consisted of pooled tissue from four mice (∼80 mg per pool), with six pooled replicates per group. Frozen tissues were transferred to pre‐chilled 1.5‐mL Eppendorf tubes, homogenized in 200 µL deionized water with five ceramic beads using a mechanical homogenizer at −35°C, and extracted with 800 µL methanol/acetonitrile (1:1, v/v) at −40°C for 1 h. After centrifugation at 12 000 × g for 15 min, the supernatant was collected; the extraction was repeated 3–5 times to ensure complete metabolite recovery. The combined supernatants were lyophilized and stored at −80°C until analysis. The remaining pellets were lysed with RIPA buffer containing protease inhibitors, and protein concentrations were determined by the BCA assay for subsequent normalization.

For LC‐MS/MS analysis, lyophilized samples were reconstituted in pre‐chilled 50% acetonitrile, sonicated at 4°C, and centrifuged at 12 000 × g for 15 min. The supernatant was transferred to injection vials for analysis. Quantification was performed using an internal standard method with calibration curves constructed from authentic ATP and Ado standards. Metabolite concentrations were calculated from peak area integration and expressed as ng/mg protein.

### 4.21 Neuroactive Substance Release Recording

To monitor synchronous release of ATP and Ado, or Ado and NE, AAVs (AAV‐GfaABC1D‐cATP1.0/AAV‐GfaABC1D‐rAdo1.7 or AAV‐GfaABC1D‐rAdo1.7/AAV‐hSyn‐NE2m) were stereotaxically injected into the PPC. An optical fiber (200‐µm diameter; ThinkerTech, Nanjing, China) was implanted above the targeted region. For fluorescence signal detection, ATP and NE signals were excited using a continuous 473‐nm laser at 25  µW, while Ado signals were excited with a continuous 560‐nm laser at 30  µW. The resulting fluorescence was converted to analog voltage signals, digitized at 50  Hz, and recorded using a multichannel fiber photometry recording system (ThinkerTech).

Fluorescence time series were extracted from defined regions of interest, and normalized fluorescence changes (ΔF/F; where ΔF represents the background‐corrected change in fluorescence and F denotes baseline fluorescence) were calculated. Raw fluorescence traces and corresponding heat maps were generated using custom scripts (ThinkerTech) implemented in MATLAB (MathWorks, Natick, MA, USA).

### 4.22 Proteomics Analysis

Proteins were extracted from 24 mouse PPC samples (*n* = 12 per group) using SDT lysis buffer (4% sodium dodecyl sulfate, 100 mM Tris‐HCl, pH 7.6) following protocols optimized for each sample type, and quantified using the bicinchoninic acid assay. For each sample, 15 µg of protein was denatured, separated by SDS–polyacrylamide gel electrophoresis (SDS‐PAGE; 4%–20% gradient, 180 V, 45 min), and visualized with Coomassie Brilliant Blue R‐250. Proteins were digested using the filter‐aided sample preparation method, and the resulting peptides were desalted with C18 reversed‐phase cartridges, dried, resuspended in 40 µL of 0.1% formic acid, and quantified by absorbance at 280 nm. Indexed retention time standard peptides were spiked into each sample prior to analysis.

Peptides were separated using a Vanquish NEO UHPLC system (Thermo Fisher Scientific) and analyzed on an Orbitrap Astral high‐resolution mass spectrometer (Thermo Fisher Scientific) operated in DIA mode. Survey scans (mass‐to‐charge ratio [m/z] 380–980) were acquired at 240 000 resolution (m/z 200), with a normalized automatic gain control target of 500% and a maximum injection time of 5  ms. MS/MS were acquired across 299 variable isolation windows (2  m/z), with higher‐energy collisional dissociation at 25 eV, a normalized automatic gain control target 500%, and a maximum injection time of 3 ms.

DIA data were processed using the DIA‐NN software suite. Search parameters included trypsin as the proteolytic enzyme (allowing a maximum one missed cleavage), carbamidomethylation of cysteine as a fixed modification, and methionine oxidation and protein N‐terminal acetylation as variable modifications. Peptide and protein identifications were filtered at a 1% false discovery rate. Differentially expressed proteins were identified by applying a threshold of *p* < 0.05 and a minimum |log_2_(Fold change)| of 1.2 in comparisons between the sham and cancer groups. Feature proteins meeting these criteria were subjected to further analysis. Heat maps and hierarchical clustering were generated in R statistical software to visualize expression patterns across groups. Functional annotation of differentially expressed proteins was performed using Blast2GO (BioBam Bioinformatics S.L., Valencia, Spain) with BLASTP (version 2.8.0+; National Center for Biotechnology Information, Bethesda, MD, USA), and protein–protein interaction networks were constructed using the Search Tool for the Retrieval of Interacting Genes/Proteins database.

### 4.23 Real‐Time Reverse Transcription Quantitative PCR (RT‐qPCR)

Mouse PPC tissues were homogenized in TRIzol reagent (Invitrogen, Thermo Fisher Scientific) on ice, and total RNA was isolated following the manufacturer's protocol (Takara Bio Inc., Shiga, Japan). For complementary DNA (cDNA) synthesis, 1 µg of total RNA was reverse transcribed using a commercially available kit (Takara Bio Inc.) according to the manufacturer's instructions. RT‐qPCR was performed with the TB Green Premix Ex Taq II kit (Takara Bio Inc.) on a QuantStudio 3 Real‐Time PCR System (Thermo Fisher Scientific). Each biological sample was analyzed in triplicate. Amplification and quantification data were acquired and processed using QuantStudio software (Applied Biosystems, Thermo Fisher Scientific). Glyceraldehyde‐3‐phosphate dehydrogenase (GAPDH) was used as the endogenous control. Primer sequences (synthesized by Sangon Biotech, Shanghai, China) are provided in Table S2. Relative gene expression levels were calculated using the 2^−ΔΔCT^ method.

### 4.24 Western Blotting

Mouse brain tissues were homogenized in pre‐chilled radioimmunoprecipitation assay lysis buffer supplemented with a protease inhibitor cocktail, followed by incubation on ice for 30 min. Homogenates were centrifuged at 10 000 × *g* for 20 min at 4°C to obtain protein lysates. Equal amounts of protein were separated by SDS‐PAGE and transferred to PVDF membranes (Millipore, Merck KGaA). Membranes were blocked with 5% skim milk in tris‐buffered saline with 0.1% Tween‐20 (TBST) for 1 h at room temperature and then incubated overnight at 4°C with primary antibodies (see Table S3 for detailed information on antibodies used in this study). After three washes in tris‐buffered saline with 0.1% Tween‐20, membranes were incubated with horseradish peroxidase‐conjugated secondary antibodies for 1 h at room temperature. Protein bands were detected and quantified using ImageJ software, with expression levels normalized to GAPDH. Uncropped western blot images are provided in the Source Data file.

### 4.25 Cannula Infusion Experiment

A custom‐designed cannula (Kedou Brain‐computer Technology, Suzhou, China), consisting of a hollow catheter (outer diameter: 0.5 mm) and a protective cap housing an inner core (outer diameter: 0.25 mm) was bilaterally implanted into the PPC of male C57BL/6J mice (8 weeks old). Following surgery, a dummy cannula extending 0.5 mm beyond the guide cannula and secured with a metal cap was inserted to maintain patency. One week after implantation, the BCP model was established by inoculating the mice with LLC cells.

On day 3, 7, 10, 14, 17 after BCP induction, experimental drugs and chemicals (see Table S4 for detailed information on drugs used in this study) were bilaterally infused into the PPC (0.3 µL per hemisphere) of freely moving mice. Infusions were performed via an inner injection core connected to a microinfusion pump at a rate of 0.05 µL/min for three consecutive days. Minocycline was administered using the same protocol for seven consecutive days. To ensure local drug diffusion and minimize backflow, the injector core was left in place for an additional 5 min following infusion. Behavioral testing or fiber photometry was conducted 30 min after drug delivery. Only mice with verified injection sites, confirmed by Nissl staining, were included in the analysis.

### 4.26 Immunohistochemistry and Imaging

Mice were deeply anesthetized with isoflurane and transcardially perfused with ice‐cold 0.9% saline, followed by fixation with 4% paraformaldehyde. Brains were carefully extracted and post‐fixed overnight at 4°C in 4% paraformaldehyde in PBS. For cryoprotection, the brains were sequentially incubated in 20% (w/v) and then 30% (w/v) sucrose in PBS at 4°C until fully submerged. Coronal sections (30 µm thick) were prepared using a Leica CM1950 cryostat (Leica, Wetzlar, Germany). For immunofluorescence staining, free‐floating sections were blocked in PBS containing 0.3% Triton X‐100 and 10% donkey serum for 1 h at room temperature. Sections were then incubated overnight at 4°C with primary antibodies diluted in blocking buffer (see Table S3 for detailed information on antibodies used in this study). After three 5‐min washes in PBS, sections were incubated with the corresponding fluorophore‐conjugated secondary antibodies for 1.5 h at room temperature. Nuclei were counterstained with 4′,6‐diamidino‐2‐phenylindole (DAPI, 10 µg/mL, C0065, Beijing Solarbio Science & Technology Co., Ltd., Beijing, China) before mounting. Fluorescent images were acquired using a Leica SP8 confocal microscope. For quantification, all sample identifiers were coded prior to analysis to ensure blinding.

### 4.27 Statistical Analysis

Mice were randomly assigned to experimental groups, and all analyses were performed in a blinded manner. No data transformation or normalization was applied prior to analysis. Outliers were identified using the ROUT method (Q = 1%) and are noted in the Source Data file. Animals with mistargeted viral injections or drug delivery, as verified by post‐hoc histological examination, were excluded from subsequent analyses. All data are presented as mean ± SEM. Statistical significance is indicated as ^*^ *p* < 0.05, ^**^ *p* < 0.01, and ^***^ *p* < 0.001. For multi‐timepoint comparisons in Figure 2H, the following symbols are used: ^*^ *p* < 0.05, ^**^ *p* < 0.01 for sham vs. cancer 7d; ^#^ *p* < 0.05, ^##^ *p* < 0.01, ^###^ *p* < 0.001 for sham vs. cancer 17d; ^+^ *p* < 0.05, ^++^ *p* < 0.01 for sham vs. cancer 21d; ^@^ *p* < 0.05, ^@@^ *p* < 0.01 for cancer 7d vs. cancer 21d; ^$^ *p* < 0.05 for cancer 7d vs. cancer 17d. *P*‐values below 0.0001 are not reported as exact values. Sample sizes were determined based on preliminary experiments and comparable published studies. The number of animals or experimental replicates (n) for each statistical analysis, along with detailed statistical outcomes including exact *p*‐values and degrees of freedom, is provided in the corresponding figure legends and the Source Data file. Western blotting and brain morphological experiments were independently replicated at least three times with consistent results. Prior to statistical analysis, all datasets were assessed for normality using the Shapiro–Wilk test and for homogeneity of variance using Levene's test. For data meeting parametric assumptions, comparisons between two groups were performed using a two‐tailed unpaired Student's *t*‐test; comparisons involving more than two groups were performed using one‐way or two‐way analysis of variance (ANOVA) followed by Bonferroni's post‐hoc test. All statistical tests were two‐sided, with a significance level of α = 0.05 (95% confidence level). No additional alpha adjustment was applied unless otherwise noted in the figure legends.

To examine correlations between pathological indicators (A1 astrocyte polarization ratio, ATP, Ado, and norepinephrine levels in the PPC) and behavioral outcomes (PWT, PWL, CA, SP, SPT, TST immobility, and FST immobility), Pearson correlation coefficients (*r*) were computed. To capture continuous biological relationships within the pathological state itself and to avoid spurious correlations driven by systematic group‐level separation between sham and cancer animals, correlation analyses were performed exclusively on data from individual cancer‐group mice; sham animals were not included. Correlation strength was interpreted as weak (|r| < 0.4), moderate (0.4 ≤ |r| ≤ 0.7), or strong (|r| ≥ 0.7). Results are presented as forest plots displaying the Pearson *r* with 95% confidence intervals, with statistical significance set at *p* < 0.05 (two‐tailed). Statistical methods for metabolomic and proteomic data are described in their respective sub‐sections. All statistical analyses were performed using GraphPad Prism 9.2.0 (GraphPad Software, San Diego, CA, USA).

## Funding

This work was supported by grants from the National Natural Science Foundation of China (82360236 to J.L.), Gansu Provincial Natural Science Foundation (23JRRA1299 to J.L.), Youth Talent Project of Gansu Province (2025QNGR67 to J.L.), Science and Technology Plan Project of Lanzhou (2023‐4‐59 to J.L.), Research Project of Gansu Provincial Hospital (23GSSYA‐5 to J.L.), the Major Project of Gansu Provincial Department of Science and Technology Joint Research Fund (25JRRA1195 to E.C.), Key R&D Program of Gansu Province Science and Technology Program (24YFFA031 to C.W.), and Excellent Graduate Student Cultivation Project of Gansu Provincial Hospital (22GSSYD‐57 to Z.T.).

## Conflicts of Interest

The authors declare no conflicts of interest.

## Supporting information




**Supporting File 1**: advs75972‐sup‐0001‐SuppMat.docx.

**Supporting File 2**: advs75972‐sup‐0002‐Data1.xlsx.

**Supporting File 3**: advs75972‐sup‐0003‐MovieS1.mp4.

## Data Availability

All data supporting the findings of this study are available within the main text and the Supplementary Information. Source data for Figures 1, 2, 3, 4, 5, 6, 7, 8, 9, 10, 11, 12 and Figures S2–10 are provided with this paper. There are no restrictions on data availability.
