# Supplementary material for: Astrocytic Phenotypic Switching in Posterior Piriform Cortex Orchestrates Bone Cancer Pain–Depression Comorbidity via Purinergic–Noradrenergic Signaling
Source: Adv Sci (Weinh). 2026 Jun 5:e23150. Online ahead of print. doi: 10.1002/advs.202523150 (PMC13336383; doi:10.1002/advs.202523150)
Supplement: Supplementary file 1 — Supporting File 1: advs75972‐sup‐0001‐SuppMat.docx. [file ADVS-9999-e23150-s002.docx]

Supporting Information

**Astrocytic Phenotypic Switching in Posterior Piriform Cortex Orchestrates Bone Cancer Pain**–**Depression Comorbidity via Purinergic–Noradrenergic Signaling**

Jiang-Ping Liu† ⃰, Jun-Han Zhang†, Zhi-Xuan Tan†, Wei-Bing Yan, Ya-Ru Yuan, Yao Liu, Bing-Di Wei, Meng-Qi Ding, Zhuo-Min Fu, Ying Liu, An-Qi Wang, Lin Ma, Yong-Hong Li ⃰, Er-Qing Chai ⃰, Chao-Jun Wei ⃰

**Correspondence:** J.-P.L. (liujiangping@pku.org.cn), Y.-H.L. (haifenglyh@126.com), E.-Q.C. (happybirds998@126.com), and C.-J.W. (weichaojun-gsph@hotmail.com).

**This file includes:**

**Fig S1-S10**

**Tables S1–S4**

**
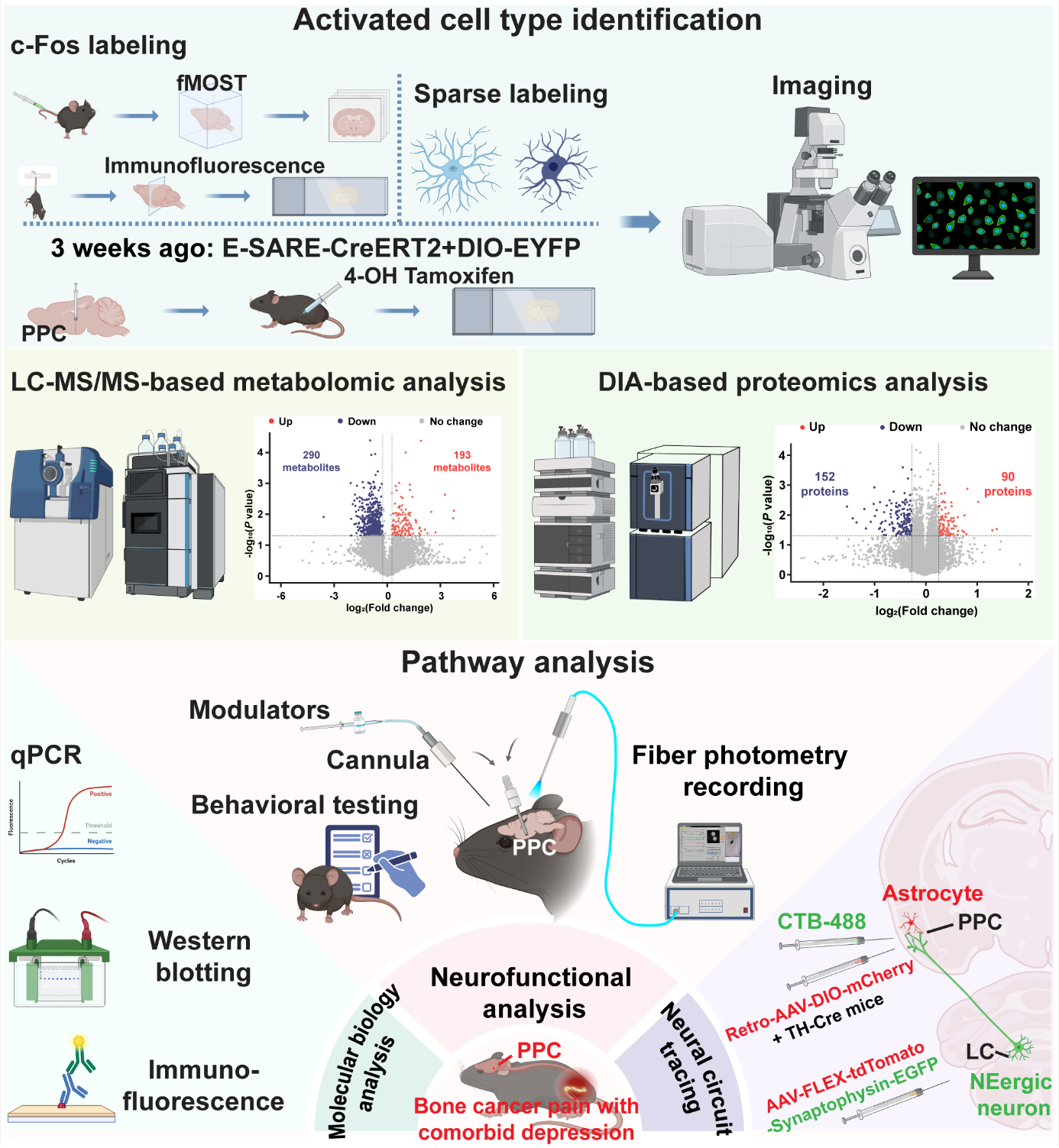
Figure S1. Graphical overview of the experimental strategy.** Schematic illustrating the multi-level experimental approach used in this study. Top: Activated cell type identification in the PPC, including whole-brain c-Fos mapping via fMOST imaging and immunofluorescence, combined with sparse labeling using E-SARE-CreERT2 + DIO-EYFP and 4-hydroxytamoxifen to identify activated cell populations. Middle: Multi-omics profiling of PPC tissue, including LC-MS/MS-based metabolomic analysis and DIA-based proteomic analysis in bone cancer pain mice compared with sham controls. Bottom: Pathway analysis and functional validation strategies, encompassing molecular biology approaches (qPCR, western blotting, and immunofluorescence), neurofunctional analyses (pharmacological modulation via cannula, behavioral testing, and fiber photometry recording of NE dynamics in the PPC), and neural circuit tracing (CTB-488 retrograde tracing, Retro-AAV-DIO-mCherry in *Th*-Cre mice, and AAV-FLEX-tdTomato-Synaptophysin-EGFP anterograde tracing) to delineate the LC–PPC NEergic circuit and its interaction with astrocytes in BCP with CDS. Created with BioRender.com.


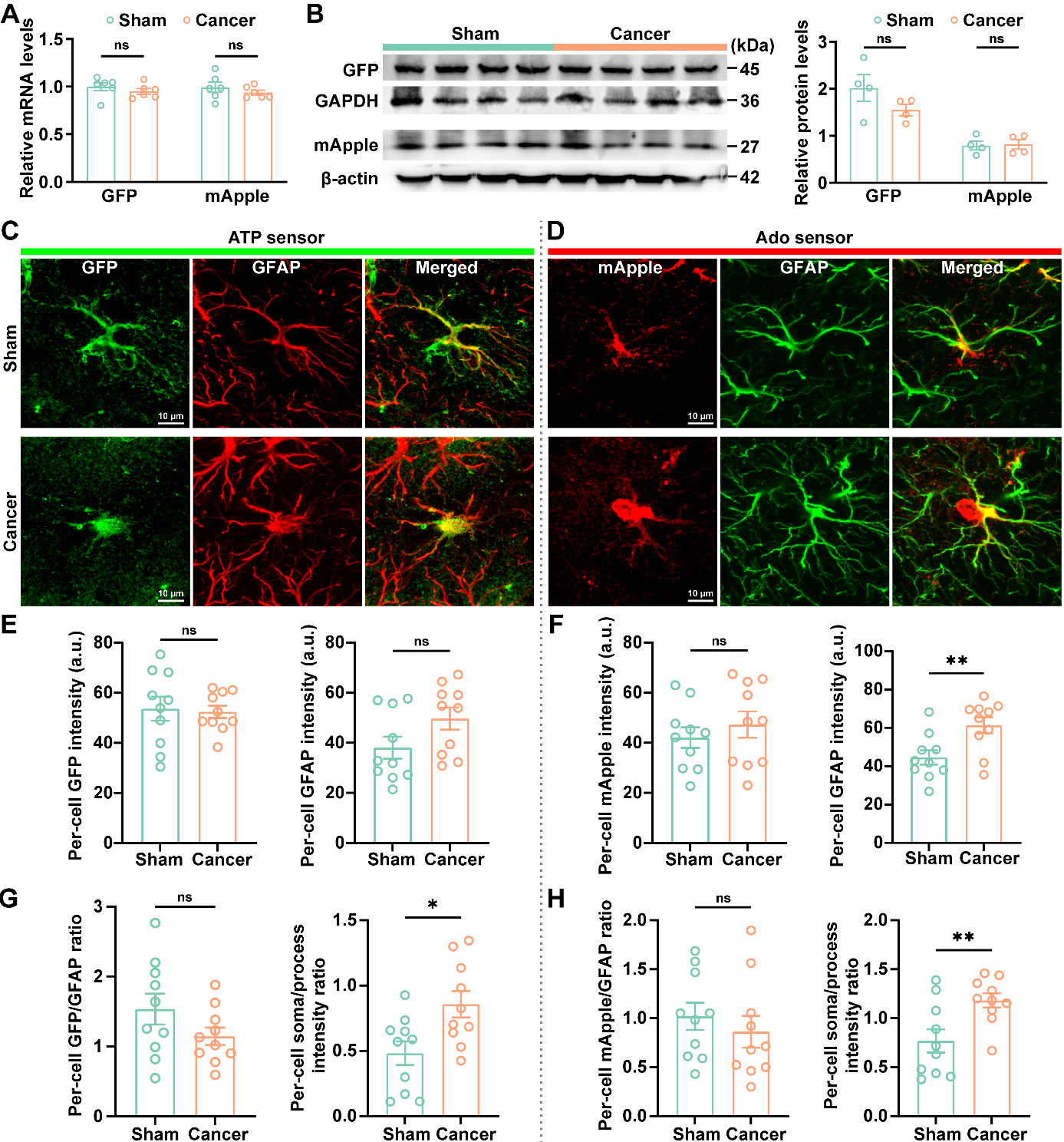


**Figure S2. Comparable expression and astrocyte localization of GRAB-ATP and GRAB-Ado sensors in sham and cancer mice.** (A) qRT-PCR of GFP (GRAB-cATP1.0) and mApple (GRAB-rAdo1.7) mRNA in PPC tissue, showing equivalent viral transduction between groups. (ns, not significant; *n* = 6 pooled samples per group, each pooled from 4 mice). (B) Representative western blots (left) and quantification (right) of GFP (~45 kDa) and mApple (~27 kDa), with GAPDH and β-actin as loading controls. Sensor protein levels were comparable between groups. (ns, not significant; *n* = 4 pooled samples per group, each pooled from 4 mice). (C, D) Representative confocal images showing co-localization of GRAB-ATP (C; GFP, green) and GRAB-Ado (D; mApple, red) sensors with GFAP immunostaining in PPC astrocytes from sham (top) and cancer (bottom) mice. Scale bars, 10 μm. (E, F) Per-cell quantification of sensor (left) and GFAP (right) fluorescence intensities for ATP (E) and Ado (F) cohorts. (G, H) Per-cell sensor/GFAP intensity ratio (left) and soma-to-process intensity ratio (right) for ATP (G) and Ado (H) cohorts. n = 10 cells/3 mice per group for (E)–(H). **p* < 0.05, ***p* < 0.01. Significance was assessed using unpaired two-tailed Student’s *t*-test for (E-H). Data are presented as mean ± SEM. Exact *p*-values, degrees of freedom, and pairwise comparisons are provided in the Source Data file.


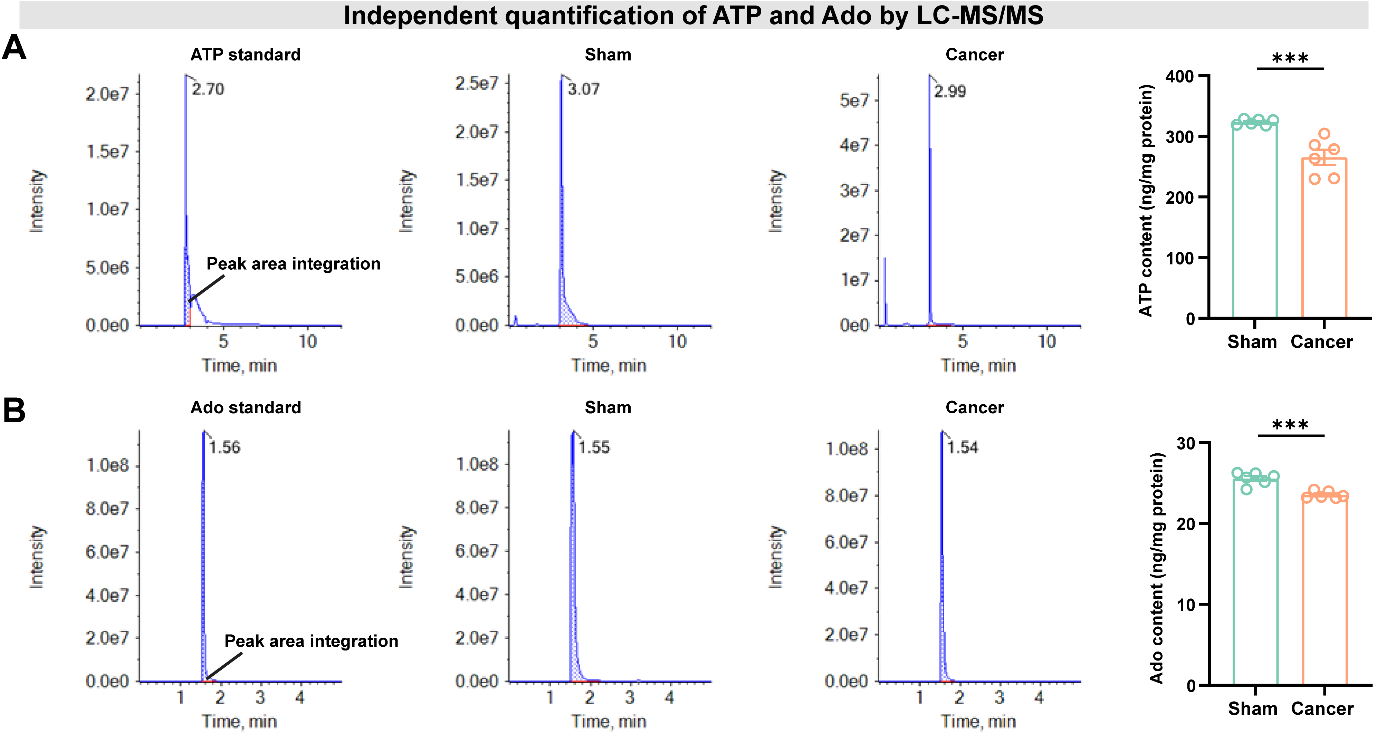


**Figure S3. LC-MS/MS-based absolute quantification confirms decreased ATP and Ado levels in the PPC of cancer mice.** (A) LC-MS/MS-based absolute quantification of ATP in PPC tissue. Representative extracted ion chromatograms are shown for the pure standard, sham, and cancer samples, with retention times labeled above each peak. Analyte concentrations were determined by peak area integration: the area under each chromatographic peak was calculated and mapped to a calibration curve generated from serial dilutions of the corresponding standard, thereby converting peak area values into absolute content (ng per mg protein). The chromatographic peaks of cancer samples were visibly smaller than those of sham samples (retention time ~2.99–3.07 min), consistent with the quantification results (rightmost) showing significantly decreased ATP content in the PPC of cancer mice (*n* = 6 pooled samples per group, each pooled from 4 mice). (B) LC-MS/MS-based absolute quantification of Ado in PPC tissue. Displayed as in (A). The chromatographic peaks of cancer samples were visibly smaller than those of sham samples (retention time ~1.54–1.55 min), consistent with the quantification results (rightmost) showing significantly decreased Ado content in the PPC of cancer mice (*n* = 6 pooled samples per group, each pooled from 4 mice). ****p* < 0.001. Significance was assessed using unpaired two-tailed Student’s *t*-test for (A) and (B). Data are presented as mean ± SEM. Exact *p*-values, degrees of freedom, and pairwise comparisons are provided in the Source Data file.

**
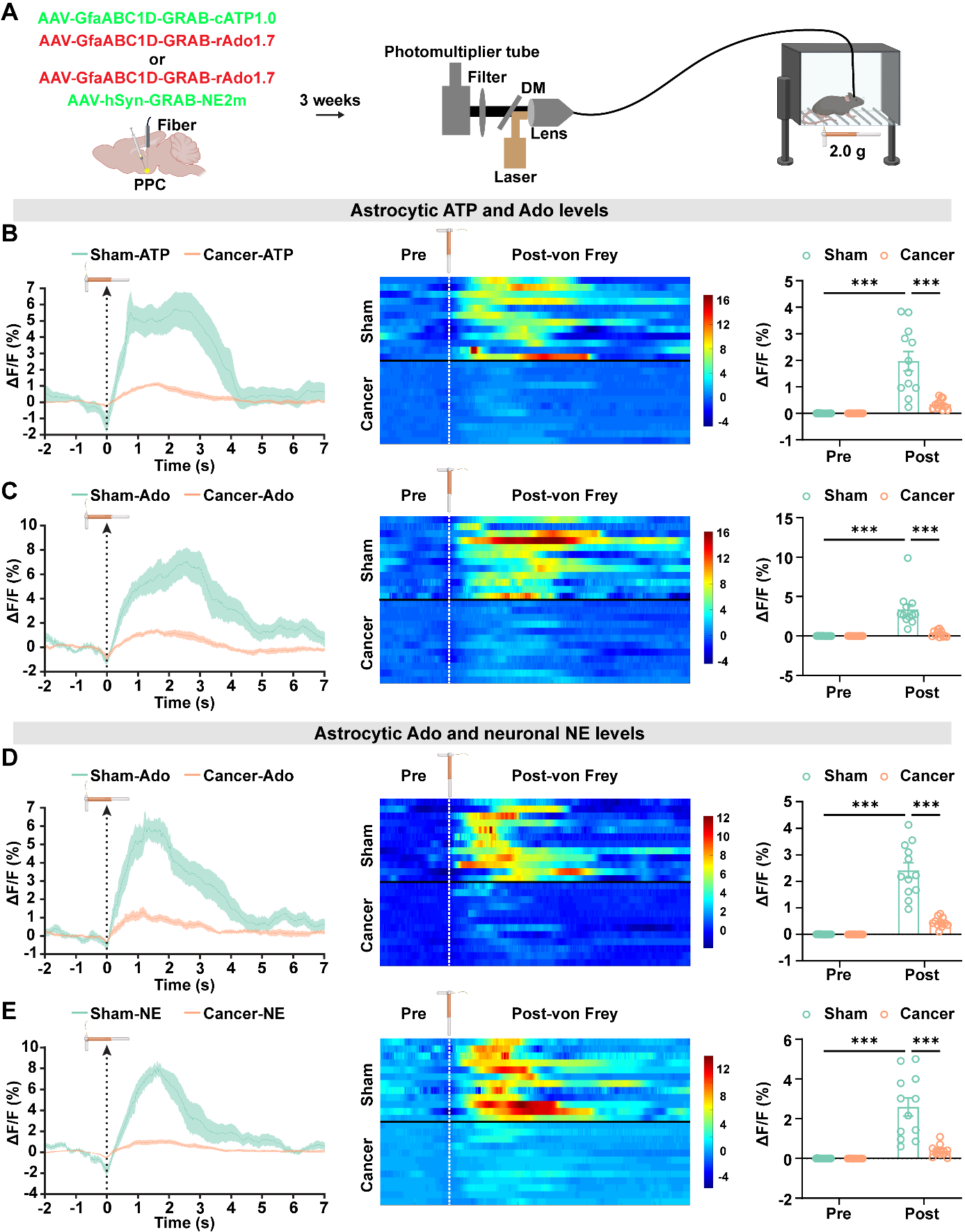
**

**Figure S4. Astrocytic ATP, Ado, and neuronal NE dynamics in the PPC during von Frey stimulation.** (A) Schematic of the experimental strategy. AAV-GfaABC1D-GRAB-cATP1.0, AAV-GfaABC1D-GRAB-rAdo1.7, or a combination of AAV-GfaABC1D-GRAB-rAdo1.7 and AAV-hSyn-GRAB-NE2m was delivered into the PPC with fiber implantation. After 3 weeks of viral expression, fiber photometry recordings were performed during von Frey filament stimulation (2.0 g). (B) Astrocytic ATP dynamics in the PPC during von Frey stimulation. Left, average ΔF/F (%) traces aligned to filament application (dashed line); shaded areas represent SEM. Middle, heatmaps of ΔF/F for individual trials. Right, quantification of mean ΔF/F (%) during pre- and post-stimulation epochs (*n* = 12 mice per group). (C) Astrocytic Ado dynamics in the PPC during von Frey stimulation, displayed as in (B) (*n* = 12 mice per group). (D) Astrocytic Ado dynamics in the PPC during von Frey stimulation, recorded simultaneously with neuronal NE shown in (E), displayed as in (B) (*n* = 12 mice per group). (E) Neuronal NE dynamics in the PPC during von Frey stimulation, recorded simultaneously with astrocytic Ado shown in (D), displayed as in (B) (*n* = 12 mice per group). Astrocytic ATP, astrocytic Ado, and neuronal NE responses to mechanical stimulation were all significantly attenuated in cancer mice compared with sham controls. ****p* < 0.001. Significance was assessed using two-way ANOVA with Bonferroni’s *post-hoc* test. Data are presented as mean ± SEM. Exact *p*-values, degrees of freedom, and pairwise comparisons are provided in the Source Data file. Created with BioRender.com.

**
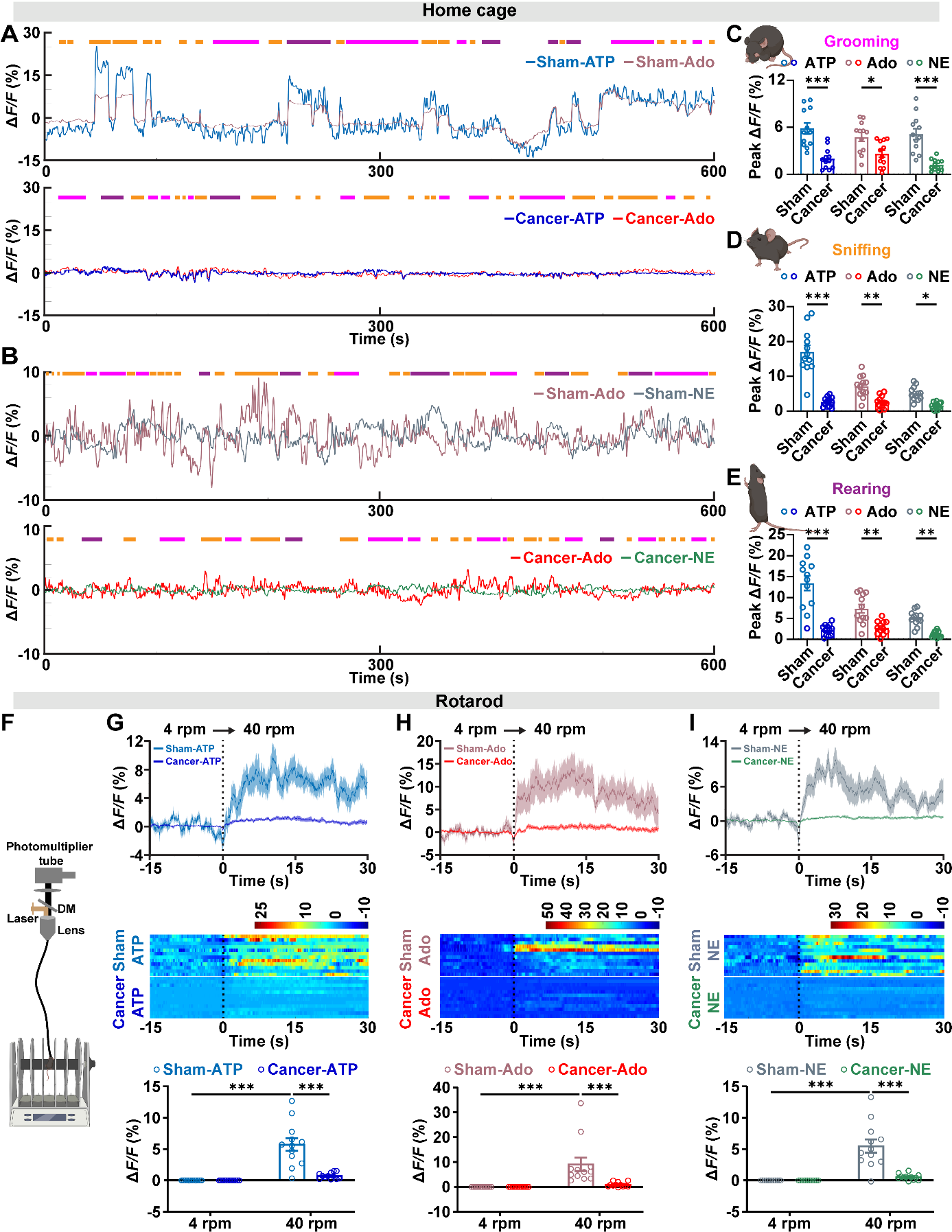
**

**Figure S5. Diminished PPC astrocytic ATP, Ado, and neuronal NE dynamics during naturalistic behaviors and motor challenge in cancer mice.** (A) Representative dual-color fiber photometry traces of concurrent ATP (cyan/blue) and Ado (mauve/red) signals during 10-min home cage sessions in sham (top) and cancer (bottom) mice. Colored bars above traces denote grooming (magenta), sniffing (orange), and rearing (dark purple) episodes. (B) Representative dual-color fiber photometry traces of concurrent Ado (mauve/red) and NE (gray-green/green) signals during 10-min home cage sessions in sham (top) and cancer (bottom) mice. Colored bars are as in (A). (C) Peak ΔF/F (%) for ATP, Ado, and NE signals time-locked to grooming episodes. Cancer mice showed significantly reduced responses across all three transmitters (*n* = 12 mice per group). (D) Peak ΔF/F (%) for ATP, Ado, and NE signals time-locked to sniffing episodes. Cancer mice showed significantly reduced responses across all three transmitters (*n* = 12 mice per group). (E) Peak ΔF/F (%) for ATP, Ado, and NE signals time-locked to rearing episodes. Cancer mice showed significantly reduced responses across all three transmitters, indicating generalized suppression of PPC signaling beyond pain-evoked contexts (*n* = 12 mice per group). (F) Schematic of the fiber photometry system illustrating the optical path: laser, dichroic mirror (DM), fiber-coupled lens, and photomultiplier tube. (G) ATP dynamics in the PPC during accelerating rotarod testing (4 → 40 rpm). Top, averaged ΔF/F (%) traces aligned to acceleration onset (dashed line); shaded areas represent SEM. Middle, trial-by-trial heatmaps for sham and cancer groups. Bottom, mean ΔF/F (%) quantification at 4 rpm versus 40 rpm (*n* = 12 mice per group). (H) Ado dynamics in the PPC during accelerating rotarod testing, displayed as in (G) (*n* = 12 mice per group). (I) NE dynamics in the PPC during accelerating rotarod testing, displayed as in (G) (*n* = 12 mice per group). Sham mice showed robust signal increases upon acceleration, whereas cancer mice displayed markedly blunted responses despite comparable baselines. **p* < 0.05, ***p* < 0.01, ****p* < 0.001. Significance was assessed by two-way ANOVA with Bonferroni's *post-hoc* test. Data are presented as mean ± SEM. Exact *p*-values, degrees of freedom, and pairwise comparisons are provided in the Source Data file. Created with BioRender.com.

**
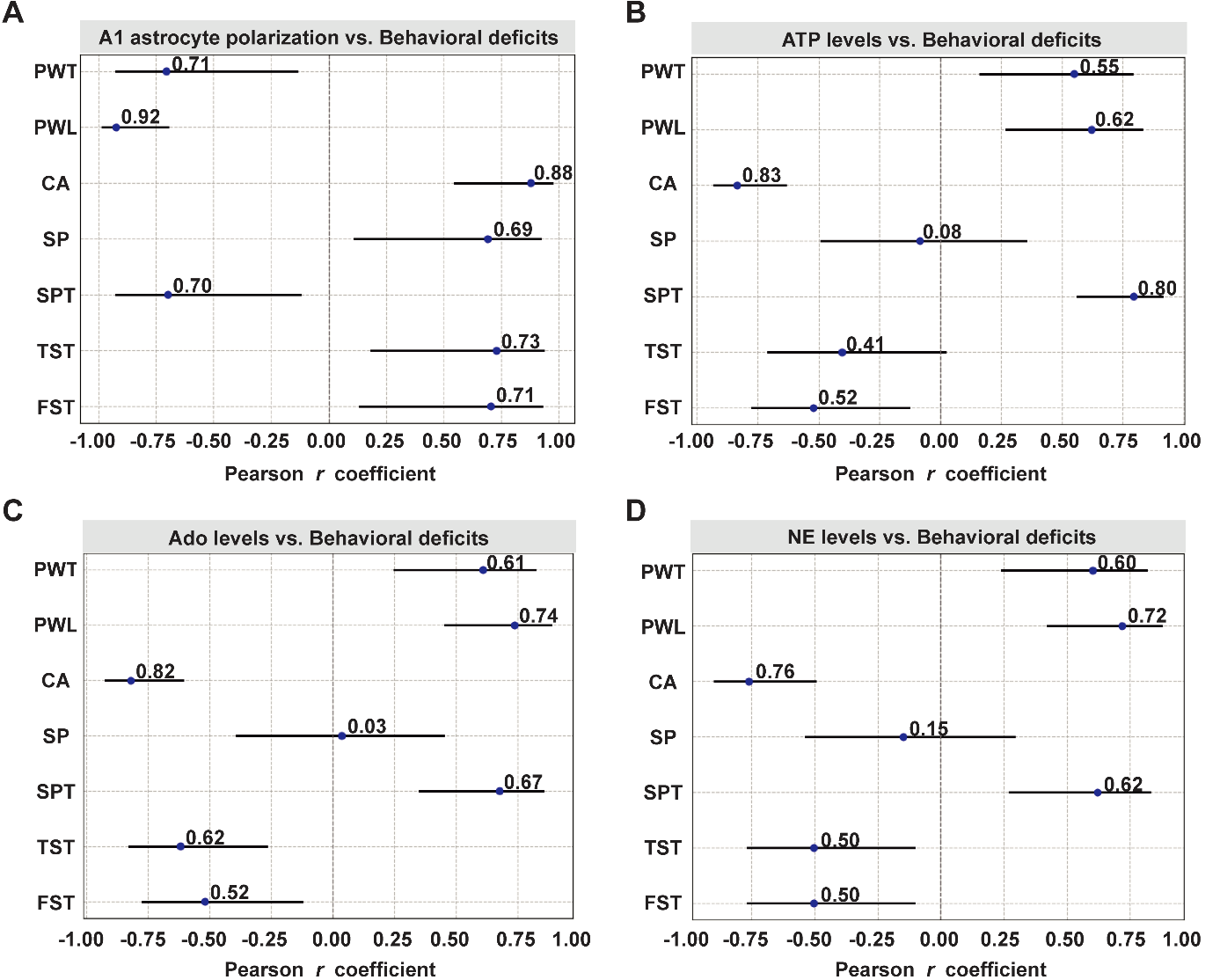
**

**Figure S6. Correlations between A1 astrocyte polarization, neurotransmitter levels, and behavioral deficits in cancer-group mice.** All Pearson correlations were performed within the cancer group only to assess continuous biological relationships across individuals in the pathological state, thereby avoiding artifactual correlations driven by sham–cancer group separation. (A) Forest plot showing Pearson correlation coefficients (*r*) with 95% confidence intervals (CIs) between behavioral measures and A1 astrocyte polarization index in the PPC. Behavioral measures include paw withdrawal threshold (PWT), paw withdrawal latency (PWL), cold allodynia (CA), spontaneous pain (SP), sucrose preference test (SPT), tail suspension test (TST), and forced swim test (FST). A1 polarization was negatively correlated with pain thresholds (PWT, PWL) and sucrose preference, and positively correlated with pain- and depression-related behaviors (CA, SP, TST, FST). (B) Forest plot showing Pearson *r* with 95% CIs between behavioral measures and astrocytic ATP levels in the PPC. ATP levels were positively correlated with pain thresholds and sucrose preference, and negatively correlated with aversive and despair-like behaviors. (C) Forest plot showing Pearson *r* with 95% CIs between behavioral measures and astrocytic Ado levels in the PPC. Ado levels showed a similar correlation pattern to that of ATP. (D) Forest plot showing Pearson *r* with 95% CIs between behavioral measures and neuronal NE levels in the PPC. NE levels were positively correlated with pain thresholds and sucrose preference, and negatively correlated with aversive and despair-like behaviors. Across (B)–(D), SP showed relatively weak correlations with ATP, Ado, and NE levels, suggesting that spontaneous pain may involve additional mechanisms beyond PPC neurotransmitter signaling. Pearson correlation analysis was used throughout; values indicate *r* coefficients and horizontal lines represent 95% CIs. Exact *r*-values, *p*-values, sample sizes, and 95% CIs for each correlation are provided in the Source Data file. Created with BioRender.com.


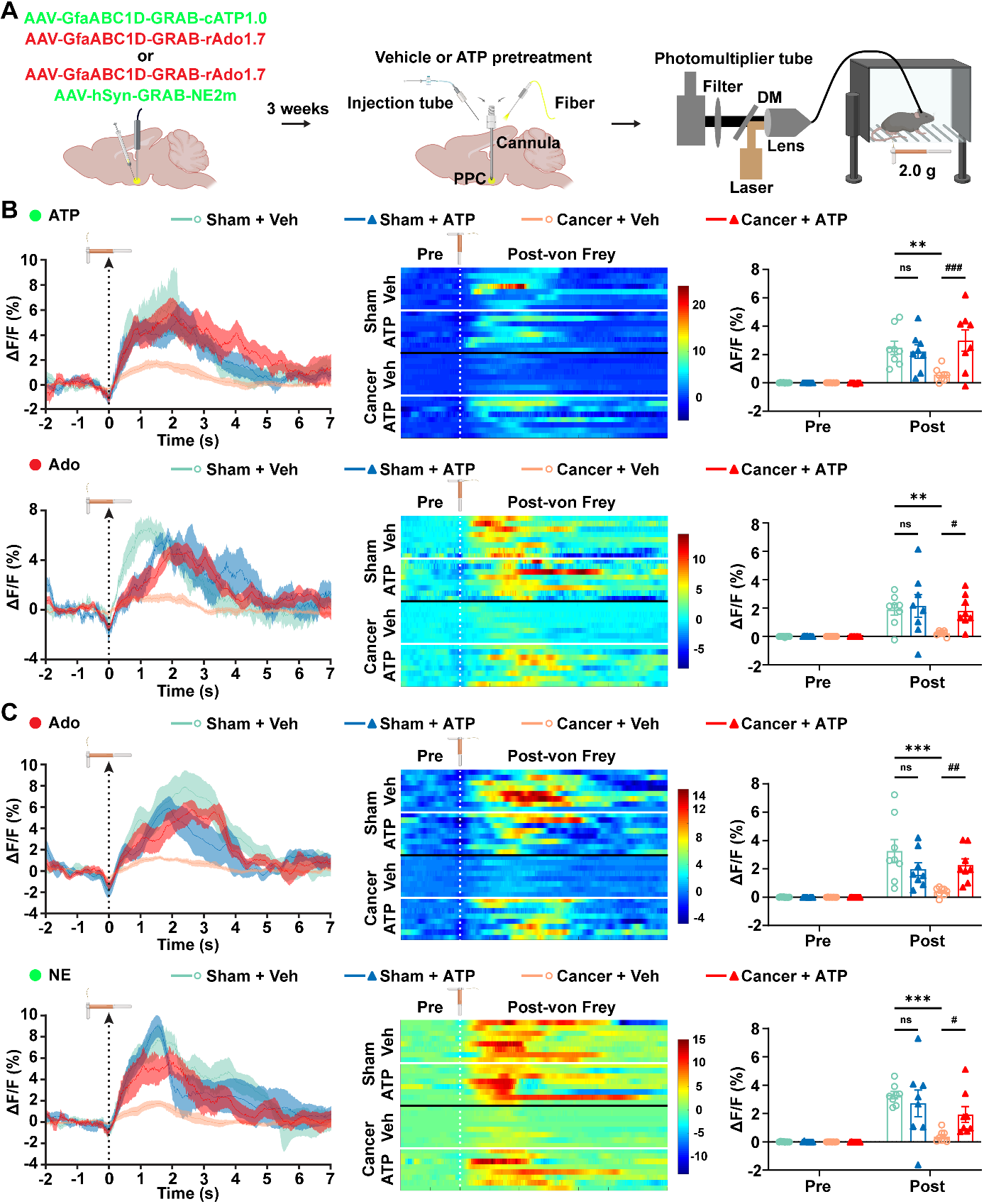


**Figure S7. Local ATP supplementation in the PPC rescues diminished astrocytic purinergic and neuronal NEergic responses to mechanical stimulation in cancer mice.** (A) Experimental design. Mice were injected with either AAV-GfaABC1D-GRAB-cATP1.0 and AAV-GfaABC1D-GRAB-rAdo1.7 (for simultaneous ATP and Ado recording) or AAV-GfaABC1D-GRAB-rAdo1.7 and AAV-hSyn-GRAB-NE2m (for simultaneous Ado and NE recording) into the PPC. After 3 weeks of viral expression, a cannula was implanted adjacent to the optical fiber for local vehicle or ATP pretreatment. Von Frey filament (2.0 g) stimulation was applied during fiber photometry recording. Right, schematic of the photomultiplier tube-based detection system. (B) Concurrent astrocytic ATP (top) and Ado (bottom) dynamics in the PPC during von Frey stimulation across sham + Veh, sham + ATP, cancer + Veh, and cancer + ATP groups. Left, averaged ΔF/F (%) traces (mean ± SEM) aligned to von Frey onset (dashed line). Middle, trial-by-trial heatmaps for pre- and post-stimulation periods. Right, mean ΔF/F (%) quantification. ATP pretreatment significantly rescued both astrocytic ATP and Ado responses in cancer mice without altering sham responses (*n* = 8 mice per group). (C) Concurrent astrocytic Ado (top) and neuronal NE (bottom) dynamics in the PPC during von Frey stimulation across the same four groups as in (B). Displayed as in (B). Local ATP pretreatment similarly restored both Ado and NE responses in cancer mice to levels comparable to those of sham controls (*n* = 8 mice per group). ***p* < 0.01, ****p* < 0.001 for sham + Veh vs. cancer + Veh; ^#^*p* < 0.05, ^##^*p* < 0.01, ^###^*p* < 0.001 for cancer + Veh vs. cancer + ATP; ns, not significant for sham + Veh vs. sham + ATP. Significance was assessed using two-way ANOVA with Bonferroni’s *post-hoc* test. Data are presented as mean ± SEM. Exact *p*-values, degrees of freedom, and pairwise comparisons are provided in the Source Data file. Created with BioRender.com.


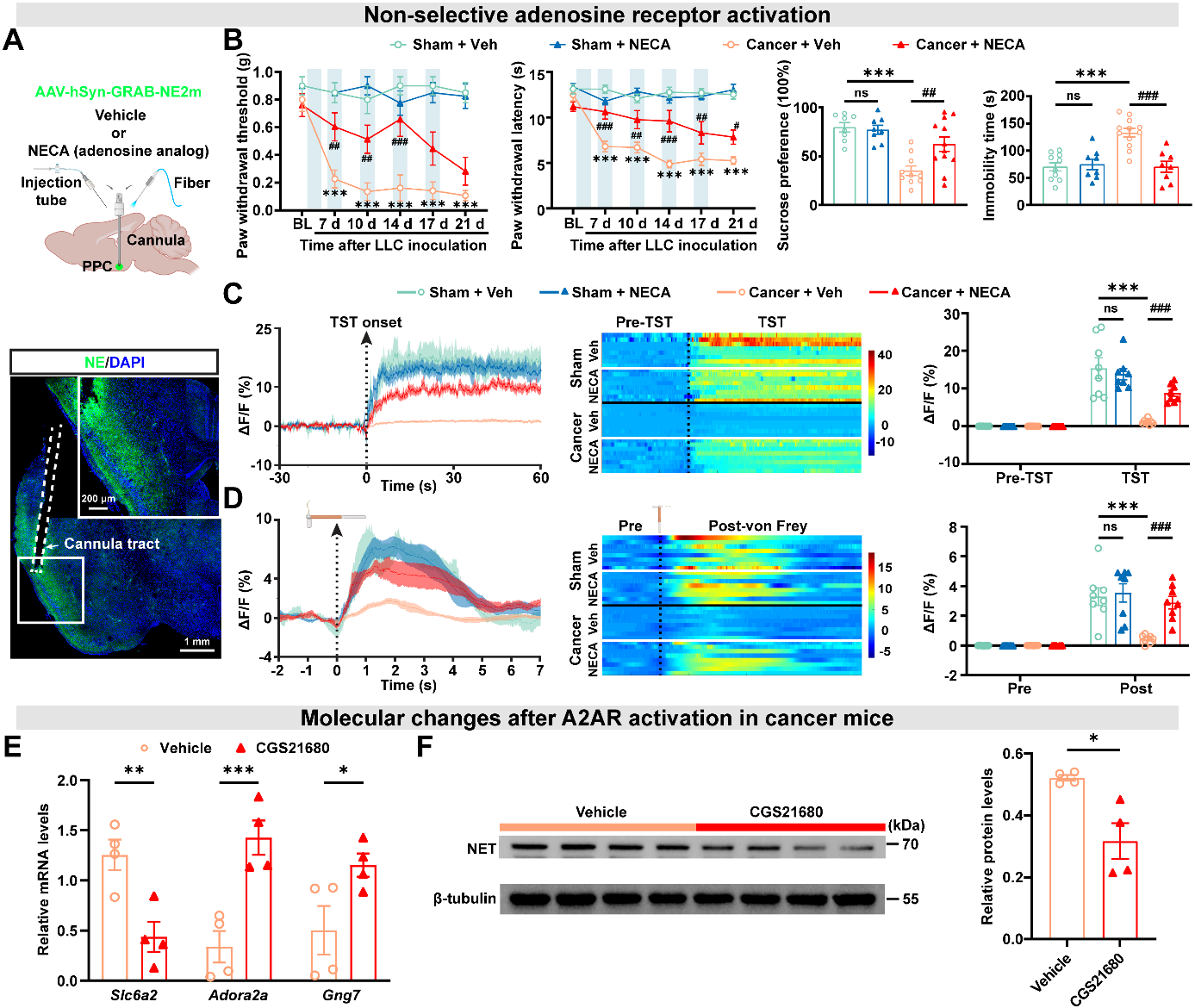


**Figure S8. PPC adenosine receptor activation rescues cancer-induced behavioral and NEergic deficits and modulates NET expression.** (A) Experimental design. AAV-hSyn-GRAB-NE2m was injected into the PPC with cannula implantation for local vehicle or NECA (non-selective adenosine receptor agonist) delivery. Representative histology (left bottom) showing NE sensor expression (green), DAPI (blue), and cannula tract. Scale bars, 200 μm (inset) and 1 mm. (B) PWT (far left), PWL (middle left), sucrose preference (middle right), and immobility time in TST (far right) across sham + Veh, sham + NECA, cancer + Veh, and cancer + NECA groups. NECA attenuated cancer-induced mechanical hypersensitivity, anhedonia, and despair-like behavior without affecting sham mice (*n* = 8–12 mice per group). (C) NE dynamics in the PPC during TST. Left, averaged ΔF/F (%) traces aligned to TST onset (dashed line); shaded areas represent SEM. Middle, trial-by-trial heatmaps for Pre-TST and TST periods. Right, mean ΔF/F (%) quantification during Pre-TST and TST epochs (*n* = 8 mice per group). (D) NE dynamics in the PPC during von Frey stimulation. Displayed as in (C). NECA restored blunted NE responses in cancer mice (*n* = 8 mice per group). (E) Relative mRNA levels of *Slc6a2*, *Adora2a*, and *Gng7* in the PPC from cancer mice treated with vehicle or CGS21680 (selective A2AR agonist) (*n* = 4 pooled samples per group, each pooled from 4 mice). (F) Representative western blots (left) and quantification (right) of NET protein levels normalized to β-tubulin in the PPC from cancer mice treated with vehicle or CGS21680. CGS21680 significantly reduced NET expression in cancer mice (*n* = 4 pooled samples per group, each pooled from 4 mice). In (B)–(D): ****p* < 0.001 for sham + Veh vs. cancer + Veh; ^#^*p* < 0.05, ^##^*p* < 0.01, ^###^*p* < 0.001 for cancer + Veh vs. cancer + NECA; ns, not significant for sham + Veh vs. sham + NECA. In (E) and (F): **p* < 0.05, ***p* < 0.01, ****p* < 0.001 for cancer + Veh vs. cancer + CGS21680; ns, not significant. Significance was assessed using two-way ANOVA with Bonferroni’s *post-hoc* test for PWT and PWL in (B), and for (C), (D), (E), and (F); one-way ANOVA with Bonferroni’s *post-hoc* test for SPT and TST in (B). Data are presented as mean ± SEM. Exact *p*-values, degrees of freedom, and pairwise comparisons are provided in the Source Data file. Created with BioRender.com.

**
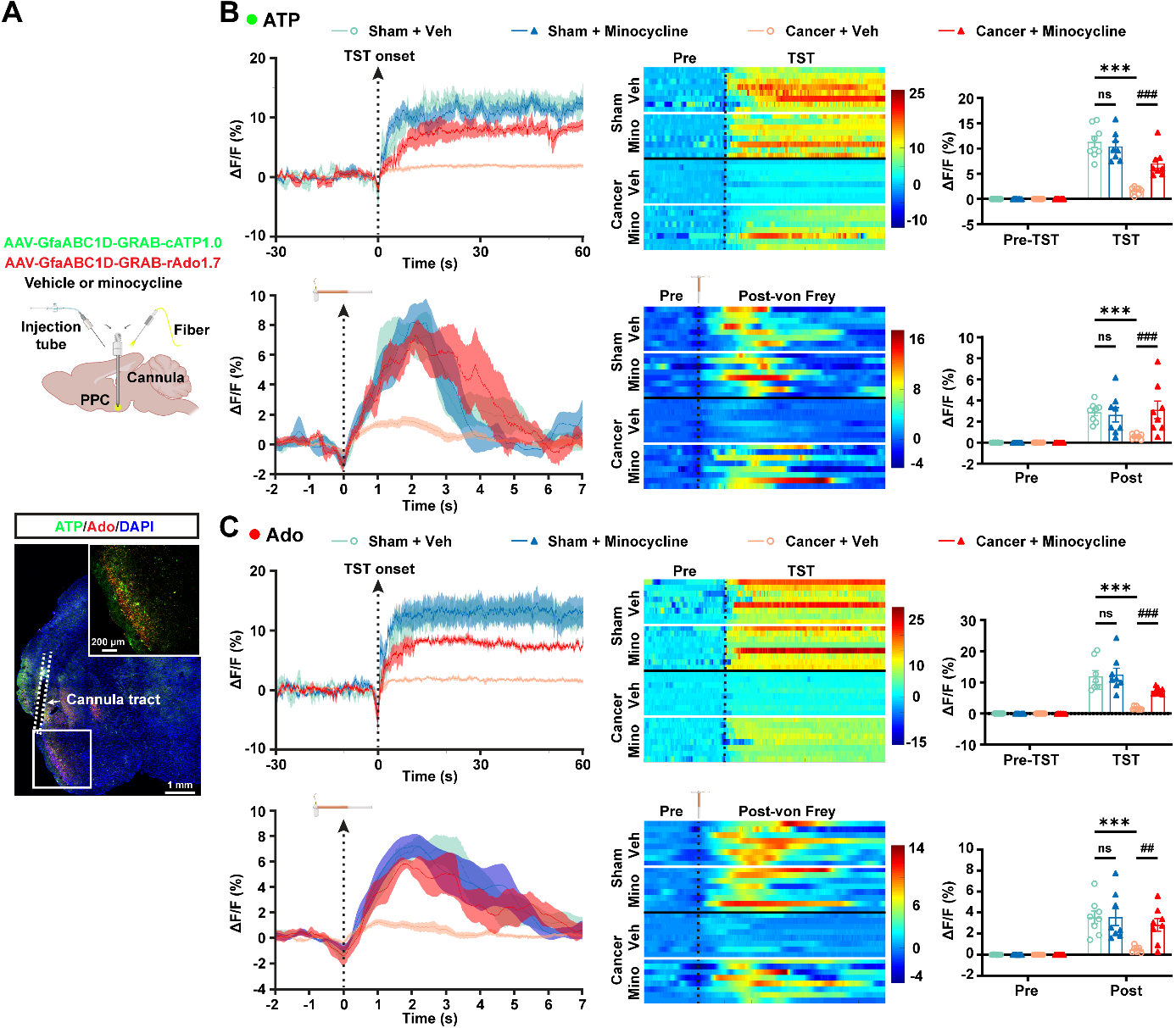
**

**Figure S9. Minocycline-mediated inhibition of astrocytic A1 polarization rescues PPC purinergic signaling deficits in cancer mice.** (A) Experimental design. AAV-GfaABC1D-GRAB-cATP1.0 and AAV-GfaABC1D-GRAB-rAdo1.7 were co-injected into the PPC with cannula implantation for local vehicle or minocycline (A1 astrocyte polarization inhibitor) delivery. Representative histology (bottom left) showing ATP sensor (green) and Ado sensor (red) expression with DAPI (blue) and cannula tract. Scale bars, 200 μm (inset) and 1 mm. (B) Astrocytic ATP dynamics in the PPC during TST (top) and von Frey stimulation (bottom). Left, averaged ΔF/F (%) traces aligned to TST onset or von Frey stimulation onset (dashed line); shaded areas represent SEM. Middle, trial-by-trial heatmaps for pre- and post-stimulus periods. Right, mean ΔF/F (%) quantification. Minocycline restored cancer-induced reductions in astrocytic ATP release (*n* = 8 mice per group). (C) Astrocytic Ado dynamics in the PPC during TST (top) and von Frey stimulation (bottom). Displayed as in (B). Minocycline similarly rescued Ado signaling deficits in cancer mice (*n* = 8 mice per group). In (B) and (C): ****p* < 0.001 for sham + Veh vs. cancer + Veh; ^##^*p* < 0.01, ^###^*p* < 0.001 for cancer + Veh vs. cancer + minocycline; ns, not significant for sham + Veh vs. sham + minocycline. Significance was assessed using two-way ANOVA with Bonferroni’s *post-hoc* test. Data are presented as mean ± SEM. Created with BioRender.com.

**
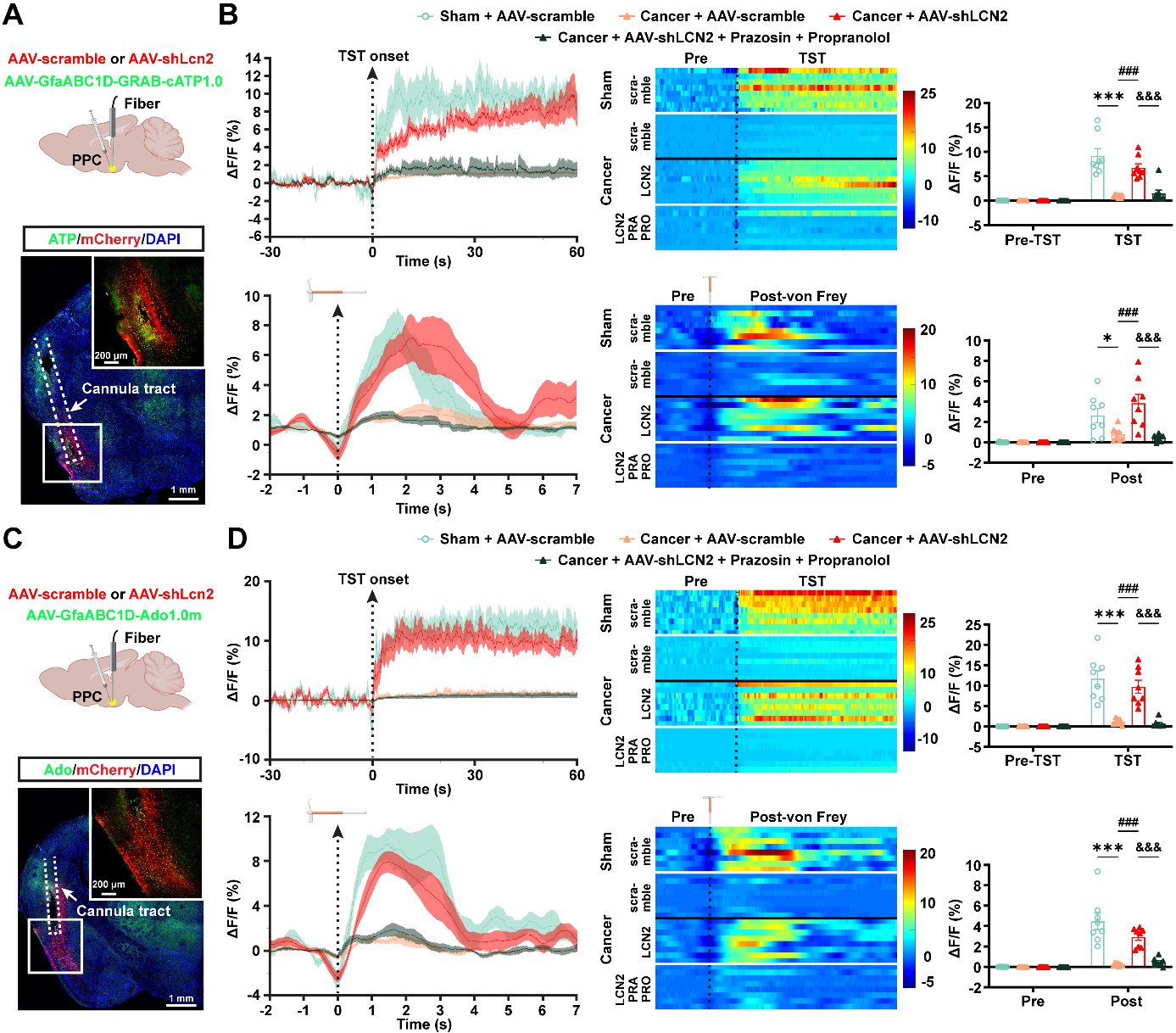
**

**Figure S10. *Lcn2* knockdown rescues PPC astrocytic purinergic signaling deficits in cancer mice via adrenergic receptor-dependent mechanisms.** (A) Experimental design for ATP recording. AAV-scramble or AAV-shLcn2 was co-injected with AAV-GfaABC1D-GRAB-cATP1.0 into the PPC with fiber implantation. Representative histology (bottom) showing ATP sensor (green), mCherry (red), and DAPI (blue) with cannula tract. Scale bars, 200 μm (inset) and 1 mm. (B) Astrocytic ATP dynamics in the PPC during TST (top) and von Frey stimulation (bottom) across sham + AAV-scramble, cancer + AAV-scramble, cancer + AAV-shLcn2, and cancer + AAV-shLcn2 + prazosin + propranolol groups. Left, averaged ΔF/F (%) traces aligned to TST onset or von Frey stimulation onset (dashed line); shaded areas represent SEM. Middle, trial-by-trial heatmaps for pre- and post-stimulus periods. Right, mean ΔF/F (%) quantification. *Lcn2* knockdown rescued cancer-induced ATP signaling deficits, an effect that was abolished by co-administration of prazosin and propranolol (adrenergic receptor antagonists) (*n* = 8 mice per group). (C) Experimental design for Ado recording. AAV-scramble or AAV-shLcn2 was co-injected with AAV-GfaABC1D-GRAB-rAdo1.0m into the PPC with fiber implantation. Representative histology (bottom) showing Ado sensor, mCherry, and DAPI with cannula tract. Scale bars, 200 μm (inset) and 1 mm. (D) Astrocytic Ado dynamics in the PPC during TST (top) and von Frey stimulation (bottom) across the same four groups as in (B). Displayed as in (B). *Lcn2* knockdown similarly restored Ado signaling, an effect that was again abolished by adrenergic receptor blockade (*n* = 8 mice per group). In (B) and (D): **p* < 0.05, ****p* < 0.001 for sham + AAV-scramble vs. cancer + AAV-scramble; ^###^*p* < 0.001 for cancer + AAV-scramble vs. cancer + AAV-shLcn2; ^&&&^*p* < 0.001 for cancer + AAV-shLcn2 vs. cancer + AAV-shLcn2 + prazosin + propranolol. Significance was assessed by two-way ANOVA with Bonferroni’s *post-hoc* test. Data are presented as mean ± SEM. Exact *p*-values, degrees of freedom, and pairwise comparisons are provided in the Source Data file. Created with BioRender.com.

**Table S1: virus**

| **Virus name** | **Abbreviation** | **Type (Serotype)** | **Titer (v.g./mL)** | **Supplier (City, Country）** |
| --- | --- | --- | --- | --- |
| rAAV-cfos-EYFP-WPRE-pA | AAV-cfos-EYFP | AAV2/phpeb | 5.40 × 10^12^ | BrainVTA (Wuhan, China) |
| rAAV-E-SARE-CreERT2-PEST-WPRE-hGH-pA | AAV-E-SARE-CreERT2 | AAV2/9 | 5.78 × 10^12^ | BrainVTA (Wuhan, China) |
| AAV-CAG-DIO-EYFP | AAV-DIO-EYFP | AAV2/9 | 1.49 × 10^13^ | Taitool (Shanghai, China) |
| rAAV-EF1α-DIO-EGFP-WPRE-hGH-pA | AAV-DIO-EGFP | AAV2/5 | 4.91 × 10^12^ | BrainVTA (Wuhan, China) |
| rAAV-GfaABC1D-Cre-WPRE-SV40-pA | AAV-GfaABC1D-Cre | AAV2/5 | 5.22 × 10^12^ | BrainVTA (Wuhan, China) |
| rAAV-GfaABC1D-cATP1.0 (chick)-WPRE-hGH-pA | AAV-GfaABC1D-cATP1.0 | AAV2/9 | 1.66 × 10^13^ | Taitool (Shanghai, China) |
| AAV-GfaABC1D-rAdo1.7 | AAV-GfaABC1D-rAdo1.7 | AAV2/9 | 2.89 × 10^12^ | BrainVTA (Wuhan, China) |
| AAV-GfaABC1D-Ado1.0m | AAV-GfaABC1D-Ado1.0m | AAV2/9 | 5.18 × 10^12^ | BrainVTA (Wuhan, China) |
| rAAV-hSyn-GRAB-NE2m (3.1)-WPRE-pA | AAV-hSyn-GRAB-NE2m | AAV2/9 | 5.40 × 10^12^ | BrainVTA (Wuhan, China) |
| rAAV-hSyn-GRAB-rNE1.0-WPRE-pA | AAV-hSyn-GRAB-rNE1.0 | AAV2/9 | 5.40 × 10^12^ | BrainVTA (Wuhan, China) |
| AAV-hSyn-FLEX-tdTomato-T2A-Synaptophysin-EGFP-WPRE-pA | AAV-FLEX-tdTomato-Synaptophysin-EGFP | AAV2/9 | 1.66 × 10^13^ | Taitool (Shanghai, China) |
| AAV-PRSx8-EGFP-WPRE-pA | AAV-Con | AAV2/9 | 1.43 × 10^13^ | Taitool (Shanghai, China) |
| rAAV-PRSx8-EGFP-P2A-mSlc6a2-WPRE-pA | AAV-NET | AAV2/9 | 2.51 × 10^12^ | Taitool (Shanghai, China) |
| rAAV-U6-shRNA1 (Lcn2)-CMV-mCherry-SV40-pA | AAV-shLcn2 | AAV2/9 | 2.05 × 10^12^ | BrainVTA (Wuhan, China) |
| rAAV-U6-shRNA (scramble)-CMV-mCherry-SV40-pA | AAV-scramble | AAV2/9 | 5.10 × 10^12^ | BrainVTA (Wuhan, China) |
| CTB-488 | CTB-488 | — | — | BrainVTA (Wuhan, China) |
| rAAV-EF1α-DIO-mCherry-WPRE-hGH-pA | RetroAAV-DIO-mCherry | AAV2 | 5.67 × 10^12^ | BrainVTA (Wuhan, China) |

**Table S2:** Primer information

| **Name** | **Direction** | **Sequence (5’–3’)** |
| --- | --- | --- |
| *Adora2a* | Forward | GCCATCCCATTCGCCATCA |
|  | Reverse | GCAATAGCCAAGAGGCTGAAGA |
| *Gng7* (*Gγ7*) | Forward | TCAGGTACTAACAACGTCGCC |
|  | Reverse | CAGTAGCCCATCAGGTCTGAC |
| *Slc6a2* (NET) | Forward | CATTGCCCTCTACGTTGGCTT |
|  | Reverse | ACACCACGCTCATAAAACTCTG |
| *Gapdh* | Forward | GGTGAAGGTCGGTGTGAACG |
|  | Reverse | CTCGCTCCTGGAAGATGGTG |
| GFP | Forward | CAGAAGAACGGCAТСAAGGC |
|  | Reverse | TGGGGCACAAGCTGGAGTA |
| mApple | Forward | CTTCAAGGTGCACATGGAGGGCTCC |
|  | Reverse | TGAAGCGCATGAACTCCTTG |

**Table S3: antibodies**

| **Antibody Type** | **Target** | **RRID** | **Dilution** | **Catalog Number** | **Supplier** |
| --- | --- | --- | --- | --- | --- |
| Primary | Rabbit anti-NET | AB_10687241 | 1:200 | AMT-002 | Alomone Labs |
| Primary | Rabbit anti-A2AR | AB_2039707 | 1:200 | AAR-002 | Alomone Labs |
| Primary | Rabbit anti-GNG7 | AB_2683527 | 1:700 | HPA057790 | Atlas Antibodies |
| Primary | Rabbit polyclonal anti-β-tubulin | AB_2210695 | 1:5000 | 10094-1-AP | Proteintech |
| Primary | Guinea pig anti-c-Fos | AB_2905595 | 1:1000 | 226308 | Synaptic Systems |
| Primary | Mouse monoclonal anti-NeuN | AB_2298772 | 1:1000 | MAB377 | Sigma-Aldrich |
| Primary | Mouse monoclonal anti-GFAP | AB_477010 | 1:1000 | G3893 | Sigma-Aldrich |
| Primary | Rabbit anti-GFAP | AB_2631098 | 1:400 | 12389S | Cell Signaling Technology |
| Primary | Guinea pig anti-GFAP | AB_2905596 | 1:1000 | 173308 | Synaptic Systems |
| Primary | Rabbit anti-Iba1 | AB_839504 | 1:500 | 019-19741 | Wako |
| Primary | Mouse monoclonal anti-C3 | AB_627277 | 1:50 | sc-28294 | Santa Cruz Biotechnology |
| Primary | Mouse monoclonal anti-S100A10 | AB_2717244 | 1:50 | MA5-24769 | Thermo Fisher Scientific |
| Primary | Rabbit monoclonal anti-S100A10 | AB_2183341 | 1:50 | NBP1-40588 | Novus Biologicals |
| Primary | Rabbit anti-tyrosine hydroxylase (TH) | AB_390204 | 1:200 | AB152 | Sigma-Aldrich |
| Primary | Goat polyclonal anti-mApple | AB_2895506 | 1:500 | AB5421 | SICGEN |
| Primary | Rabbit polyclonal anti-GFP | AB_221569 | 1:1000 | A-11122 | Thermo Fisher Scientific |
| Secondary | Goat anti-guinea pig 594 | AB_2905555 | 1:500 | ab150188 | Abcam |
| Secondary | Donkey anti-rabbit 488 | AB_2340619 | 1:1000 | 711-546-152 | Jackson ImmunoResearch |
| Secondary | Donkey anti-rabbit Cy3 | AB_2307443 | 1:1000 | 711-165-152 | Jackson ImmunoResearch |
| Secondary | Donkey anti-mouse Cy3 | AB_2340813 | 1:1000 | 715-165-150 | Jackson ImmunoResearch |
| Secondary | Donkey anti-mouse 488 | AB_2340846 | 1:1000 | 715-545-150 | Jackson ImmunoResearch |
| Secondary | Donkey Anti-Goat IgG H&L (HRP) | AB_3065024 | 1:2000 | ab205723 | Abcam |
| Secondary | Goat Anti-Rabbit IgG H&L (HRP) | AB_2819160 | 1:2000 | ab205718 | Abcam |

**Table S4: drugs and chemicals**

| **Name** | **Concentration**  **(**mM**)** | **Vehicle** | **Injection volume (unilateral, µL)** | **Catalog Number** | **Supplier** |
| --- | --- | --- | --- | --- | --- |
| ATP | 0.025 | ACSF | 1 | HY-B2176 | MedChemExpress |
| 5’-(N-ethylcarboxamido) adenosine (NECA) | 0.25 | DMSO ( stock solution); 0.9 % saline (working solution) | 0.3 | 119140 | Sigma-Aldrich |
| CGS21680 | 4 |  | 0.3 | HY-13201A | MedChemExpress |
| Prazosin | 6 |  | 0.2 | HY-B0193 | MedChemExpress |
| DSP-4 (hydrochloride) | 32 |  | 0.5 | HY-103210 | MedChemExpress |
| XEN2174 | 0.005 | 5 mM sodium acetate in 0.9 % saline | 0.4 | — | Provided by Yan Zhao, Chinese Academy of Sciences |
| Minocycline | 20.3 | ACSF | 0.3 | M9511 | Sigma-Aldrich |
